# Organised Genome Dynamics in the *Escherichia coli* Species Results in Highly Diverse Adaptive Paths

**DOI:** 10.1371/journal.pgen.1000344

**Published:** 2009-01-23

**Authors:** Marie Touchon, Claire Hoede, Olivier Tenaillon, Valérie Barbe, Simon Baeriswyl, Philippe Bidet, Edouard Bingen, Stéphane Bonacorsi, Christiane Bouchier, Odile Bouvet, Alexandra Calteau, Hélène Chiapello, Olivier Clermont, Stéphane Cruveiller, Antoine Danchin, Médéric Diard, Carole Dossat, Meriem El Karoui, Eric Frapy, Louis Garry, Jean Marc Ghigo, Anne Marie Gilles, James Johnson, Chantal Le Bouguénec, Mathilde Lescat, Sophie Mangenot, Vanessa Martinez-Jéhanne, Ivan Matic, Xavier Nassif, Sophie Oztas, Marie Agnès Petit, Christophe Pichon, Zoé Rouy, Claude Saint Ruf, Dominique Schneider, Jérôme Tourret, Benoit Vacherie, David Vallenet, Claudine Médigue, Eduardo P. C. Rocha, Erick Denamur

**Affiliations:** 1 Atelier de BioInformatique, Université Pierre et Marie Curie - Paris 6 (UPMC), Paris, France; 2 Microbial Evolutionary Genomics, Institut Pasteur, CNRS URA2171, Paris, France; 3 Faculté de Médecine, Université Paris 7 Denis Diderot, INSERM U722, Site Xavier Bichat, Paris, France; 4 Génoscope, Institut de Génomique, CEA, Evry, France; 5 Faculté de Médecine, Université Paris 5 René Descartes, INSERM U571, Paris, France; 6 Université Paris 7 Denis Diderot, Hôpital Robert Debré (APHP), EA 3105, Paris, France; 7 Plate-Forme Génomique, Institut Pasteur, Paris, France; 8 Laboratoire de Génomique Comparative, CNRS UMR8030, Institut de Génomique, CEA, Génoscope, Evry, France; 9 UR1077 Mathématique, Informatique, et Génome, INRA, Jouy en Josas, France; 10 Unité de Génétique des Génomes Bactériens, Institut Pasteur, CNRS URA2171, Paris, France; 11 UR888 Unité des Bactéries Lactiques et Pathogènes Opportunistes, INRA, Jouy en Josas, France; 12 Faculté de Médecine, Université Paris 5 René Descartes, INSERM U570, Paris, France; 13 Unité de Génétique des Biofilms, Institut Pasteur, CNRS URA2172, Paris, France; 14 Veterans Affairs Medical Center, Minneapolis, Minnesota, United States of America; 15 Department of Medicine, University of Minnesota, Minneapolis, Minnesota, United States of America; 16 Pathogénie Bactérienne des Muqueuses, Institut Pasteur, Paris, France; 17 Université Grenoble 1 Joseph Fourier, CNRS UMR 5163, Grenoble, France; Universidad de Sevilla, Spain

## Abstract

The *Escherichia coli* species represents one of the best-studied model organisms, but also encompasses a variety of commensal and pathogenic strains that diversify by high rates of genetic change. We uniformly (re-) annotated the genomes of 20 commensal and pathogenic *E. coli* strains and one strain of *E. fergusonii* (the closest *E. coli* related species), including seven that we sequenced to completion. Within the ∼18,000 families of orthologous genes, we found ∼2,000 common to all strains. Although recombination rates are much higher than mutation rates, we show, both theoretically and using phylogenetic inference, that this does not obscure the phylogenetic signal, which places the B2 phylogenetic group and one group D strain at the basal position. Based on this phylogeny, we inferred past evolutionary events of gain and loss of genes, identifying functional classes under opposite selection pressures. We found an important adaptive role for metabolism diversification within group B2 and *Shigella* strains, but identified few or no extraintestinal virulence-specific genes, which could render difficult the development of a vaccine against extraintestinal infections. Genome flux in *E. coli* is confined to a small number of conserved positions in the chromosome, which most often are not associated with integrases or tRNA genes. Core genes flanking some of these regions show higher rates of recombination, suggesting that a gene, once acquired by a strain, spreads within the species by homologous recombination at the flanking genes. Finally, the genome's long-scale structure of recombination indicates lower recombination rates, but not higher mutation rates, at the terminus of replication. The ensuing effect of background selection and biased gene conversion may thus explain why this region is A+T-rich and shows high sequence divergence but low sequence polymorphism. Overall, despite a very high gene flow, genes co-exist in an organised genome.

## Author Summary

Although abundant knowledge has been accumulated regarding the *E. coli* laboratory strain K-12, little is known about the evolutionary trajectories that have driven the high diversity observed among natural isolates of the species, which encompass both commensal and highly virulent intestinal and extraintestinal pathogenic strains. We have annotated or re-annotated the genomes of 20 commensal and pathogenic *E. coli* strains and one strain of *E. fergusonii* (the closest *E. coli* related species), including seven that we sequenced to completion. Although recombination rates are much higher than mutation rates, we were able to reconstruct a robust phylogeny based on the ∼2,000 genes common to all strains. Based on this phylogeny, we established the evolutionary scenario of gains and losses of thousands of specific genes, identifying functional classes under opposite selection pressures. This genome flux is confined to very few positions in the chromosome, which are the same for every genome. Notably, we identified few or no extraintestinal virulence-specific genes. We also defined a long-scale structure of recombination in the genome with lower recombination rates at the terminus of replication. These findings demonstrate that, despite a very high gene flow, genes can co-exist in an organised genome.

## Introduction

*Escherichia coli* was brought into laboratories almost a century ago to become one of the most important model organisms and by far the best-studied prokaryote. Major findings in phage genetics, bacterial conjugation, recombination, genetic regulation and chromosome replication involved the use of *E. coli*, especially laboratory derivatives of the K-12 strain, originally isolated from the faeces of a convalescent diphtheria patient in Palo Alto in 1922 [1]. However, K-12 derivatives are far from representing the whole *E. coli* species [2]. The primary habitat of *E. coli* is the lower intestinal tract of humans and other vertebrates, with which it typically establishes commensal associations. Healthy humans typically carry more than a billion *E. coli* cells in their intestine. It has been estimated that half of the living *E. coli* cells are outside their host, in their secondary habitat [3]. Beside these habitats, certain strains have the potential to cause a wide spectrum of intestinal and extra-intestinal diseases such as urinary tract infection, septicaemia, meningitis, and pneumonia in humans and animals [4]. Furthermore, *Shigella*, which have been elevated to the genus order with four species (*dysenteriae*, *flexneri*, *boydii*, *sonnei*) based on their capacity to generate a specific mucosal invasive diarrhoea strictly in humans and their biochemical characteristics, in fact belong to the *E. coli* species [5]–[7]. Of note, *Shigella* and enteroinvasive *E. coli* are considered the only obligate pathogens of the species, whereas other strains are facultative pathogens with a broad host range. Thus, natural isolates of *E. coli/Shigella* live in conditions quite different from those in the laboratory and must cope with very diverse environments that provide stresses ranging from immune system attack and protozoal grazing to starvation, low temperatures, and, more recently, antibiotic therapy.

With its large range of pathologies, *E. coli* is a major cause of human morbidity and mortality around the world. Each year *E. coli* causes more than two million deaths due to infant diarrhoea [8],[9] and extraintestinal infections (mainly septicaemia derived from urinary tract infection) [10], and is also responsible for approximately 150 million cases of uncomplicated cystitis [10]. Since humans and food animals carry so many *E. coli* cells that may establish commensal or antagonistic interactions with their hosts it is mandatory to define the genetic and population determinants that drive commensal strains to adopt a pathogenic behaviour.

Population genetic studies based on both multi-locus enzyme electrophoresis [11]–[13] and various DNA markers [14]–[18] have identified four major phylogenetic groups (A, B1, D and B2) and a potential fifth group (E) among *E. coli* strains. Strains of these groups differ in their phenotypic characteristics, including the ability to use certain sugars, antibiotic resistance profiles and growth rate–temperature relationships [19]. The distribution (presence/absence) of a range of virulence factors thought to be involved in the ability of a strain to cause diverse diseases also varies among strains of these phylogenetic groups [20]–[22], indicating a role of the genetic background in the expression of virulence [23]. Consequently, these groups are differently associated with certain ecological niches, life-history characteristics and propensity to cause disease. For example, group B2 and D strains are less frequently isolated from the environment [24], but more frequently recovered from extra-intestinal body sites [23]. While B2 strains represent 30 to 50% of the strains isolated from the faeces of healthy humans living in industrialised countries, they account for less than 5% in French Guyana Amerindians [25]–[26].

The clear clustering of *E. coli* strains into monophyletically meaningful groups has long been used as an argument favouring clonality within the species. However, analysis of gene sequences shows pervasive recombination, matching the well-known efficiency of conjugation and transduction of the species [17],[27]. Hence, it remains controversial whether such frequent recombination obliterates the phylogenetic signal. *E. coli* genomes show evidence of widespread acquisition of functions by horizontal gene transfer, concomitant with similar amounts of gene deletion [28]–[29]. While less than 3% of nucleotide divergence is found among conserved genes, the gene content between pairs of *E. coli* genomes may diverge by more than 30% [30]. Such diversification of gene content due to horizontal gene transfer contributes greatly to the diversity of the strains' phenotypes and can be accurately quantified only by the sequencing of a large number of strains to completion and closure.

Until now, sequencing efforts in *E. coli* have been focused mainly on pathogenic strains, particularly on diarrhoeal and group B2 extraintestinal pathogenic strains (see Table 1), precluding an unbiased assessment of the diversity of the species. Therefore, we have sequenced with high coverage and up to completion the genomes of 6 human-source *E. coli* strains. The *E. coli* strains were chosen to complement the available sequences and other ongoing sequencing projects (http://msc.jcvi.org/e_coli_and_shigella/index.shtml, http://www.sanger.ac.uk/Projects/Escherichia_Shigella/). They encompass two commensal strains of phylogenetic groups B1 and B2, a group B1 enteroaggregative strain, two group D urinary tract infection strains and a group B2 newborn meningitis strain (Table 1). We also sequenced the type strain of the closest *E. coli* relative, i.e., *E. fergusonii* [31], as an outgroup to permit accurate and meaningful evolutionary analyses with the 6 new *E. coli* genomes and the 14 other currently available *E. coli/Shigella* genomes. To statistically substantiate the identification of extraintestinal virulence-associated genes, we also applied a mouse lethality assay to the strains [32] to quantify the intrinsic virulence of the strain, excluding host variability and other potential confounding factors (Table 1). Our goal was to take the outstanding opportunity provided by the availability of many genomes of a single bacterial species, regarding which a considerable amount of knowledge has been accumulated over the years, to answer to the following questions. (i) Is there genome-wide evidence of frequent recombination and does it vary with genome location? (ii) If so, can one nonetheless infer an intra-specific bacterial phylogeny? (iii) How do the different factors of genome dynamics (mutation, horizontal gene transfer with or without recombination) result together in strain diversification? (iv) Is genome dynamics in conflict with genome organisation? (v) How does the commensalism/pathogenicity duality evolve?

**Table 1:** Principal characteristics of the 20 *Escherichia coli/Shigella* strains and 1 *E. fergusonii* strain.

Strains	Host	Sample	Clinical condition (Pathotype^a^)	Phylogenetic group^b^	Extraintestinal mouse model phenotype^c^ (Number of mice killed out of 10)	Genome sequence reference
K-12 MG1655	Human	Faeces	Commensal	A	NK (0)	[115]
K- 12 W3110	Human	Faeces	Commensal	A	NK (0)	Nara Institute of Science and Technology
**IAI1**	**Human**	**Faeces**	**Commensal**	**B1**	**NK (0)**	**This work**
**55989**	**Human**	**Faeces**	**Diarrhoea (EAEC)**	**B1**	**K (10)**	**This work**
*S. boydii* 4 227 (Sb 227)	Human	Faeces	Shigellosis	S1	ND^d^	[116]
*S. sonnei* 046 (Ss 046)	Human	Faeces	Shigellosis	SS	ND	[116]
*S. flexneri* 2a 301 (Sf 301)	Human	Faeces	Shigellosis	S3	ND	[117]
*S. flexneri* 2a 2457T (Sf 2457T)	Human	Faeces	Shigellosis	S3	NK (0)	[118]
*S. flexneri* 5b 8401 (Sf 8401)	Human	Faeces	Shigellosis	S3	ND	[119]
*S. dysenteriae* 1 197 (Sd 197)	Human	Faeces	Shigellosis	SD1	ND	[116]
O157:H7 EDL933	Human	Faeces	Diarrhoea (EHEC)	E	NK (1)	[120]
O157:H7 Sakai	Human	Faeces	Diarrhoea (EHEC)	E	NK (1)	[121]
**UMN026**	**Human**	**Urine**	**Cystitis (ExPEC)**	**D**	**K (10)**	**This work**
**IAI39**	**Human**	**Urine**	**Pyeloneprhitis (ExPEC)**	**D**	**K (8)**	**This work**
UTI89	Human	Urine	Cystitis (ExPEC)	B2	K (10)	[122]
APEC O1	Chicken	Lung	Colisepticemia (ExPEC)	B2	K (10)	[123]
**S88**	**Human**	**Cerebro-spinal fluid**	**New born meningitis (ExPEC)**	**B2**	**K (10)**	**This work**
CFT073	Human	Blood	Pyeloneprhitis (ExPEC)	B2	K (10)	[30]
**ED1A**	**Human**	**Faeces**	**Healthy subject**	**B2**	**NK (0)**	**This work**
536	Human	Urine	Pyeloneprhitis (ExPEC)	B2	K (10)	[124]
***E. fergusonii***	**Human**	**Faeces**	**Unknown**	**Outgroup**	**NK (1)**	**This work**

The strains in bold correspond to the strains sequenced in this work.

a EAEC (enteroaggregative *E. coli*), EHEC (enterohaemorrhagic *E. coli*), ExPEC (extraintestinal pathogenic *E. coli*).

b The *E. coli* and *Shigella* phylogenetic groups are as defined in [22] and [6], respectively.

c K, killer; NK, Non Killer [32].

d ND, not determined.

## Results/Discussion

### The General Features of the Seven Sequenced Genomes

We fully sequenced the chromosomes and the plasmids, if any, of 6 strains of *E. coli* and the reference type strain of *E. fergusonii*. The general features of these replicons are listed in Tables 2 and 3. Genomes were sequenced at an average of 12-fold coverage and were then finished. The 6 newly sequenced *E. coli* chromosomes contain between 4.7 Mb and 5.2 Mb each, corresponding to between 4627 and 5129 protein coding genes, slightly above the average value within the 20 genomes that we analyzed (∼4700 genes, ranging from 4068 to 5379). The chromosome of *E. fergusonii* is slightly smaller with ∼4.6 Mb and ∼4500 protein coding genes. The G+C content is very similar among the 6 strains and close to the *E. coli* K-12 MG1655 value (∼50.8%). The G+C content of *E. fergusonii* is lower at 49.9%. These chromosomes have similar densities of coding genes and numbers of stable RNA genes. By contrast, the number of pseudogenes varies more widely, from 22 in *E. fergusonii* to 95 in strain ED1a (Table 2). The list of pseudogenes is available in Table S1.

**Table 2:** General features of the *Escherichia coli* and *E. fergusonii* genomes sequenced in this work with *E. coli* K-12 MG1655 as reference (chromosome features).

Chromosome features	*E. coli* K-12 MG1655	*E. coli* strains						*E. fergusonii* ATCC
		55989	IAI1	ED1a	S88	IAI39	UMN026	
Genome Size (bp)	4 639 675	5 154 862	4 700 560	5 209 548	5 032 268	5 132 068	5 202 090	4 588 711
G+C content (%)	50.8	50.7	50.8	50.7	50.7	50.6	50.7	49.9
rRNA operons	7 (+5S)	7 (+5S)	7 (+5S)	7 (+5S)	7 (+5S)	7 (+5S)	7 (+5S)	7 (+5S)
tRNA genes	86	94	86	91	91	88	88	87
Total Protein-coding genes^a^	4306	4969	4491	5129	4859	4906	4918	4336
Pseudogenes^b^ (nb)	81	79	51	95	90	80	45	22
Protein coding density^c^	85.7	87.4	87.6	86.2	87	86.1	87.8	84.7
Assigned function^d^ (%)	80	74	77	74	77	78	76.5	77
Conserved hypothetical (%)	12.5	23	21.5	23	22	20	22	20
Orphans (%)	7.5	3	1.5	3	1	2	1.5	3
IS-like genes (nb)	66	150	42	118	47	224	92	29
Phage-associated genes (nb)	231	406	201	657	507	393	429	235

a The number of protein-coding genes is given without the number of coding sequences annotated as artifactual genes (Supplementary Table 2A).

b The number of pseudogenes computed for each genome corresponds to the real number of genes that are pseudogenes: one pseudogene can be made of only one CDS (in this case the gene is partial compared to the wild type form in other *E. coli* strains) or of several CDSs (generally two or three CDSs corresponding to the different fragments of the wild type form in other *E. coli* strains). These lists of pseudogenes are available in Supplementary Table 1.

c The computed protein coding density takes into account the total length of protein genes excluding overlaps between genes, artifacts, and RNA genes.

d Protein genes with assigned function include the total number of definitive and putative functional assignments.

**Table 3:** General features of the *Escherichia coli* and *E. fergusonii* genomes sequenced in this work with *E. coli* K-12 MG1655 as reference (plasmid features).

Plasmid features	*E. coli* strains					*E. fergusonii* ATCC
	55989	ED1a	S88	UMN026		
Genome Size (bp)	72 482	119 594	133 853	122 301	33 809	55 150
G+C content (%)	46.1	49.2	49.3	50.5	42	48.5
Total Protein-coding genes^a^	100	150	144	149	49	54
Pseudogenes^b^ (nb)	7	11	9	8	0	5
Protein coding density^c^	75.6	86.2	87	79.4	87.5	88.7
Assigned function^d^ (%)	74	53	65	65.7	35.4	46.6
Orphans (%)	17	31.5	25.8	27.8	12.5	20.7
Hypothetical (%)	9	15.5	9.2	6.5	52.2	32.7
IS-like genes (nb)	18	14	14	15	0	4

a The number of protein-coding genes is given without the number of coding sequences annotated as artifactual genes (Supplementary Table 2A).

b The number of pseudogenes computed for each genome corresponds to the real number of genes that are pseudogenes: one pseudogene can be made of only one CDS (in this case the gene is partial compared to the wild type form in other *E. coli* strains) or of several CDSs (generally two or three CDSs corresponding to the different fragments of the wild type form in other *E. coli* strains). These lists of pseudogenes are available in Supplementary Table 1.

c The computed protein coding density takes into account the total length of protein genes excluding overlaps between genes, artifacts, and RNA genes.

d Protein genes with assigned function include the total number of definitive and putative functional assignments.

The variation in the number of pseudogenes is uncorrelated with the number of transposable elements and phage-associated genes, which vary in the range 42–224 and 201–517 respectively. While some phage-associated genes are scattered throughout the chromosomes, the majority are concentrated in well-defined prophage regions. Analyses of the prophages suggest that many may still be functional. These prophages often carry at their extremity some unrelated cargo genes that probably arose from genomes of previously infected bacteria, as found in *Salmonella* [33]. We sequenced a total of 6 plasmids, varying in size from 34 to 134 kbp: four strains possess one plasmid each whereas one strain has 2 plasmids (Table 3). As frequently noted, the plasmids have a lower gene density (84%, vs. 87% for chromosomes), lower G+C content (47.4%, vs. 50.7% for chromosomes) and more pseudogenes (2.7%, vs. 1.5% for chromosomes). The percentage of orphan proteins (i.e., having no detectable homolog in other organisms) is also high on plasmids (6.5 to 52.2%), while it ranges between 1–3% on the chromosomes.

A manual expert annotation of the new *E. coli* strains was performed on genes and regions not found in *E. coli* K-12 MG1655 (about 10 000 genes in total; Table S2A). This allowed the re-annotation of orthologs in the previously available *Escherichia* and *Shigella* genomes (see Materials and Methods). The annotation data, together with the results of the comparative analysis were stored in a relational database called ColiScope, which is publicly available using the MaGe Web-based interface at http://www.genoscope.cns.fr/agc/mage. This re-annotation process revealed extensive variations in the number of the newly predicted genes (Table S2B). For example, between the two strains of *E. coli* O157:H7 we found twice as many newly predicted genes in one strain as in the other. In some genomes important genes were missing. For example, in *E. coli* APEC O1 several subunits of the ribosome, DNA polymerase III, and ATP synthase were missing in the original annotation (Table S3, *E. coli* APEC sheet). In other genomes, the re-annotation allowed us to standardise the definition and identification of pseudogenes. For example, in *S. sonnei* Ss 046 most of the newly annotated genes correspond to insertion sequences (ISs) and small fragments of incompletely annotated pseudogenes (Table S3, *S. sonnei* sheet). As a result of this effort, the present ColiScope database contains a complete and consistent set of annotations for the 7 newly sequenced genomes and the 14 available *Escherichia* and *Shigella* genomes. These data were the starting point of the work presented here.

We analyzed gene order conservation within the 21 genomes (Table S4). More than half of the genomes have exactly the gene order of *E. coli* K-12 MG1655, which we inferred as ancestral. Thus, the organisation of the core genome is stable in most strains. Three genomes show 1 or 2 rearrangements. Seven genomes show more than 10 blocks of synteny: 6 of these genomes are from *Shigella*, the high rearrangement rates of which resulted in up to 65 blocks of synteny in *S. dysenteriae*. These genomes have a large number of ISs, ranging from 549 to 1155 in *S. flexneri* and *S. dysenteriae*, respectively, which are well known to shuffle genomes. *E. fergusonii* also shows a large number of rearrangements relative to the ancestral organization of the *E. coli* genome. Since the organisation of some strains of the more distantly related *Salmonella enterica* closely resembles that of *E. coli* K-12 MG1655, many rearrangements must have taken place in the branch leading to *E. fergusonii*.

Figure S1 provides the classical concentric circle representation for the 7 genomes we sequenced, showing GC skews, G+C variation, and a description of the presence of genes in ever-increasing clades within the genus, relative to the inferred ancestral genome. The first position of the sequences was chosen to match the orthologous region in the *E. coli* K-12 MG1655 genome and corresponds to the intergenic region between *lasT* and *thrL*. Origins and termini of replication were identified by GC skews and homology with the respective *E. coli* K-12 MG1655 regions. These figures show that divergence from the average G+C content often occurs in genomic regions absent in the other strains. They also reveal the highly mosaic structure of these genomes, comprising the core genes and the accessory genes, which we then set out to quantify.

### The Core and Pan-Genomes of *E. coli*

The analysis of the first *E. coli* genomes changed our views about the evolution of gene repertoires in bacteria. Genomes within the species vary in size by more than 1 Mb, i.e., by more than 1000 genes, and even the gene repertoires of similarly sized genomes differ widely [30],[34]. We have thus taken advantage of the unprecedented availability of 20 completely sequenced genomes of the same species to analyse the evolution of the gene repertoire. We first identified the core and pan-genomes of *E. coli*, i.e., the genes present in all genomes and the full set of non-orthologous genes among all genomes. In our data set, the average *E. coli* genome contains 4721 genes, the core genome contains 1976 genes, and the pan-genome contains 17 838 genes. The random sampling of one gene within a randomly selected *E. coli* genome has a probability of only ∼42% of revealing a ubiquitous gene. On the other hand, the full sequencing of an *E. coli* strain allows observation of only one-fourth of the observed pan-genome. This implies that although some fundamental functions can be well studied by using a model strain, no single strain can be regarded as highly representative of the species.

**Figure 1:**
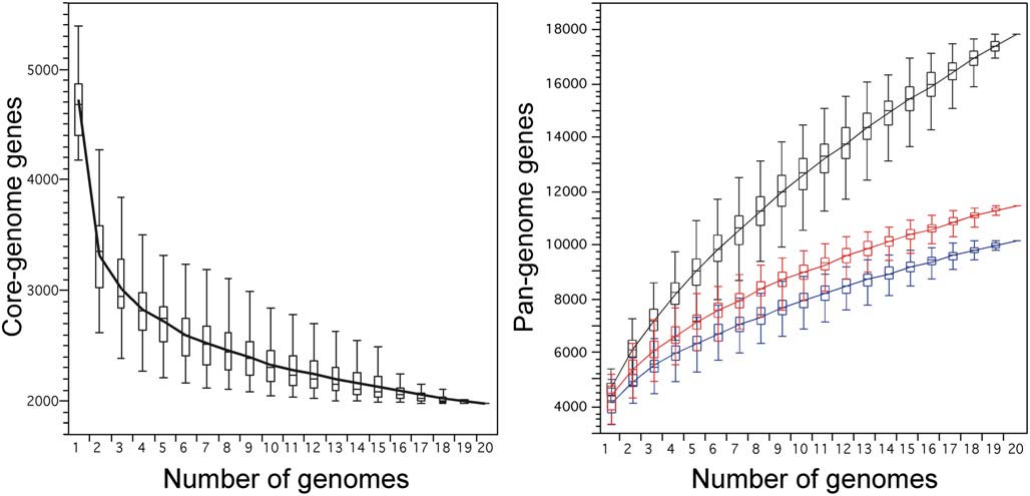
*Escherichia coli* core and pan-genome evolution according to the number of sequenced genomes. Number of genes in common (left) and total number of non-orthologous genes (right) for a given number of genomes analysed for the different strains of *E. coli*. The upper and lower edges of the boxes indicate the first quartile (25th percentile of the data) and third quartile (75th percentile), respectively, of 1000 random different input orders of the genomes. The central horizontal line indicates the sample median (50th percentile). The central vertical lines extend from each box as far as the data extend, to a distance of at most 1.5 interquartile ranges (i.e., the distance between the first and third quartile values). At 20 sequenced genomes, the core-genome had 1976 genes (11% of the pan-genome), whereas the pan-genome had (i) 17 838 total genes (black), (ii) 11 432 genes (red) with no strong relation of homology (<80% similarity in sequence), and (iii) 10 131 genes (blue) after removing insertion sequence-like elements (3834, 21% of all genes) and prophage-like elements (3873, 22% of all genes).

Further sampling of *E. coli* genomes is unlikely to change significantly the estimate of the core genome, however, the pan-genome is far from being fully uncovered (Figure 1). Annotation and sequencing artefacts may affect the estimations of core and pan-genome sizes, e.g. by spurious annotation of small genes or pseudogenes. We hope to have minimised such problems by using a coherent set of annotations. Still, we found that 40 genes deemed essential in *E. coli* K-12 W3110 [35] were missing in the core genome. Among these, 17 correspond to genes with conflicting reports of essentiality, or contextually essential genes such as prophage repressors, and are absent in most genomes. The other 23 genes have orthologs in most genomes and 19 are missing in a single genome where they can be found as pseudogenes interrupted by a single-nucleotide frameshift. While “pseudogenisation” does often start with such frameshifts [36], these genes correspond to core housekeeping functions, so the reported frameshifts probably represent sequencing errors. For example, it is hard to see how *S. boydii* could replicate without the catalytic α-subunit of the DNA polymerase III or how *E. coli* 536 could survive without a tyrosine tRNA synthetase. We found some comfort in verifying that none of the 23 genes was absent from the 7 genomes we sequenced. If one assumes that these essential genes cannot be deleted and that no special care has been taken to check for sequencing errors at these loci, then our estimation of the core genome should be increased by a factor of 260/(260-23) to 2167 genes. This still makes the core genome less than half of the average *E. coli* genome (∼46%). Importantly, no gene of the core genome, nor any operon ubiquitous in *E. coli*, was unique to the species, i.e., we could always find a homolog in at least one of the other fully sequenced bacterial genomes.

Some elements recently amplified in the genome, such as transposable elements, create multiple copies that are not orthologs *sensu strictu*, even though they probably have the same function. They will thus inflate the size of the pan-genome by increasing the number of strain-specific genes. We therefore made two complementary analyses. First, we classed together all paralogs with more than 80% sequence similarity. This led to 11 432 genes of a functionally diverse pan-genome (Figure 1). Second, we removed all transposable elements and prophages, but not their cargo genes, from the pan-genome to obtain a set of 10 131 genes. These analyses still lead to a vast pan-genome for the species and show that its large size is not a simple consequence of the presence of selfish genes or recent amplifications of genetic material. They also show that further sampling of *E. coli* genomes is likely to uncover a significant number of currently unrecognised genes that may confer lasting adaptive value for the diversification of the species.

**Figure 2:**
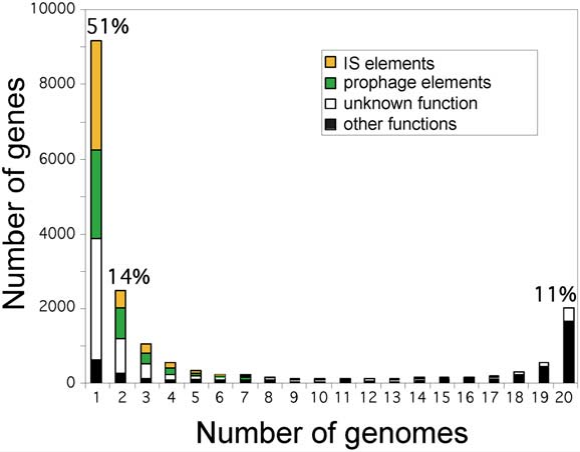
Frequency of genes within the 20 analysed *Escherichia coli* genomes. At one extreme of the x-axis are the genes present in a single genome which are regarded as strain specific genes (9054 genes: 51% of the pan-genome), while at the opposite end of the scale are situated the genes found in all 20 genomes, which represent the *E. coli* core-genome (1976 genes: 11% of the pan-genome). Coloured rectangles represent the proportion of insertion sequence (IS)-like elements (yellow), prophage-like elements (green), and genes of unknown/unclassified function (white). Black rectangles represent genes for which a function can be assigned. Strain-specific genes correspond to 2885 IS-like elements (32%), 2352 prophage-like elements (26%), and 3220 genes of unknown/unclassified function (35%).

Progressive sampling of *E. coli* genomes will tend to reduce the core genome to the list of essential genes because only instantaneously lethal deletions will never be found in natural populations of living cells. Hence, it is more relevant to quantify the relative frequency of each gene of the pan-genome among extant genomes (Figure 2). Of the genes in an average *E. coli* genome, approximately 62% are present in at least 18 genomes, and thus might be called the persistent genes [37], while 26% exist in 4 or fewer genomes, and thus might be called the volatile genes. Thus, most genes of the pan-genome exist in very few (≤20%) or almost all (≥90%) of the genomes, leaving only a small subset of genes that are present in around half of the genomes. The functional pattern of these groups of genes varies. Genes of known function are strongly over-represented among persistent genes, whereas genes of unknown function and especially selfish DNA, such as transposable and prophage elements, are over-represented among strain-specific (volatile) genes (Figure 2). Although some of these strain-specific genes may confer adaptive functions that allow the exploration of new niches (see below the section on the genome repertoire dynamics), the volatility of this set and the functions thereby over-represented suggest that most such genes are non-adaptive.

We assessed how different was *E. fergusonii* from the strains of *E. coli*. We computed the core genome of the 21 genomes (20 *E. coli*+1 *E. fergusonii*), which contained 1878 genes. We then made experiments in which we computed the core genome of all combinations of 20 genomes and then added the 21^st^ at the end. We ranked the genomes in terms of which led to the highest decrease in the core genome size. *S. dysenteriae* (174 genes) led to the greatest reduction in the core genome, followed by *E. fergusonii* (98 genes). We then repeated the experiment with the pan-genome. In this analysis, we also found that the most contributory 21^st^ genome was *S. dysenteriae* (1434 genes), followed by *E. fergusonii* (984 genes). However, this results from the large number of ISs in the former strain. When we computed the pan-genome while merging together paralogs that are more than 80% identical, we found that *E. fergusonii* ranks first (709 genes), well ahead of the second place strain (*E.coli* CFT073 with 462 genes). This latter difference matches the phylogenetic distance of *E. fergusonii*, but the overall analysis shows that crossing the *E. coli* species barrier does not lead to dramatic changes in the core and pan-genome.

### Gene Conversion Is Frequent, but Not Enough So to Obscure the Phylogenetic Signal

Horizontal transfer of new genes necessarily entails different phylogenies for these genes, but has few implications for the inference of phylogeny in the core genome. However, a considerable fraction of the large amounts of DNA that seemingly enter *E. coli* cells is expected to arise from consepecifics or closely related species. Such DNA can integrate into the chromosome by homologous recombination and thus lead to allelic replacements that obscure the phylogenetic signal. To address this question, we first estimated the rate of recombination in the genomes, then tested whether such a rate could affect the phylogenetic reconstruction.

Using methods based on the coalescent framework, it is possible to estimate the ratio of recombination to mutation rates, i.e., to compare the probability of a recombination being initiated at a particular nucleotide with the probability of a mutation occurring at that same nucleotide. We analyzed each core gene with LDHat, a coalescent-based estimator of recombination [38], and estimated an average ratio of recombination to mutation close to 1.0 (data not shown). Classical population genetics models, such as the one used in LDHat, assume that recombination occurs through reciprocal exchange of DNA with a single crossover. In prokaryotes, incoming DNA sequences are short and the recombination process is akin to gene conversion, whereby linkage between two close regions may be weaker than between two distant ones if one of the former has engaged in conversion with incoming DNA. Bacterial genetic exchange does not always imply mechanisms strictly analogous to those involved in eukaryotic gene conversion. However, since we are concerned more with the signature of gene conversion in linkage disequilibrium than with the underlying molecular mechanisms, we will use the term gene conversion hereafter to refer generically to bacterial genetic exchanges. We took advantage of the peculiar signature of gene conversion on linkage disequilibrium [39] to estimate the per-base rates of mutation (theta) and gene conversion (C_gc_), as well as the average tract length (L_gc_) (assuming a geometrical distribution), with Approximate Bayesian Computation method [40],[41] (see model in Materials and Methods). We applied the method to individual genes of the core genome and to 3 kbp sliding windows along the whole genome multiple alignment (see Materials and Methods, Figure S2).

Both analyses provided similar average values, but since the genes differ widely in size, we preferred to use the genome alignment for the rest of the analyses. The average ratio of gene conversion to mutation (C_gc_/theta) was 2.47±0.05. The average tract length was very short: 50 bp on average, lower than our previous estimate of 120 bp based on multi-locus sequence typing (MLST) data [42], and lower than expected based on experimental data [43]. Contrary to expectations based on random experiments (see Materials and Methods), we observed a strong negative correlation (Pearson r = −0.55, p<0.001) between the ratio of recombination to mutation and the length of the conversion fragments. This may be explained by the overlap of gene conversion fragments in regions of high rate of exchange, which results in artificially low values of L_gc_, lending further support to the existence of high conversion rates in the population. In any case, these tract lengths should not necessarily be equated with the size of incoming DNA fragments.

Our model assumes a homogenous population. However, in the gut of a vertebrate, the most likely neighbour for a cell probably is another cell from the same clone, since mucus provides a structured environment within which sister cells are likely to stay together for some time. Transfers between such closely related strains are less affected by restriction [43] or divergence [44]. Every time such a transfer overlaps with a previous transfer from a distant clone it will effectively remove some trace of recombination and, thus, lead to a lower observed tract length. In spite of such limitations we find that a gene conversion event is twice as likely as a mutation to occur at a given position. Therefore, taking into account the estimated tract length (50 bp), a base is 100 times more likely to be involved in a gene conversion than to be involved in a mutation. This is twice as large as the classical estimate [27].

**Figure 3:**
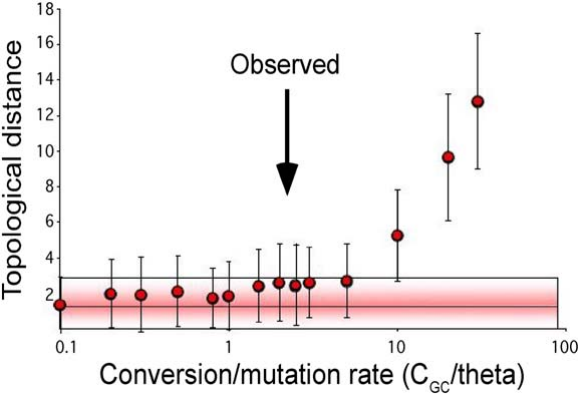
Impact of gene conversion rate on phylogenetic reconstruction. Sets of 20 sequences of 25 kbp were simulated 100 times under different gene conversion rates with constant tract length (50 bp) and mutation rate. The topology of the “true” genealogy of the sample (as inferred from a single nucleotide on which no gene conversion was allowed) was compared, using Robinson and Foulds distance, to the topology inferred from phylogenetic tree reconstruction using the simulated sequences. Error bars indicate one standard deviation variance, and horizontal bars represent one standard deviation variance from the no-gene-conversion model. A high rate of gene conversion is required to affect the topology of the reconstructed phylogeny. The observed average ratio of gene conversion to mutation (C_GC_/theta) is indicated by an arrow.

Is such a rate of gene conversion compatible with a meaningful phylogeny? If we do not consider the specificities of bacterial genetic exchange, the answer is no. The estimates provided under a simple crossing-over model are incompatible with any phylogenetic approach (data not shown). However the answer might be different if one considers that exchange in bacteria results in gene conversion. To test this idea quantitatively, we made coalescent simulations in which we used the parameters estimated previously (theta = 0.014 and L_gc_ = 50) and various rates of gene conversion to mutation (100 experiments for each value) to simulate the evolution of 25 kbp sequences (see Materials and Methods). We then compared the tree inferred by maximum likelihood with the tree derived directly from the simulated history, which reflects the history of the chromosomal background. We compared the tree topologies with Robinson and Foulds distances [45] and the SH, KH and ELW tests (see Materials and Methods). The average distance between the topologies of the pair of trees only starts to increase for gene conversion to mutation ratios (C_gc_/theta) much higher than the observed value (Figure 3). Hence, surprisingly, the substantial level of gene conversion in *E. coli* is not expected to blur the phylogenetic signal, and a meaningful and robust tree topology can be extracted from the sequences.

### The Phylogenetic History of the Strains

The foregoing analysis suggests that phylogenetic approaches can be used to analyse genome evolution even within highly non-clonal prokaryotic species. We therefore characterised the phylogenetic relationships among the 20 fully sequenced strains and the outgroup, using a maximum likelihood approach on all 1878 genes of the *Escherichia* core genome (i.e., the genes present in all 20 *E. coli/Shigella* and *E. fergusonii*), either independently or concatenated (1 769 508 nt, 88 883 informative sites). The same analysis was also performed on the chromosomal backbone using the *E. coli/Shigella* multiple genome alignment (2 672 618 nt, 115 435 informative sites) that, in addition, integrates non-coding sequences and pseudogenes.

**Figure 4:**
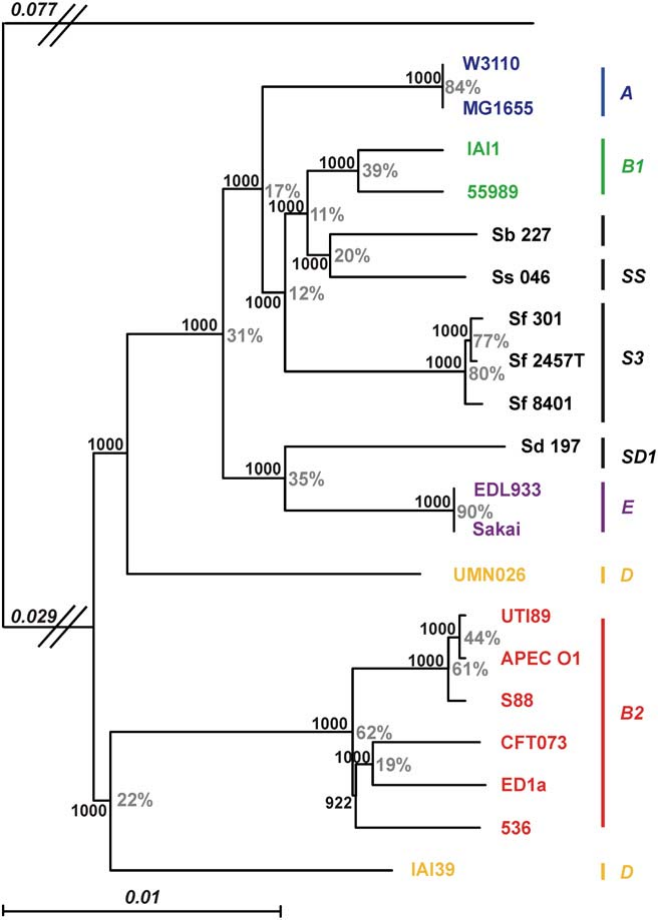
Maximum likelihood phylogenetic tree of the 20 *Escherichia coli* and *Shigella* strains as reconstructed from the sequences of the 1878 genes of the *Escherichia* core genome. The earliest diverging species, *E. fergusonii*, was chosen to root the tree. The numbers at the nodes correspond, in black, to the bootstrap values (1000 bootstraps) and, in grey, to a “consensus strength”, which is the number of genes that confirms the bipartition (see Materials and Methods). The latter value is displayed only in instances where consensus and tested trees correspond. The branch length separating *E. fergusonii* from the *E. coli* strains is not to scale; the numbers above the branch indicate its length. Phylogenetic group membership of the strains is indicated with bars at the right of the figure.

Using the concatenated genes of the core genome and a maximum likelihood approach, regardless of the method used to estimate a model (see Materials and Methods) we obtained a robust phylogeny with very high bootstrap values (Figure 4). When each of the 1878 individual gene phylogenies is compared to the concatenated gene phylogeny using various tree topology comparison tests (see Materials and Methods), about 25% are not significantly different from the concatenated gene tree. (It is worth noting that these tests are very stringent, as tree topologies differing by a single strain position can be significantly different.) Similarly, when the “consensus strength” of a node is defined as the percentage of genes that supports the bipartition at a specific node using CONSENSE, it can be shown that nodal consensus strength varies greatly, from 11% to 90% (Figure 4). However, in both approaches (tree topology comparison tests and consensus strength), the low values are largely due to an absence of phylogenetic signal differentiating the strains rather than to conflicting phylogenies, as 55% of genes have fewer than 40 informative sites (data not shown). All the classical groups described by multi-locus enzyme electrophoresis [13] and retrieved later on by genetic markers [14]–[18] are recovered as monophyletic apart from group D. The monophyly of group D in previous MLST studies never appeared to be very robust [16],[17],[46] and was presumably due to long-branch attraction. One D strain (IAI39) is closely related to the group B2 strains and belongs to the ECOR 35, 40, 41 subgroup [16],[46], whereas the other (UMN026), which belongs to the ECOR 46, 47, 49, 50 subgroup [16],[46], has emerged later. Our analysis retrieves the previously reported polyphyly of *Shigella* [6],[7]. Identical data were observed when using the multiple genome alignment (Figure S3), thus confirming the robustness of the phylogeny. A controversy has emerged about the more basal group within the *E. coli* species phylogeny, which some authors maintain is group B2 [16], [47]–[49] whereas others remain unconvinced [17],[46]. Our large data set using the closely related *E. fergusonii* as an outgroup, and thus avoiding the long-branch attraction artefact caused by the inclusion of *Salmonella* in some previous works, clearly shows that the first split in the *E. coli/Shigella* phylogenetic history leads on one hand to the strains of group B2 and a subgroup within group D, and on the other hand, to the remaining strains of the species. Groups A and B1, as well as the S1, S3 and SS *Shigella* groups, have emerged more recently (Figure 4).

**Figure 5:**
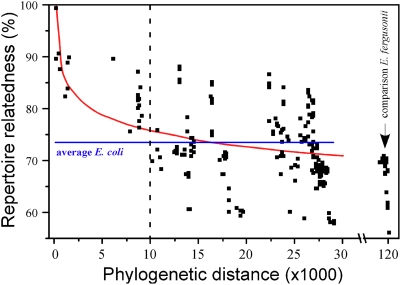
Association between gene repertoire relatedness and phylogenetic distance. The horizontal line corresponds to the average relatedness among *Escherichia coli/Shigella* strains. The log fit shows an R^2^ = 0.26 (p<0.01), which drops to R^2^ = 0.07 (p<0.01) if the points before the dashed line are removed.

Since lateral transfer is extensive in *E. coli*, we investigated how well gene repertoire relatedness fades with increasing evolutionary distance. We defined gene repertoire relatedness between two genomes as the fraction of shared orthologs in the smallest genome [50], and obtained the evolutionary distance from the phylogenetic tree in Figure 4. We found a negative association between the relatedness of gene repertoires and phylogenetic distance (Figure 5, R^2^ = 0.26, p<0.001). For very closely related genomes the association is quite clear (Spearman's *ρ* = −0.70, p<0.001, for the 12% closest comparisons corresponding to 2 of the 6 histogram bins of Figure S4). However, the more distant comparisons show much weaker association between relatedness and divergence time (Spearman's *ρ* = −0.30, p<0.001). Therefore, the number of shared orthologs is a poor phylogenetic marker and only among the most closely related genomes is there a high degree of similarity according to the repertoire of non-core genes of the pan-genome. This rapid saturation of phylogenetic signal in terms of gene repertoire relatedness might seem surprising in light of the ∼2000 genes shared among all genomes. Yet, if most gene deletions correspond to recent insertions, as we suggested previously, then the saturation of the phylogenetic signal results from the very small number of ancient acquisitions that are maintained among distant genomes. This effect is further enhanced by the frequent re-acquisition of some gene families such as phage and IS-associated genes. As a result, variance in gene repertoire relatedness increases quickly with phylogenetic distance to such an extent that some distantly related genomes actually exhibit greater gene repertoire relatedness than do more closely related ones.

### Reconstruction of Ancestral Genomes

**Figure 6:**
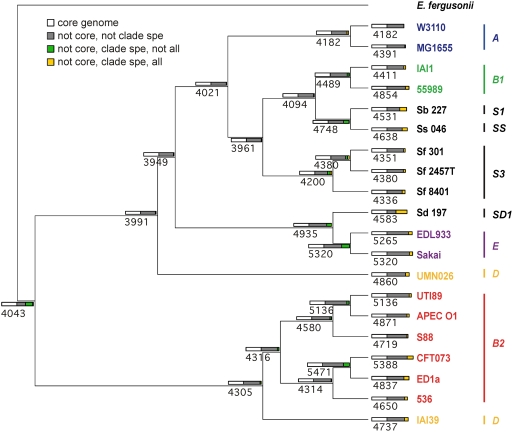
Inferred gene content evolution in the lineage of *Escherichia coli*. The cladogram shows the phylogenetic relationships among the 20 *E. coli/Shigella* genomes rooted on the *E. fergusonii* genome, as in Figure 4, but ignoring branch lengths. The major phylogenetic groups are indicated by the vertical lines. Each strain and internal node of the tree is labelled with the number of genes present (as inferred by maximum likelihood: see Materials and Methods). Coloured rectangles represent different gene classes within the gene repertoires of ancestral and modern *E. coli*. Rectangle widths are proportional to the number of genes. The four different gene classes (by colour) include genes that are: in the core genome (white), not clade-specific (grey), clade-specific but not ubiquitous in the clade (green) and both clade-specific and ubiquitous in the clade (yellow). A clade-specific gene is one that is inferred to be present only in the node and its descendent nodes.

**Figure 7:**
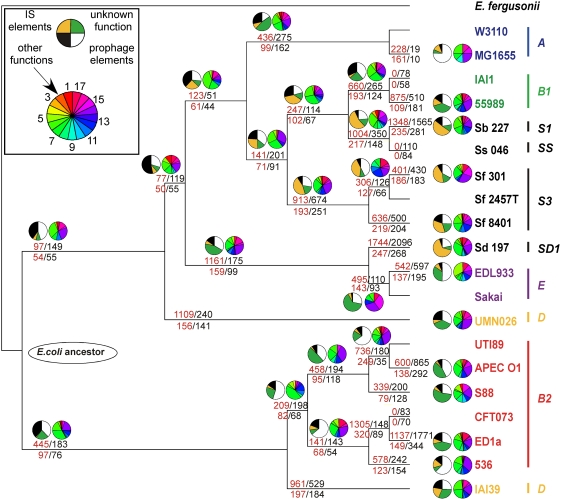
Reconstruction of gains and losses of genes in the evolution of *Escherichia coli*. The cladogram shows the phylogenetic relationships among the 20 *E. coli/Shigella* genomes rooted on the *E. fergusonii* genome, as in Figure 4, ignoring branch lengths for clarity. Each strain and internal node of the tree is labelled with the inferred numbers of genes gained (red: top) and lost (black: top) and the inferred numbers of corresponding events of gene acquisition (red: bottom) and loss (black: bottom) along the branch. Pie charts on each branch represent the functional classification of genes gained based on the colour-scale (details in the keys). The functional classes of known function genes are represented by numbers explained by a key in Supplementary Table 5. A similar figure, but displaying the pie charts for genes lost in the branch, is given in supplementary material (Figure S5).

The finding of a strong, reliable phylogenetic tree for the strains allows the inference of gene repertoire dynamics along the history of the species (Figures 6 and 7, Figure S5). We inferred the presence/absence of genes by maximum likelihood using the reference phylogeny at each ancestral node, including the inferred ancestor of all *E. coli*. We then quantified the flux of incoming and outgoing genes between consecutive nodes of the tree, i.e., at every branch, and inferred the associated change in genome length. There is a difference of almost one thousand genes between the gene repertoire we can infer reliably in the ancestor (4043 genes) and the expected one given the inferred genome length (∼5000). This is because most incoming genes are quickly lost. Anciently acquired volatile genes with no lasting adaptive value have been purged, if not re-acquired later on, whereas recently acquired ones may still persist in populations. Indeed, the gap between expected and inferred gene numbers increases linearly with the distance from the node to the tips of the tree, i.e., with the ancientness of the node (Pearson r = 0.75, p<0.001, Figure S6). Confirming this interpretation, a comparison of genomes separated by a lapse of time equivalent to the distance between the extant genomes and the ancestor, e.g., strains APEC O1 and 55989, shows a number of distinct genes close to the 1000-gene difference observed at the inferred ancestral genome. When accounting for *E. coli*'s speciation process from the other *Escherichia* spp. it should thus be borne in mind that genes involved in speciation may have disappeared altogether from extant lineages.

To analyse in detail the gains and losses of genes we considered that genes were present at an ancestral node if the probability of presence was higher than 50%, and otherwise were absent. (Variations around this value had little effect of the overall results.) Genes were then classified in 4 mutually exclusive categories: core genome, clade-unspecific (i.e., also present in some genomes not descending from the focal node), clade-specific and present in all descendents from the focal node, or clade-specific but present in only some of the descendents (Figure 5). Most non-core genes are clade-unspecific, especially in nodes close to the root. This is best understood by revisiting Figure 2, which shows that most non-core genes are present in very few genomes. As a result, few genes in the internal nodes are clade-specific and present in all genomes of the clade. The last common ancestor is an exception because it contains many genes present in some *E. coli* genomes but absent in *E. fergusonii*. Elsewhere, very few genes are clade-specific, consistent with the idea that most transferred genes quickly disappear from the populations.

Very recent acquisitions are highly enriched in phage-related genes, except in the branches leading to *Shigella* where transposable elements dominate (Figure 7). Few terminal branches show significant amounts of acquisition of known function genes. The exceptions, UMN026 and IAI39, correspond to the largest terminal branches, which include very ancient and very recent acquisitions. This pattern is suggestive of rare acquisition of genes of known function followed by lower probability of loss for these genes. Stated otherwise, the acquisition of known-function genes is rare, but these genes have a higher probability of being adaptive and, thus, are less likely to be lost. At the opposite extreme, transposable elements and prophage-related genes have high probabilities of being acquired, but since they often have deleterious consequences, they are quickly purged from the populations. As a result, gains inferred in ancestral nodes, i.e., those for which we can still infer an acquisition regarding extant genomes, are enriched in adaptive genes and impoverished in transposable and phage elements.

The pan-genome includes the ancestral genome, which in turn includes the core genome. As one goes from the smallest to the largest gene set one expects to find more accessory and fewer essential functions. Indeed, functions encountered more frequently in the smaller sets include biosynthesis of amino acids, nucleotides, co-factors and proteins, and, to a lesser extent, metabolism of DNA, fatty acids, and phospholipids, transcription and protein fate (Table S5). On the other hand, regulators, cell envelope, biological processes and mobile elements are over-represented in the larger sets. Interestingly, the inferred ancestor of all *E. coli* lacks none of the 23 high-confidence essential genes that are missing in the core genome. It thus provides a better representation of the housekeeping and essential functions of the *E. coli* cell than does the core genome.

### The Role of Genome Repertoire Dynamics in the Commensalism/Pathogenicity Duality

Gene acquisition and loss have important roles in transitions between commensalism and pathogenicity [51],[52]. Epistatic interactions between virulence determinants and the genetic background may also be important [22]. Indeed, the strains with the highest pathogenicity and classified as biosafety level 3 (*S. dysenteriae* serotype 1 and enterohemorragic *E. coli* O157:H7) (Table 1) are closely related (Figure 4). This high degree of pathogenicity is due to toxins that could require a specific genetic background to achieve appropriate expression.

To understand the link between virulence and genetic background, we first looked for functional genes categorically present (i.e., ubiquitous in the clade but absent elsewhere) or absent (i.e., absent in the clade but ubiquitously present elsewhere) within three main phylogenetic groups: A, B1 and B2 (with group D being unsuitable for the analysis as it is paraphyletic) (Table 4 and Table S6). Since only one group A strain was available (*E. coli* K-12 MG1655), we added to this analysis the genome of strain HS (http://msc.jcvi.org/e_coli_and_shigella/escherichia_coli_hs/index.shtml), a group A human commensal strain. Few genes (5 to 81 per phylogenetic group, depending on the group) were found to be specific to and ubiquitous within the particular phylogenetic group, in agreement with the high gene flow observed in the species. However, the numbe of specific genes was higher within group B2 than within other phylogenetic groups, despite the greater number of studied B2 genomes and the greater time of divergence of this phylogenetic group (two factors that should decrease the number of shared genes) (Table 4). This could indicate that these genes stably gained or lost, contribute to the fitness of the group B2 strains. Indeed, only one of these genes corresponds to a transposase and none to phages, whereas 75% have an assigned function. This is significantly higher (Chi square test, p<0.001) than the proportion of genes with assigned functions in the B2 pan-genome (4097 of 8439, 48.5%). Furthermore, the distribution of the genes with assigned functions among different functional categories (‘Product type’ annotations, Table 4) is significantly different for the specific genes as compared with the pan-genome (Chi square test, p = 0.049). The study of Pearson residuals shows that the enzymes and transporters and carriers categories contribute significantly to this difference. Integrative analysis of the documented functions of the specific genes shows a large part of them to be involved in metabolism (Table 5). These observations represent a hallmark of selection and suggest an important role for metabolism in the niche adaptation of group B2 strains that needs to be further substantiated by experimental analyses.

**Table 4:** General characteristics of functional genes specifically present or absent in strains belonging to the main phylogenetic groups or exhibiting a specific phenotype.

Phylogenetic group or phenotype	Specific gene	Total	Assigned function^a^						Phage origin	IS	Unknown function
			Total	Enzymes	Regulators	Transporters and carriers	Factors	Membrane components and structures			
A^b^	Present	19	4	1	1	0	1	1	2	1	12
	Absent	19	4	3	0	1	0	0	10	0	5
B1	Present	23	6	1	0	1	1	3	0	1	16
	Absent	5	4	2	0	1	0	1	0	0	1
B2	Present	62 (36)^c^	39	18	4	10	2	5	0	1	22
	Absent	81 (80)	68	22	12	22	3	9	0	0	13
ExPEC pathotype	Present	16 (13)	14	2	4	0	3	5	0	0	2
	Absent	1 (0)	1	0	0	1	0	0	0	0	0
B2 mouse killer	Present	31 (23)	11	6	3	1	1	0	1	1	18
	Absent	9 (0)	8	6	1	1	0	0	0	1	0
Invasive diarrhoea (*Shigella*)	Present	8+30^d^	0	0	0	0	0	0	3	0	5
	Absent	32	28	12	2	13	1	0	0	0	4

a Functions were assigned according to *E. coli* K-12 MG1655 orthologous gene annotations [96] if any, or to similarity results obtained using the MicroScope analysis pipeline described in [95]. The categories ‘Enzymes’, ‘Regulators’, ‘Transporters and carriers’ (‘carriers’ includes specialized electron-carrying proteins and electron-carrying subunits of enzymes), ‘Factors’ (such as transcription and translation factors, and chaperones), and ‘Membrane components and structures’ are from GenProtEC [125] ‘Product type’ annotations (i.e., types of molecular functions) (Supplementary Table 6). The number of genes in each category does not take into account genes from phage and insertion sequence (IS) origin.

b Considering the complete genome of the strain HS (phylogenetic group A). (http://msc.jcvi.org/e_coli_and_shigella/escherichia_coli_hs/index.shtml).

c Considering the complete genome of enteropathogenic strain E2348/69 (phylogenetic group B2) (http://www.sanger.ac.uk/Projects/Escherichia_Shigella/). This strain is not virulent (0 of 10 mice killed) in the mouse model of extraintestinal virulence [32].

d Genes on the virulence plasmid [56], not detailed in the subsequent columns.

**Table 5:** Integrative analysis of cellular activities specifically present or absent in the group B2 strains.

Genes	Encoded function		Cellular activity
**Present**			
*ptsG*	Subunit of glucose-specific PTS permease	§^a^	Carbohydrate transport
*sucABCD* like	Subunits of 2-keto-glutarate dehydrogenase complex	§	TCA cycle
**Absent**			
*cynRTSX*	Cyanate degradation	§	Xenobiotic degradation
*arsRB*	Arsenate catabolism	§	Xenobiotic degradation
*puuPADRCBE*	Putrescine degradation II	§	Polyamine degradation
*abgAR*	p-Aminobenzoyl-glutamate degradation	§	Aromatic compound degradation
*ddpFDCBAX*	D-Ala-D-Ala degradation	§	Dipeptide degradation
*hcaREFCB*	3-Phenylpropionate degradation	§	Aromatic compound degradation
*melB*	Melibiose permease	§	Carbohydrate degradation
*argKygfGH*	Succinate degradation	§	Dicarboxylic acid degradation
*codA*	Cytosine deaminase	§	Pyrimidine nucleotide biosynthesis
*lsrBFG*	AI-2 transport	§	ATP-dependent transport
*glvC*	Arbutin specific PTS permease	§	Aromatic compound transport
*hyfABCDEFGHIJ*	Subunits of hydrogenase 4	§	Anaerobic respiration
*sfmACDHFfimZ*	Exportation of fimbrial-like adhesin protein	§	Pilus biosynthesis
*lhr*	Putative ATP-dependent helicase	§	DNA replication
*yeaTUVWX*	Hydroxybutanedioic acid degradation	§	Dicarboxylic acid degradation
*yggF*	Putative hexose phosphate phosphatase		Carbohydrate degradation

a § indicates that the genes are present or absent when the enteropathogenic strain E2348/69 (phylogenetic group B2) (http://www.sanger.ac.uk/Projects/Escherichia_Shigella/) is included.

We then examined whether the presence of specific genes could be related to a specific phenotype. No gene was specific either to commensal strains or to pathogenic strains in general. However, in extraintestinal pathogenic strains (ExPEC pathotype) 16 genes were specifically present and 1 was specifically absent (Table 4). Most of these genes have an assigned function corresponding mainly to 2 clusters: (i) the *pap* operon, a well-known adhesin determinant involved in the pathogenesis of urinary tract infection [53], and (ii) two genes coding for an aldo-keto reductase activity (one of these genes shares 95% identity with *akr5f1* gene from *Klebsiella* spp [54]) and a divergent *lysR* family regulatory gene (Table S6). In addition, when considering intrinsic extraintestinal virulence potential as assessed using a mouse model of septicaemia that avoids host variability [32], no gene specific to the virulent phenotype was identified. All these data indicate that extraintestinal virulence is a multigenic process resulting from numerous gene combinations and multiple redundancies. Furthermore, the fact that no gene specific to extraintestinal infection could be identified reinforces the hypothesis that extraintestinal virulence is a coincidental by-product of commensalism [42]. This suggests that the development of vaccines specific for extraintestinal infections will be extremely difficult. Any gene target likely will also be present in some commensal strains; therefore, such vaccines will presumably lead to potentially undesirable modification of the resident microbiota. Twenty and 4 genes were specifically present and absent, respectively, in intestinal pathogenic strains (with *Shigella* excluded from the analysis). All except 2 of these genes are of phage and IS origin or of unknown function.

We also took the unique opportunity to do a comparative genomic analysis of the recently reported B2 human commensal clone (represented by strain ED1a, as sequenced in this work), which is avirulent in the mouse lethality model [55]. Thirty-one genes were specifically present and 9 were specifically absent in the B2 strains that were virulent in the mouse lethality model (B2 mouse killer strains) (Table 4 and Table S6). Interestingly, among the 9 absent genes, 8 belong to the *mhp* operon. The catabolic pathway of phenylpropionate and its derivatives is split in *E. coli* into two operons, the *mhpR mhpABCDFET* and the *hcaR hcaEFCBD* operons. The *hca* operon is specifically absent in all the group B2 strains (Table 5). Strain ED1a is thus an exception, as it possesses the *mhp*, but not the *hca* operon. This may suggest some sort of involvement of aromatic compounds in the virulence of B2 strains.

A similar comparative genomic analysis involving the *Shigella* strains identified 38 genes (30 from the virulence plasmid [56], as expected) to be specifically present, but also 32 genes to be specifically absent (Table 4). Excluding the plasmid genes, 70% have an assigned function, which is significantly greater (Chi square test, p<0.001) than for the genes of the *Shigella* pan-genome (3832 of 9351, 41%). Here again, the distribution of the genes with assigned functions among different categories (Table 4) is significantly different from the *Shigella* pan-genome (Chi square test, p = 0.027), with a disproportionate emphasis on the transporters and carriers category, and more generally on metabolism-related functions (Table 6). The specificity of this pattern of gene loss suggests a footprint of selection through an antagonistic pleiotropy mechanism of adaptation [57] during the very peculiar *Shigella* intracellular life style. Such a life style also leads to the reduced effective population size of *Shigella*, and to less efficient selection [49]. Thus, it has been argued frequently that gene loss in *Shigella* is the result of independent mutation accumulation. It is likely that most gene loss in *Shigella* is indeed the result of less efficient selection, but our data suggest that inactivation of these 32 genes, or a fraction of them, is positively selected.

**Table 6:** Integrative analysis of cellular activities specifically absent in *Shigella* strains.

Genes	Encoded function	Cellular activity
*prpBCDER*	Methylcitrate cycle	Carboxylate degradation
*codBA*	Cytosine degradation	Pyrimidine salvage pathway
*lacY*	Lactose permease	Carbohydrate degradation
*allD*	Allantoin degradation IV	Amine degradation
*fiu*	Outer membrane receptor for iron-regulated colicin and the siderophore dihydroxybenzoylserine	Outer membrane transport
*speG*	Spermidine biosynthesis	Polyamine biosynthesis
*guaDygfOQ*	Guanine salvage	Purine degradation
*agaSkbaYagaB*	N-acetylgalactosamine (or galactitol) degradation	Carbohydrate degradation
*aapQ*	ABC transporter of polar amino acids	Amino-acid transport
*rbsB*	Ribose ABC transporter	Carbohydrate transport
*cadBC*	Decarboxylation of lysine	Polyamine biosynthesis
*ydiF*	Acetate CoA transferase	Fatty acid oxidation
*yaaJ*	Alanine/Glycine transporter	Amino-acid transport

We further substantiated the role of polyamine metabolism and transport in *Shigella* virulence by identifying the absence of (i) *speG* involved in spermidine biosynthesis and (ii) the *cad* genes involved in cadaverine biosynthesis [52]. It has been shown that the presence of cadaverine prevents the escape of *S. flexneri* from the phagolysosome [58]. The absence of spermidine acetylation by SpeG could preclude export of acetyl-spermidine. Another negative phenotype of *Shigella*, not often discussed in relation to pathogenicity, is their lactose-negative character, arrived at by convergent evolution [7]. We found that within the lactose operon region, the only gene always inactivated is *lacY*, the permease coding gene. As the role of pH is essential for colonisation of a novel niche, the lactose permease, a proton-driven transporter, may act against adaptation of the bacteria to the acidic phagolysosome. One might speculate that a beta-galactoside present in the phagolysosome could be transported out with import of protons, leading to a proton influx that would rapidly kill the bacteria. Gene decay would thus have protected *Shigella* against this host protective mechanism.

### Hotspots of Gene Acquisition and Loss Are the Same in Every Genome

Bacterial chromosomes are highly organised with respect to their interaction with cellular processes such as replication, segregation and transcription [59]. To understand how the massive flux of genes we have documented can be compatible with chromosome organisation we inferred the number of insertion and deletion events at each branch of the species tree (see Materials and Methods, Figure 7 and Figure S5). The average acquired fragment contains 4.3 genes, whereas the losses average only 3 genes (Wilcoxon test, p<0.001). These values are nearly half the previously published ones [60], most likely because our analysis includes many more closely related strains and uses the inference of ancestral states, leading to a more accurate estimation of multiple contiguous insertions and deletions. The total number of genes gained and lost is expected to be roughly similar, since enterobacterial genomes have relatively similar sizes. Therefore, gains correspond to larger fragments and losses to more frequent events. The size of the fragments of gains or losses varies widely. More than half of inferred losses and gains involve a single gene. Only 5% of losses and 8% of gains correspond to events including more than 10 genes, but these include around half of the genes involved in gains and losses (54% and 40%, respectively). These values are similar for internal branches, small external branches and long external branches (Kruskal-Wallis test, p>0.05), suggesting that our inference is unbiased with respect to successive events taking place at the same locations in long branches or by selection-purging older events in internal branches. Variation in gene repertoires has been described as being scattered on the chromosome of *E. coli* and balanced between the two replichores [61]. For the numerous small insertions and deletions this distribution results naturally from random insertion/deletion of genetic material. Such small indels are expected to have little impact on the large-scale organisation of the genome.

**Figure 8:**
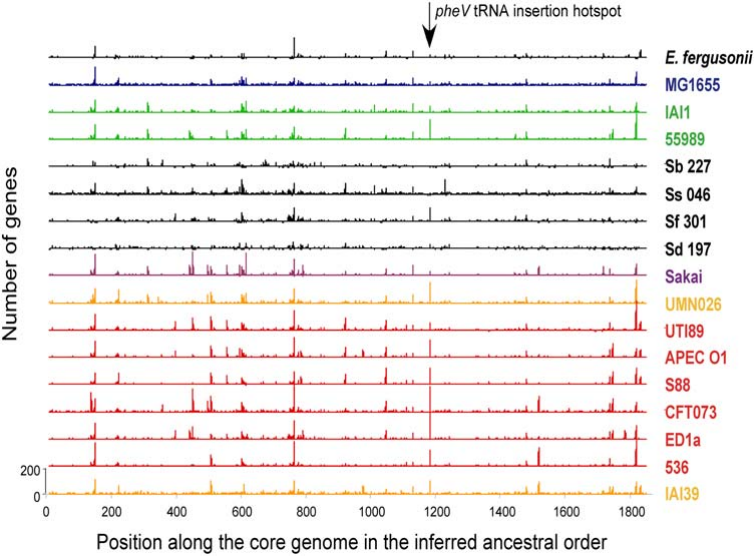
Global view of insertion/deletion hot spots. Number of genes (ranging from 0 to 200) in indels along the genomes of modern strains according to the ancestral gene order of the core genome. The numbers on the x-axis represent the order of genes in the core genome, which has the same order as *E. coli* K-12 MG1655.

What about the very large insertions/deletions? The 554 such events that involve more than 10 genes over-represent insertions over deletions (Fisher exact test, p<0.001), as expected given that insertions are typically larger. These events involve an average of 29 genes each, with a maximum of 157 genes for a single event. Unsurprisingly, known pathogenicity islands and prophages are included in these large events. The insertion of very large DNA segments, even if it takes place in intergenic regions, will have important consequences for the organisation of genomes. Therefore, we investigated where such insertions took place. We used the ancestral order of the core genome and computed, for each genome, the number of non-core genes between consecutive core genes. (The rare positions corresponding to synteny breakpoints in a genome were ignored for that genome.) This analysis revealed that in most genomes gene acquisition and loss takes place at precisely the same locations across genomes, i.e., between the same two contiguous core genome genes (Figure 8, Figure S7). Thus, the *E. coli* genome contains striking integration hotspots.

**Figure 9:**
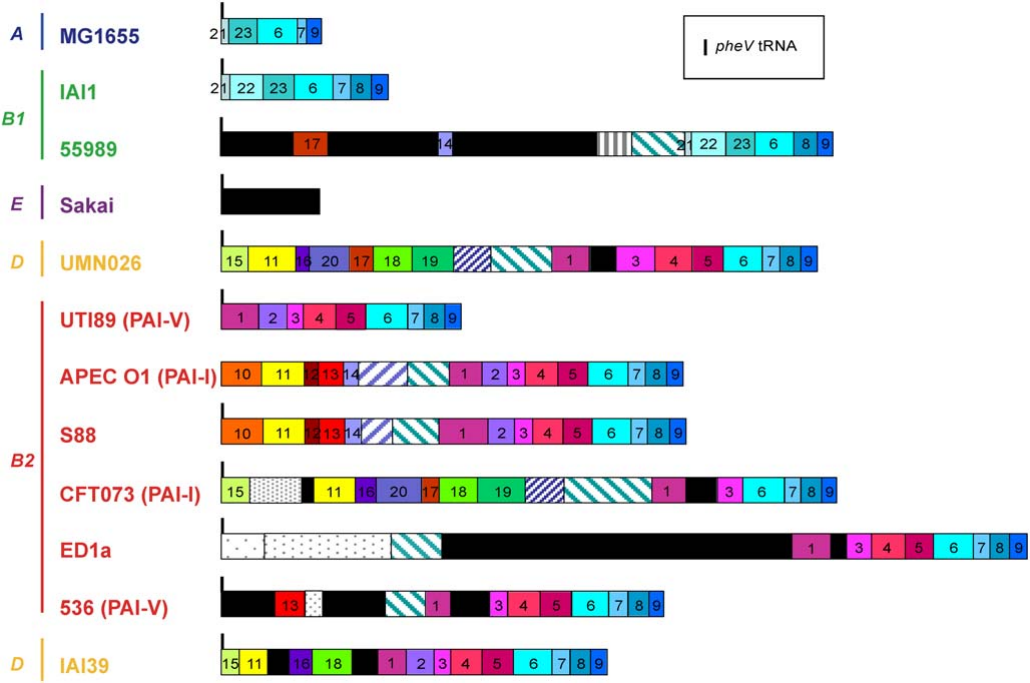
The genomic island at the *pheV* tRNA insertion hot spot in the different *Escherichia coli* strains. The figure provides a synthetic view of the *pheV* tRNA insertion hotspot in the different studied *E. coli* strains. This region has been defined using the synteny breaks among 12 *E. coli* strains. In *E. coli* K-12 MG1655, the genes immediately flanking the *pheV* tRNA gene are the ECK2960 gene (*speC*, ornithine decarboxylase) and the ECK2981 gene (*pitB*, phosphate transporter). In strain APEC O1, the *pheV* tRNA gene is absent. As most *E. coli* genomic regions have a composite structure, e.g., a region partially conserved or found in different synteny groups in other strains (i.e., at different genomic locations), we have manually divided this large genomic island into sub-regions (or modules), which are found in only a subset of the compared *E. coli* strains. Homologous modules have the same colour code and identifying number throughout. A total of 23 homologous modules were defined. The composition of these modules (i.e, the lists and functional descriptions of the constituent genes) is available in Supplementary Table 7. Black modules are strain-specific. Modules with hatched patterns correspond to repeated regions. Modules with grey dotted patterns are found in other strains but at another genomic location. The pathogenicity island published as PAI-V in UTI89 and 536 or PAI-I in APEC O1 and CFT073 ends just before module number 6.

An example of an insertion hotspot at *pheV* tRNA gene in 12 *E. coli* strains is represented in Figure 9. This example shows that very different genetic information occurs at the same hotspot in different genomes. Interestingly, it also shows a patchy structure, with the information segmented into modules that can be found independently in other locations of other genomes. The presence/absence of specific modules is uncorrelated with either the phylogenetic group or the pathotype. For example, module 14 (immunoglobulin-binding genes, which encode a surface-exposed protein that binds immunoglobulins in a nonimmune manner) is present in strains 55989 (group B1, EAEC), APEC O1 and S88 (group B2, ExPEC); module 19 (N-acetylneuraminic acid degradation) is present in strains UMN026 (group D, ExPEC) and CFT073 (group B2, ExPEC) only; and module 2 (N-acetylneuraminic acid synthesis), with the pattern [1-2-3-4-5] is absent in strains UMN026, CFT073, ED1a (group B2, commensal) and 536 (group B2, ExPEC). Actually, the organization of the modules is identical in APEC O1 and S88, and very similar in UMN026 and CFT073. Such a modular structure of the hotpots suggests either multiple integrations or frequent recombination between integrative elements.

While 51% of all intergenic regions between pairs of contiguous core genes show no single insertion or deletion in any of the 21 genomes, we found 133 such locations with an average of more than 5 non-core protein-coding genes per genome. These locations accumulate 71% of all non-core pan-genome genes. Nearly two thirds of the hotspots (62%) lack prophages in all genomes. Genes in hotspots have an average of 4 orthologs in the other genomes. Yet, this average is somewhat misleading since some genes have many orthologs and the majority has practically none. Therefore, hotspots correspond to regions of abundant and parallel insertions and deletions of genetic material. While the existence of large insertions and deletions in *E. coli* has been abundantly described [62],[63], our data shows that these events take place systematically at the same regions in different genomes.

### What Creates Such Hotspots of Gene Acquisition and Loss?

The genomes of *E. coli* harbour many prophages and genomic (e.g., pathogenicity) islands, which typically integrate in the chromosomes by site-specific recombination in a tRNA gene through the action of phage-like integrases [64]. We assessed how frequently such elements are associated with hotspots. We found that 83% of the hotspots showed no tRNA gene at the edge of the element, within a 3-gene window, in any of the genomes. When tRNA genes were indeed found, they tended to be present in practically all genomes. Since each *E. coli* genome has close to 100 tRNA genes, the occurrence of tRNA genes in the neighbourhood of 17% of hotspots can partly be due to chance. We therefore searched the hotspots for homologs of a set of 8067 integrases obtained from Swissprot by using Blastx to include potentially pseudogenised integrases. Using our standard criteria for homology (see Materials and Methods) we found that more than half of the hotspots have no integrase homolog in any genome, whereas fewer than 6% have integrases in the majority of the genomes. Decreasing the similarity criterion for a homolog to 40% identity increases the number of putative integrases, but half of the hotspots still have at most two distant homologs of integrases, and these are present in the majority of genomes in only 17% of the hotspots. This seriously challenges the widely held view that *E. coli* integration hotspots are mostly determined by the distribution of tRNA genes and that such integrations systematically take place by phage-like integrase elements.

What else could create such hotspots? It would be predicted that selection for preserved integrity of composite regulatory elements, genes, operons, supra-operonic structures, nucleoid folding-domains and macrodomains should reduce the number of locations where large insertions can occur without causing significant loss of fitness [59]. For example, ∼90% of the genomes consist of genes and half of the remaining 10% represents intergenic regions within operons. Selection should thus effectively forbid most insertion points in the genome. However, once a permissive region has acquired a large element, and since most transferred DNA has no adaptive value, subsequent integration in the region becomes more likely because the region offers a larger target for neutral insertion. The insertion of a large element in a permissive region will then result in a founder effect that amplifies the likelihood of the permissive region becoming a hotspot.

Some regions may be more prone to recombination because of their sequence/motif composition, e.g., the presence of motifs recognised by integrases or the machinery of homologous recombination. We tested if the regions flanking the hotspots showed higher frequencies of chi sequences, but found no significant effect. DNA structure may also play a role, e.g., because chromosome folding leaves some regions more exposed than others for recombination with incoming DNA [65]. The 133 hotspots contain 61% of all synteny breakpoints, which is much more than expected given the number of these locations (Chi square test, p<0.0001), but close to the expected value if one considers that rearrangements cannot disrupt core genes and that the hotspots are very large (Chi square test, p>0.05). This shows that insertion/deletion hotspots are also rearrangement hotspots, even though we initially removed rearranged positions to identify the insertion/deletion hotspots (thus being conservative). It also suggests that rearrangements occur in these regions because they are permissive to change not because they are intrinsically recombinogenic, since the frequency with which they rearrange simply reflects their larger size. However, even if hotspots are not intrinsically recombinogenic they can still be caused by the brokering effect of homologous recombination. Indeed, incoming DNA once integrated in one genome can propagate within the population by lateral transfer via classical homologous recombination involving the homologous flanking regions. Given the observed rates of recombination in the species, this mechanism could quickly lead to the horizontal spread of highly adaptive newly acquired genes. We describe some evidence for this in the next section.

### Hotspots of Phylogenetic Incongruence

For any given sequence alignment, the likelihood of the overall gene tree topology, i.e., the phylogenetic congruence, reflects the extent to which the phylogenetic signal of the sequences was altered by recombination. While the concatenate of genes provides a strong phylogenetic signal, the individual genes' histories can be very diverse as a result of recombination. Furthermore, these histories may depend on the genes' positioning in the chromosome. Notably, if homologous recombination helps in disseminating recent acquisitions, as we propose, the core genome around these hotspots should show signs of recombination as indicated by phylogenetic incongruence. We therefore made an analysis in 5 kbp sliding windows along the multiple genome alignment to identify the most phylogenetically incongruent regions (see Material and Methods). This method identified two large regions of very strong incongruence, one centred around *rfb* (Figure S8), the operon involved in O antigen synthesis, and the other around the *leuX* tRNA gene, and including *fimA*, which is under diversifying selection and is involved in the adhesion of bacteria to host cells [66]. Both loci were previously identified as hotspots of phylogenetic incongruence [67],[68]; the present analysis reveals how much they affect the chromosome.

Recombination at the *rfb* locus significantly affects congruence within a striking 150 kbp surrounding region, i.e., from positions 1988 kbp to 2138 kbp (100% of windows tested had scores lower than 1.96 standard deviation away from the average, with an average of −4.84 and peaks at −10.19). The *fim* locus includes an incongruence region close to 200 kbp in length (from positions 4421 kbp to 4618 kbp, average −2.54 standard deviation and 73% with lower than −1.96 standard deviation and peaks at −6.65). Interestingly those two regions are centered on integration hotspots and encompass 11 of the 133 hotspots of integration. The genes present in such loci arose most likely by lateral transfer since they are highly dissimilar between strains. For example, genes at the *rfb* locus genes can exhibit less than 50% similarity, while the *leuX* locus encompasses a highly variable assortment of non-homologous inserts in all the genomes sequenced. Hence at least for those two major loci we find a striking link between hotspots of integration and hotspots of homologous recombination. In the case of the *rfb* locus, it is worth noting that the incongruence signal we observe might be a composite signal, due not only to *rfb* but also to neighbouring loci. Within the above defined *rfb* region of incongruence, a flagella locus (*fli* operon) associated with two hotspots of integration is also under diversifying selection. Moreover, the high pathogenicity island (HPI) is integrated within that high recombination region in many isolates and corresponds also to a hotspot of integration. It has been suggested that after a recent and unique integration event, the HPI has propagated within the species by homologous recombination [69]. The propagation or diversification of these loci, located to the left of *rfb*, through homologous recombination might generate the asymmetrical pattern of phylogenetic incongruence we observe around the rfb locus (extended incongruence on the left side of the *rfb* locus) (Figure S8).

We found 23 other regions with weaker signatures of incongruence (i.e., with a 5 kbp sequence incongruence score more than 2 standard deviations from the average), each spanning less than 20 kbp. It is important to note that most of these incongruent regions include genes involved in diversification of genetic information and often pathogenicity. The vast majority of these include 3 groups of common genes. First are regions with the porin-encoding genes *ompA* and *ompC*, the flagella-encoding genes, the *rfa* locus coding for the core lipopolysaccaride and genes coding for several membrane proteins such as LolCDE, CcmABCDE, ABC transporter, AroP APC transporter, LplT-aas, FadK, YeaY, EamB, YhgE and YicG membrane proteins. These loci are probably involved in diversifying selection since they code for antigenic proteins exposed at the cell surface. Second are two regions encompassing mismatch repair genes (*mutS* and *mutH*) that have been shown to be under selection for cycles of inactivation and reacquisition through recombination [70]. Third is a region associated with the integration of a locus that can provide resistance to phages through clustered regulatory interspaced short palindromic repeats (CRISPRs) [71].

All available methods estimate the effective, not the intrinsic, recombination rate. Effective recombination results from the intrinsic recombination rate and the ensuing selection on recombinants. Most of the phylogenetic incongruence hotspots we found contain genes under diversifying selection, for instance to escape immune pressure or to acquire resistance to phage. Hence, it is very likely that differences in the intensity of selection might be responsible for the differences observed in the size of the regions affected by a phylogenetic incongruence hotspot. A recombinant carrying a new allele at a locus under strong diversifying selection will be selected and thus will increase rapidly in frequency in populations. Hence, the recombinant will invade the local population before any further recombination occurs at the locus [72]. In that case, sampling the genome after the action of natural selection allows identification of the original recombining fragment. In contrast, if selection is moderate, the recombining fragment that brought the interesting allele into the genome will be covered by many further recombination events before it reaches high frequency. In this case, only fragments around the selected allele will retain the trace of the recombination event. As a consequence, when selection is intense, one expects to identify long recombinant fragments in some strains, as we did at the *rfb* or *leuX* loci. Our observations suggest that the intensity of diversifying selection acting on the *rfb* and *leuX*-*fimH* loci are under very strong selective pressure compared with the diversifying selection acting on the core LPS, the flagella or some of the porins. The fact that most hotspots of integration (117 among 133) do not result in hotspots of phylogenetic incongruence suggests that they carry neutral or deleterious genes. Conversely, it also suggests that some horizontally acquired genes can be highly beneficial (e.g., 11 hotspots of phylogenetic incongruence around the *rfb* or *leuX*-*fimH* locus) or moderately beneficial (e.g., 4 hotspots of integration associated with hotspots of phylogenetic incongruence) and that this results in different selection footprints in the neighbouring core genome.

### Recombination and Chromosome Organisation

**Figure 10:**
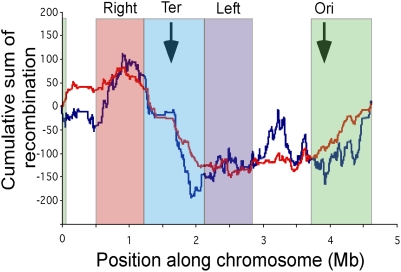
Standardized cumulative sum of effective gene conversion rate and G+C content. Gene conversion rate (i.e., probability of being involved in a gene conversion event C_gc_.L_gc_) is shown in blue, and G+C content in red. A decrease in the cumulative sum reflects regions of lower-than-expected values of the statistics. Around the terminus domain, we found a decrease in both recombination and G+C content. Coloured boxes represent the 4 different organisation macrodomains (Right, Ter, Left, Ori). The arrows point towards the origin and terminus of replication.

The existence of integration and phylogenetic incongruence hotspots brings to the fore the conflict between genome dynamics and organisation. We therefore analysed the variation in recombination along the backbone sequence (as estimated by a population genetic-based approach), using a sliding window of 3 kbp on the multiple genome alignment and a step size of 500 bp. This analysis revealed a large region around the terminus of replication with a particularly low ratio between gene conversion and mutation rates (C_gc_/theta) (Figure 10). The region between 1 Mb and 2 Mb shows lower gene conversion rates, since there is 20% lower chance for a base to be involved in a gene conversion event (C_gc_×L_gc_, unilateral t test: p = 1e-21). This region also shows 10% lower levels of polymorphism (theta of Watterson, p = 1e-7), i.e., variations within the *E. coli* species, and 2% lower G+C content (Figure 10). A+T richness at the terminus region has been suggested to result from higher mutation rates [73]. Based on comparative genomics with *Salmonella*, it was also shown that divergence, i.e., the genetic distance between species, slightly increased closer to the terminus [74],[75], further supporting the hypothesis of a higher local mutation rate. Using our newly sequenced outgroup genome *E. fergusonii*, which unlike *Salmonella* does not shows saturation of synonymous substitutions, we found that the terminus domain has synonymous and non-synonymous substitution rates twice as high as the rest of the chromosome. While decreased G+C content and increased divergence could reflect a higher mutation rate at the terminus, such an interpretation is contradicted by the observed lower polymorphism.

Theoretical population genetic studies have shown that the fluctuation of recombination frequency along chromosomes affects the level of polymorphism and the efficiency of selection [76]. When there are numerous deleterious mutations and low recombination rates, a fraction of the population bearing deleterious alleles is doomed to disappear in the long term without contributing to the gene pool of the future population. The relevance of this phenomenon, referred to as background selection, requires the existence of deleterious mutations of moderate effects, i.e., mutations that can persist for some time in the population before selection wipes them out. At the population level, this result in an excess of rare alleles, which can be estimated by Tajima's D statistics. We found that overall a gene's average Tajima's D was slightly negative (indicating an excess of rare alleles). However, the Tajima's D of synonymous mutations was null, while that of non-synonymous mutations was much more negative (Figure S9). This suggests that most non-synonymous mutations are deleterious since, in contrast to synonymous mutations, they do not increase in frequency within the population reflecting the purging effect of natural selection. Therefore the conditions for the action of background selection are met. Furthermore, under background selection, a reduced recombination rate results in a decreased polymorphism (such as we observed around the terminus), an increased fraction of rare alleles and a decreased efficiency of selection [76]. The terminus region shows a lower Tajima's D than the rest of the chromosome (Student bilateral test, p<0.00001). It also shows a reduced ratio of non-synonymous to synonymous polymorphism (Student bilateral test, p<0.002). This suggests that more non-synonymous mutations, presumably slightly deleterious, persist around the terminus. When applying the same approach to the ratio of non-synonymous to synonymous divergence, we found more non-synonymous mutations fixed around the terminus (Student bilateral test, p<0.05). All these observations are in agreement with a reduced efficiency of selection at this region, compatible with the effects of background selection in low recombination regions.

The observed co-occurrence of lower GC% and lower recombination rate at the terminus could also indicate a reduced action of recombination to purge deleterious mutations in that region. Most mutations tend to be from GC to AT and, as our analysis of Tajima's D revealed, most non-synonymous mutations are presumably deleterious. Consequently, if a segment of DNA lacking deleterious mutations replaces a fragment that contains many of them, presumably GC-towards-AT, the resulting recombinant will be selected for and, hence, will increase the GC content. Therefore, in regions of low recombination rate, a greater number of deleterious GC-towards-AT mutations will accumulate. This is in agreement with recent analyses showing an association between G+C enrichment and purifying selection of non-synonymous substitutions [77].

Alternatively, recombination could have a direct mutagenic effect. The biased gene conversion hypothesis, which enjoys growing popularity to explain the G+C heterogeneity in mammalian genomes, states that mismatches in recombination heteroduplexes are repaired in favour of G and C [78]. If in *E. coli*, as in humans and elephants, biased gene conversion results in G+C enrichment, then lower conversion rates at the terminus should result in the observed lower G+C content. Biased gene conversion results in the biased segregation of nucleotides and, therefore, in a gap between the composition of genomes and their mutation patterns. We had previously found that such a gap was common in bacterial genomes [79]. The re-assessment of those data showed that in all 6 *E. coli* genomes considered in our previous work the G+C content was higher than expected given the observed mutational patterns. This suggests that mutations towards G and C are more likely to attain fixation, in agreement with the hypothesis of biased gene conversion in *E. coli*.

Both hypotheses are compatible with the pattern observed, but attribute different meaning to reduced GC% at the terminus. In the biased gene conversion hypothesis, lower GC% is just a result of the mutational bias induced directly by recombination, while in the second one a lower GC% reflects the lower efficiency of recombination to purge slightly deleterious mutations and is therefore a signature of maladaptation.

Why should conversion rates be lower at the terminus? This could be explained by the patterns of genome organisation. Firstly, in exponentially growing *E. coli* cells the regions near the origin of replication are present in many more copies than the regions near the terminus [80]. Therefore, they provide more abundant targets for gene conversion with foreign DNA. Because of gene dosage effects the origin of replication is also enriched in highly expressed genes, which are under stronger purifying selection. This might lead to lower observed rates of mutation or to higher rates of recombination, if recombination's role is to maintain housekeeping functions [81]. Secondly, the low recombination / high A+T content region near the terminus coincides with the boundaries of the Ter macrodomain of chromosome folding in *E. coli* [82]. Four macrodomains (Ori, Ter and two flanking Ter named Right and Left: Figure 10) have been described [82]. These macrodomains are compacted structures that act as intra-chromosomal recombination insulators. Tight compaction of the Ter domain might lead to lower conversion rates with incoming DNA. The link between the frequency of gene conversion, biased sequence composition, chromosome compaction and selection highlights the intimate association between genome dynamics and chromosome organisation.

### Concluding Remarks

New high-throughput sequencing technologies will soon allow the sequencing of hundreds of strains of the same species, but not to completion and closure. The genomes of *Escherichia* that we sequenced, the previously sequenced ones, plus others and our re-annotation efforts, will provide a solid basis for the next phase of *E. coli* genomics in which population genetics and experimental evolution will have important roles. We also hope to have contributed to narrowing the gap between population genetic and phylogenetic approaches in studying genome evolution by showing that they both can be used to untangle the effects of gene dynamics on adaptation and genome organisation. Within a bacterial species, the core genome evolves mostly through mutation and recombination, whereas the rest of the genome is also subject to horizontal gene transfer. While this fits with qualitative observations in other species [83]–[85], in *E. coli* the rates of lateral transfer are particularly high and lead to very short gene residence times. Furthermore, once introduced by lateral transfer, genes can spread by homologous recombination at the flanking regions. Despite this very high gene flow, genes co-exist in organised genomes. The conflict between genome dynamics and organisation may have resulted in the striking integration hotspots, which confine regions of high instability. It may also have resulted in regionalised gene conversion.

Chromosomal plasticity certainly accelerates the adaptation of *E. coli* to varied environments. First, it allows many parallel and specific evolutionary pathways of gain and loss of genes leading to convergent phenotypes. Second, it allows multiple gene combinations that, with epistatic interactions, will result in phenotypic diversification. As a result of these complex evolutionary patterns, most often there is no simple association between the presence of a gene and a given phenotype. For example, our genomic analysis of the extraintestinal virulence phenotype suggests that it will be very difficult to develop a vaccine against extraintestinal infections without affecting also resident intestinal microbiota because there is no single determinant of the former. The vast diversity among *E. coli* genomes suggests that the key to understanding the emergence of such phenotypes resides in ampler sampling of natural isolates combined with a systematic analysis of the data at a physiological level.

## Materials and Methods

### Bacterial Strains

Six *E. coli* strains as well as the type strain (ATCC 35469^T^) of *E. fergusonii*, the closest *E. coli*-related species [31], were selected for complete genome sequencing (Table 1). Among the *E. coli* strains, 2 were commensal: IAI1 (serogroup O8) was isolated from the faeces of a young healthy military conscript in the 1980s in France [23] and ED1a (serogroup O81) was isolated in the 2000s from the faeces of a healthy man in France and belongs to a human-specific widespread commensal clone that is increasing in frequency [55]. Four *E. coli* strains were pathogenic. Enteroaggregative *E. coli* strain 55989 was originally isolated from the diarrheagenic stools of an HIV-positive adult suffering from persistent watery diarrhea in Central African Republic [86]. The enteroaggragative pathotype is recognized as an emerging cause of diarrhoea in children and adults worldwide [87]. Among the three extraintestinal pathogenic strains, IAI 39 (serotype O7:K1) was isolated from the urine of a patient with pyelonephritis in the 1980s in France [23]. UMN026 (serotype O17:K52:H18) was isolated from a woman with uncomplicated acute cystitis in 1999 in the USA (Minnesota) and is a representative of a recently emerged *E. coli* clonal group (“clonal group A”) that is now widely disseminated and a cause of drug-resistant urinary tract and other extraintestinal infections [88]. S88 (serotype O45:K1:H7) was isolated in 1999 from the cerebro-spinal fluid of a new born with late-onset neonatal meningitis in France and represents what is now considered a highly virulent emerging clone in France [89]. These strains were distributed in 3 of the 4 main *E. coli* phylogenetic groups: IAI1 and 55989 belong to group B1, UMN026 and IAI392 belong to each of the two major subgroups within group D, and ED1a and S88 belong to subgroups VIII and IX, respectively, within group B2 [42]. Few data are available on *E. fergusonii* strains. They have been isolated from humans and warm blood animals, sometimes in pathogenic (intestinal and extraintestinal) conditions [90]–[92]. The main characteristics of the 14 strains (8 *E. coli sensu strictu* and 6 *Shigella*) with freely available genomes at the time of the study are presented in Table 1. These genomes were used for comparison purpose.

### Sequencing

Three DNA libraries were constructed to determine, for each strain, the complete genome sequence. Two of the libraries were obtained after mechanical shearing of the genomic DNA and cloning of the resulting 3 kbp and 10 kbp inserts into plasmids pcDNA2.1 (Invitrogen) and pCNS (pSU18 derived), respectively. DNA fragments of about 30 kbp generated after partial digestion using *Hin*dIII and/or *Sau*3A were introduced into pBeloBac11. Vector DNAs were purified and end-sequenced using dye-terminator chemistries on ABI3730 sequencers to provide an average of 12-fold coverage for each genome. A pre-assembly was made without repeat sequences, as previously described [93] using Phred/Phrap/Consed software package (www.phrap.com). The finishing step was achieved by primer walking, transposition and PCR.

### Annotation and Re-Annotation of the *Escherichia* Genomes

Once the consensus sequence of a first complete (single contig) assembly was available for one of the new genomes, gene prediction was conducted using the AMIGene software [94]. The predicted coding sequences (CDSs) were assigned a unique identifier prefixed with “ECED1_” for *E. coli* ED1a, “EC55989_”, for E. coli 55989, “ECIAI1_” for E. coli IAI1, “ECIAI39_” for *E. coli* IAI39, “ECS88_” for E. coli S88, “ECUMN_” for E. coli UMN026, and “EFER_” for *E. fergusonii* ATCC. These identifiers start with ‘p’ if the corresponding CDSs are encoded on plasmids. The sets of predicted genes were submitted to automatic functional annotation, as previously described [95]. Apart from the plasmid-encoded genes, the final functional assignation was based on the transfer of the recently updated *E. coli* K-12 MG1655 annotations [96] between strong orthologs i.e., 85% identity over at least 80% of the length of the smallest protein (Table S2A). Sequence data for comparative analyses were obtained from the NCBI database (RefSeq section, http://www.ncbi.nlm.nih.gov/RefSeq). Putative orthologs and synteny groups (i.e., conservation of the chromosomal co-localisation between pairs of orthologous genes from different genomes) were computed between each newly sequenced genomes and all the other complete genomes, as previously described [95]. All these data (syntactic and functional annotations, results of comparative analysis) are stored in a relational database, called ColiScope. Manual validation of the automatic annotation by multiple users in different locations was performed using the MaGe (Magnifying Genomes, http://www.genoscope.cns.fr) web-based interface. For each newly sequenced genome, only ‘specific’ regions, i.e., those containing genes not orthologous to ones in *E. coli* K-12 MG1655 or to expert annotated genes in another genome of the ColiScope project, were manually annotated (Table S2A). In total, 9776 genes were annotated by our group.

This expert work was also used to re-annotate the other public and *Shigella* genomes. This allowed the creation of a set of consistent expert annotations for the 20 genomes. First, we integrated these genomes into the ColiScope database by using MICheck, a method that enables rapid verification of sets of annotated genes and frameshifts in previously published bacterial genomes [97]. Some inaccurate or missed gene annotations were defined for these genomes (see Table S2B and Table S3 for the list of newly predicted genes in the 14 analyzed genomes). Second, we automatically transferred the functional annotation of *E. coli* K-12 MG1655 genes, or genes annotated in the context of this project to the genes in the other genomes that showed very strong sequence similarity (85% identity over at least 80% of the length of the smallest protein). The remaining genes, i.e., those without orthologs in *E. coli* K-12 MG1655 or one of the new *Escherichia* genomes, retained the original functional annotations (column ‘Specific’ genes in Table S2B).

The new *E. coli* and *E. fergusonii* nucleotide sequences and annotations data have been deposited in the EMBL database (http://www.ebi.ac.uk/embl; see accession numbers list below). In addition, the ColiScope database, which includes all data for the set of *Escherichia* and *Shigella* strains sequenced to date, is publicly available via the MaGe interface at https://www.genoscope.cns.fr/agc/mage.

### Assignment of Orthology

A preliminary set of orthologs was defined by identifying unique pairwise reciprocal best hits, with at least 80% similarity (∼85% identity) in amino acid sequence and less than 20% difference in protein length. The analysis of orthology was made for every pair of *E. coli/Shigella* genomes. The core genome, consisting of genes ubiquitously found among all strains of the species, was defined as the intersection of pairwise lists.

For every pair of genomes this list of persistent orthologs was then supplemented, with attention to conservation of gene order. Because (i) few rearrangements are observed at these short evolutionary distances, and (ii) horizontal gene transfer is frequent, genes outside conserved blocks of synteny are likely to be xenologs or paralogs. Hence, we combined the homology analysis (protein sequence similarity ≥80%, ≤20% difference in protein length) with the classification of these genes as either syntenic or nonsyntenic, for positional orthology determination. The analysis was made for every pair of *E. coli/Shigella* genomes. The definitive list of orthologs of the pan-genome was then defined as the union of pairwise lists.

A syntenic block was defined as a set of consecutive pairs of genes in the core genome. Conserved order gene blocks are obtained by comparison of the localisation of best bi-directional hit pairs in the core genome, adopting a window size of one gap.

These lists were also used to perform gene accumulation curves using R, which describe the number of new genes and genes in common, with the addition of new comparative genomes (Figure 1). The procedure was repeated 1000 times by randomly modifying genome insertion order to obtain median and quartiles.

### Assignment of Homology and Orthology

In the same bacterial species, homologs (paralogs, orthologs, xenologs) were defined by identifying reciprocal blastp, with ≥80% similarity in amino acid sequence and ≤20% difference in protein length. Among different proteobacterial species, orthologs were defined by identifying unique pairwise reciprocal best hits, with ≥40% similarity in amino acid sequence and ≤20% difference in protein length. The analysis of orthology was made with 99 proteobacterial genomes.

### Whole Genome Multi-Alignments of the 20 *E. coli* Strains

Whole genome alignments of the 20 *E. coli/Shigella* study strains were performed using the Aligner algorithm of the MAUVE program, version 2.0.0 [98], with the following parameters: –island-size = 20 –backbone-size = 20 –max-backbone-gap = 20 –seed-size = 19 –gapped-aligner = clustal –max-gapped-aligner-length = 10000 –min-recursive-gap-length = 5000 –weight = 5000. The MAUVE output file was further treated so as to assign each part of the alignment to either one of two categories, ‘backbone’ or ‘variable segment’ (previously named ‘loops’), as described [99]. Briefly, regions not belonging to a “match”, as defined by MAUVE and less than 10 kbp long were aligned using ClustalW and the alignment was automatically inspected. The region was considered as a backbone segment if all pairwise comparisons gave more than 76% identity, with never more than 20 consecutive gaps. In all other cases, the entire region was considered as a variable segment.

To produce the DNA alignment file from the above mentioned procedure, the coordinates of all backbone segments on each genome were extracted and aligned with MAFFT, version 6.24 [100], using a home made Perl script. Segments were first aligned with the ‘–globalpair option’, which is suitable for a suite of globally alignable sequences. When problems occurred (especially for long backbone segments), MAFFT alignments were computed using the ‘–auto’ option which automatically selects an appropriate alignment algorithm according to data size.

Statistical analysis along the chromosome (scans). Along the chromosomal multiple genome alignment we studied the variation of descriptive statistics, such as the GC% and estimates of the mutation and recombination rates. We estimated each statistics, F, on a sliding window of constant size along the concatenated alignment. We then estimated the average value of the statistic μ and its standard deviation 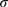 with the median and the inter quartile distance (normalised by a factor of 1.38) as these estimates are less affected by the existence of extremes values. We then calculated the standardised cumulative sum along the genome 
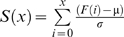
. When the cumulative sum is decreasing in a region, it means that this region harbours a lower than average value of the statistics. Hence for each statistics we can identify the boundaries of regions having atypical values.

### Phylogenetic Analyses

To reconstruct the phylogeny of the strains, we used two data sets: the genes common to all the *E. coli/Shigella* and *E. fergusonii* strains (*Escherichia* core genome) and the genome backbone, defined as above. We also used several methods for each dataset.

(i) The reference phylogenetic tree of the *Escherichia* core genome genes was reconstructed from the concatenated alignments of 1878 genes of the core genome of the *E. coli/Shigella* and *E. fergusonii* strains. We used Tree-puzzle 5.2 [101] to compute the distance matrix between all strains using maximum likelihood under the HKY+gamma (with 8 categories)+I model. The tree was then built from the distance matrix using BioNJ [102]. We made 1000 bootstrap experiments on the concatenated sequences to assess the robustness of the topology. (ii) We also inferred a tree for each of the 1878 genes in the core genome, using maximum likelihood with PHYML 2.4.4 with a GTR+gamma+I model for each gene [103]. For each tree we extracted the relevant parameters of the model and made a weighted average to obtain a global average model. We used the lengths of the genes as weights of the average. The global model thus obtained was used to infer a tree based on the concatenation of the genes using Tree-puzzle 5.2. The tree was then built from the matrix of distances using the BioNJ algorithm. To check that the branch lengths obtained with this method were correct we computed them by maximum likelihood by imposing the tree topology (baseML from package PAML 4 implementation [104]). The differences found were extremely small. To assess the robustness of the tree we bootstrapped 1000 times the concatenated sequences, each time launching Tree-puzzle with the same previously inferred global model. (iii) We performed comparisons among phylogenetic trees. To test if the phylogenetic tree of each gene (as inferred by maximum likelihood using the PHYML 2.4.4: GTR+gamma+I model) is significantly different from the global tree as reconstructed from the concatenation of genes of the *Escherichia* core genome, we performed several tests for comparing tree topologies using likelihood. These included a SH test [105], two types of Kishino and Hasegawa test (KH test) (i.e., the original two-sided KH test as described in [106] and the one-sided KH test [107] using pairwise SH tests), and the expected likelihood weights (ELW) [108]. For the simulations, we used these tests as well as the Robinson and Foulds test [45]. All tests used a 5% significance criterion. (iv) We also built a consensus tree (extended majority rule as implemented in CONSENSE) using PHYLIP 3.66 package [109] from the set of trees inferred in (ii).

Using MAUVE's global alignment we also extracted a backbone concatenate which we input into Tree-puzzle with the HKY+gamma (with 8 categories)+I model to obtain a matrix of distances. BioNJ was then used to reconstruct the unrooted tree from the distance matrix.

### Phylogenetic Congruence Along the Chromosome

Using the chromosomal multiple genome alignment, we studied the likelihood of the species tree for any 5 kbp window of conserved sequence along the genome. Since the likelihood, as estimated with PHYML [103] under the HKY model, depends on both the length of the sequence studied and the fraction of informative polymorphic sites, we computed the regression between the number of sites and the likelihood for sequences of same size, then estimated a score as the deviation from that prediction. Hence, a phylogenetic score of 0 reflects a region for which the likelihood of the species tree equals the average across all the genome. A negative score reflects a lower than average likelihood, i.e., the phylogeny is affected more than average by recombination.

### Coalescent Simulations

We simulated 2 million, 3 kbp sequences under a neutral coalescent framework with pure gene conversion using the MS software [110]. All simulations had different values of the per-base rate of mutation (theta), the per-base gene conversion rate (C_gc_) and the average tract length (L_gc_) (assuming a geometrical distribution). For each of these simulations, statistics of linkage disequilibrium specific to the gene conversion signature were calculated as described elsewhere [39]. Basically long distance and short distance linkage disequilibrium are measured for pairs and triplets of sites. Since we had previously estimated fairly small gene conversion tract lengths [42], we used window sizes of 1 kbp, 0.2 kbp and 0.1 kbp, instead of the larger default values.

Using ABCest software [41], an Approximate Bayesian Computation method, we estimated these parameters for all the genes of the genome and all the 3 kbp sliding windows along the genome alignment with a step of 500 bp. To assess the reliability of the method we tested it on 1500 new simulations. The Pearson correlation between the observed and estimated ratio C_gc_/theta was very high (0.897, 0.885 for the log transformed values) and 92% of simulations provided a 95% confidence interval around the estimated value encompassing the true value. Tract length, L_gc_, provided quite large 95% confidence intervals so even if 92% of simulations encompassed the real value in this interval, the Pearson correlation between observed and estimated value was lower: 0.585 (0.676 for the log transformed values). Hence, this approach provides adequate estimates of the parameters and once the 2 million simulations have been performed, it allows a rapid (several seconds) estimation of the parameters for each dataset.

To study how gene conversion affected the phylogenetic reconstruction process, we modified the MS software [110] to allow 25 kbp of sequences evolve in a pure gene conversion model, but maintaining 1 nucleotide without any conversion so that its history reflects the history of the chromosomal backbone. We then compared with various methods (see Phylogenetic analyses section) the topology of the phylogenetic tree as reconstructed with PHYML [103] from the 25 kbp, as evolved along MS-derived local topologies under the HKY model with Seq-Gen [111], with the true history of the non-recombining last nucleotide, as directly extracted from MS.

### Estimation of Ancestral Characters

We used the function “ACE” (package “APE” in R [112]) to estimate ancestral character states for continuous (genome size) and discrete (presence or absence of genes) characters on all branches of tree involving these taxa. For continuous characters we used a Brownian motion model in which characters evolve following a random walk. This model was fitted by least squares [113]. We estimated ancestral discrete characters by maximum likelihood [114]. For this we built a matrix wherein the number of rows corresponds to the number of characters (i.e., 18 822 positional ortholog genes corresponding to the pan-genome) and the number of columns corresponds to the number of genomes (i.e., 1 *E. fergusoni* and 20 *E. coli* strains). The model has two character states (0 = absence of the gene, 1 = presence of the gene). Since genome sizes are relatively constant among the closely related genera *Escherichia*, *Salmonella* and *Yersinia*, we assumed a probability of insertion equal to the probability of deletion, i.e., we assumed that genomes are close to equilibrium in terms of genome size. Variations in size are thus seen as stochastic fluctuations associated with the insertion of certain large elements such as phages.

We used the reference phylogenetic tree and the phyletic pattern indicating the presence/absence of each gene (of the pan-genome) to infer the probability of presence of each gene in each internal node of the tree. For each such node a gene was considered as present if it had a probability of presence ≥0.5. The numbers of genes lost and gained, respectively, were then determined in the following way: if the gene was absent (vs. present) in a given node but present (vs. absent) in its ancestor, it was considered as gained (vs. lost) along the branch leading to the given node. Ancestral gene order was determined on all branches of tree using the parsimony criterion. Considering the internal node gene order, the numbers of acquisition and loss events was defined for sets of consecutive pairs of genes (by allowing gaps of 1 gene).

The number of events in each branch of the species tree was computed by reconstructing the relative order of the core genes in the ancestral genome by parsimony. We then combined in a single event the contiguous gains or losses of genes in the same branch, allowing gaps of 1 gene.

### Mouse Lethality Assay

A mouse model of systemic infection was used to assess the intrinsic extraintestinal virulence of the available strains [23]. For each strain, 10 outbred female Swiss OF1 mice (3–4 weeks old, 14–16 gm) were challenged subcutaneously in the abdomen with a standardized bacterial inoculum (0.2 ml of Ringer solution with 10^9^ cfu/ml of log-phase bacteria). Mortality was assessed over 7 days post-challenge. In this model system, lethality is a rather clear-cut parameter and, based on the number of mice killed, almost all strains were classified as non-killer (<2 of 10 mice killed) or killer (>8 mice killed) [32].

## Supporting Information

Figure S1 Circular representation of the six *Escherichia coli* genomes and the *E. fergusonii* genome. Circles display from the inside out: (1) GC skew (G+C/G−C using a 1 kbp sliding window). (2) Location of tRNA genes, rRNA operons and Insertion Sequences (ISs). (3) GC deviation (difference between the mean GC content in a 1 kbp window and the overall mean GC). Red areas indicate that the deviation is greater than 2 standard deviations. (4) Ancestral E. coli genome. Yellow areas denote genes that are present in all the genomes under study. (5) Scale. (6) Gene specificity at strain level. Genes sharing at least one homolog in an other *E. coli* strain of the same phylogenetic group and having more than 85% identity over at least 80% of their length were regarded as non specific. To simplify the visualisation of specific regions, we created a colour gradient that denotes the percentage of organisms that possess a homolog of a given gene within the reference genome. If this particular gene is present in all the organisms under study, it is tagged in light grey. Conversely, if it is present only in the reference genome, it is tagged in dark colour. In other words, the more pronounced the colour, the higher the specificity. (7) Gene specificity at group level. The same criteria were used as for circle (6) but the genome analysed is compared to *E. coli* strains that belong to other phylogenetic groups. The comparison includes *Shigella* as well. (8) Gene specificity at the species level. The same protocol was used as for circles (6) and (7) except the comparison involves *E. fergusonii* which is considered as the outgroup for this study.
(1.82 MB PPT)

Figure S2 Visual representation of MAUVE multiple alignment of 20 *Escherichia coli/Shigella* genomes. The representation was performed using the MOSAIC database (http://genome.jouy.inra.fr/mosaic/) multiple alignment viewer. Horizontal lines correspond to a linear representation of each genome sequence drawn to scale. The blue line corresponds to annotated genes. (At this scale only a unique line is visible.) Coloured blocks correspond to the locally collinear blocks (LCBs) of the alignment as defined by MAUVE. LCBs corresponding to inversions are represented on a second line. An LCB in one genome is linked to the corresponding LCB on the subsequent genome with a plot of the same colour. This visual representation shows that, apart from the rearrangements present in *Shigella* chromosomes, *E. coli* genomes are mostly collinear.
(0.11 MB PPT)

Figure S3 Phylogenetic tree of the backbone of the 20 *Escherichia coli* and *Shigella* strains as reconstructed by MAUVE software. This unrooted tree was built using Tree-puzzle with the HKY+gamma (with 8 categories)+I model followed by BioNJ to reconstruct the tree from the distance matrix. The values at the nodes correspond to support values for each internal branch, as estimated by Tree-puzzle (range 0–100), and can be interpreted in much the same way as bootstrap values.
(0.09 MB PPT)

Figure S4 Association between gene repertoire relatedness and phylogenetic distance. A. Genomes were binned according to phylogenetic distance for clarity. For the first two bins, which correspond to the most closely related genes, there is a high percentage of genome in common, which is not the case for the other bins, which correspond with more distantly related genes. B. Histogram of the phylogenetic distances between pairs of genomes.
(0.04 MB PPT)

Figure S5 Reconstruction of gains and losses of genes in the evolution of *Escherichia coli*. The cladogram shows the phylogenetic relationships between the 20 *E. coli/Shigella* genomes rooted on the *E. fergusonii* genome, as in Figure 4, with branch lengths ignored for clarity. Each strain and internal node of the tree is labelled with the inferred numbers of genes gained (red: top) and lost (black: top), and the inferred numbers of corresponding events of gene acquisition (red: bottom) and loss (black: bottom) along the branch. Pie charts on each branch indicate the functional classification of genes lost, using the colour-scale (details in the keys). The functional classes of known-function genes are represented by numbers explained by a key in Supplementary Table 4.
(0.31 MB PPT)

Figure S6 Association between the distance of a node to the tip of the tree and the difference between the predicted ancestral genome size and the effective number of genes reliably predicted to be present in the node. The association is highly significant (R^2^ = 0.56, p<0.001).
(0.03 MB PPT)

Figure S7 Characteristics of hotspots of insertion/deletion of genetic material. The circles indicate the values per location between contiguous genes in the core genome. Data are (from the outside circle inwards): average number of genes, standard deviation, sum of genes, number of prophage-like elements, number of insertion sequence like elements, sum of tRNA genes and heterogeneity rate at hotspots. The latter is the ratio between the observed number of orthologs and the expected value if all genes had orthologs in all genomes, after excluding genomes lacking genes at the hotspot and those for which the region has a synteny breakpoint.
(0.14 MB PPT)

Figure S8 Phylogenetic congruence at the *rfb* locus coding for O antigen. We followed the likelihood of the species topology for 5 kbp windows (spaced by 250 bp) along the chromosomal backbone. After correcting for the number of polymorphic sites, each window received a Z score of phylogenetic congruence. Low values reflect lower than average phylogenetic congruence. A large region (green arrow) has a significantly lower congruence than the rest of the genome. The red arrows indicate the hotspots of integration and the corresponding loci when identified. HPI: high pathogenicity island.
(0.03 MB PPT)

Figure S9 Distribution of Tajima's D statistics on the 1976 *Escherichia coli* core genome genes. The colour code is as follows: all mutations (red), synonymous mutations (green) and non-synonymous mutations (yellow). Negative Tajima's D values [126] reflect a higher than expected frequency of rare alleles. The more negative value of Tajima's D for non-synonymous mutations suggests that they are on average deleterious: they persist some time in populations before selection removes them.
(0.08 MB PPT)

Table S1 Pseudogenes found in *Escherichia coli* K-12 MG1655 and the 7 newly sequenced genomes of the ColiScope project. The ‘Reference’ column give the coding sequence (CDS) identifier of the wild type form of the gene, in one of the 21 analyzed *Escherichia* and *Shigella* bacterial genomes. For each of these 21 genomes the particular gene's status is indicated as functional (‘1’), absent (‘0’) or a pseudogene (‘−1’). Gene names in boldface correspond to genes that are pseudogenes only in the considered strain.
(0.25 MB XLS)

Table S2 A) Number of predicted protein encoding genes in the genomes of the newly sequenced strains of *Escherichia coli* and *E. fergusonii*. Genes were (a) functionality annotated using automatic annotation transfer from K-12 MG1655 orthologs or other ColiScope manually annotated orthologous genes, (b) manually annotated using the MaGe web-based graphical interface, or (c) considered as false positive gene predictions. B) Publicly available *Escherichia* and *Shigella* genomes included in the ColiScope database. (a) Inaccurate (‘Wrong’ status) or missed gene annotations (‘New’ status) have been found using our MICheck procedure. For the 14 analyzed genomes, the list of newly predicted genes is given in Supplementary Table 3. (b) Automatic functional annotation transfer between orthologous genes (85% identity over at least 80% of the length of the smallest protein) began with similarity results obtained with *E. coli* K-12 MG1655, then with the new genomes of the ColiScope project. False gene predictions (i.e., artefacts) were those defined in the course of the expert annotation of the ColiScope sequences. (c) ‘Specific genes’ are genes that have no ortholog in *E. coli* K-12 MG1655 or any of the newly sequenced and annotated genomes.
(0.05 MB DOC)

Table S3 Missing genes in publicly available *Escherichia coli* and *Shigella* genomes. The genes are ordered by length (given in base pairs). Those that are similar to genes from the minimal gene set defined by [127] are highlighted in boldface. Functional descriptions of the genes, starting with ‘fragment of’ (Product column), are provided for putative pseudogenes (whether actual pseudogenes or sequencing errors). For some *E. coli* stains, e.g., UTI89, the corresponding pseudogenes were probably correctly annotated by the authors (numbering of the gene locus_tags), but were not reported in the databank files (GeneBank and EMsBL), and thus were annotated as missing genes by the MICheck procedure [97].
(0.36 MB XLS)

Table S4 Synteny blocks and insertion sequence (IS) elements among the 21 *Escherichia coli/Shigella/E. fergusonii* genomes.
(0.03 MB DOC)

Table S5 Classification of bioprocesses (key for Figure 7). The tests indicate the sense of the difference in the number of genes associated with a given bioprocess. ‘+/−’ means more/fewer genes in the first class, i.e., more/fewer in the core genome than in the complementary set. ‘++/−−’ means the difference is significant at the 5% level, using a Chi square test followed by a sequential Bonferroni correction for multiple tests.
(0.02 MB DOC)

Table S6 Genes (and associated characteristics) categorically associated with certain phylogenetic groups or pathotypes. Principal characteristics of the genes were deduced from the annotation process.
(0.23 MB DOC)

Table S7 Genes of the genomic island at the *pheV* tRNA insertion hot spot (see Figure 9).
(0.08 MB XLS)
