# Supplementary figures and images for: Organised Genome Dynamics in the Escherichia coli Species Results in Highly Diverse Adaptive Paths
Source: PLoS Genet. 2009 Jan 23;5(1):e1000344. doi: 10.1371/journal.pgen.1000344 (PMC2617782; doi:10.1371/journal.pgen.1000344)

## Slide 1
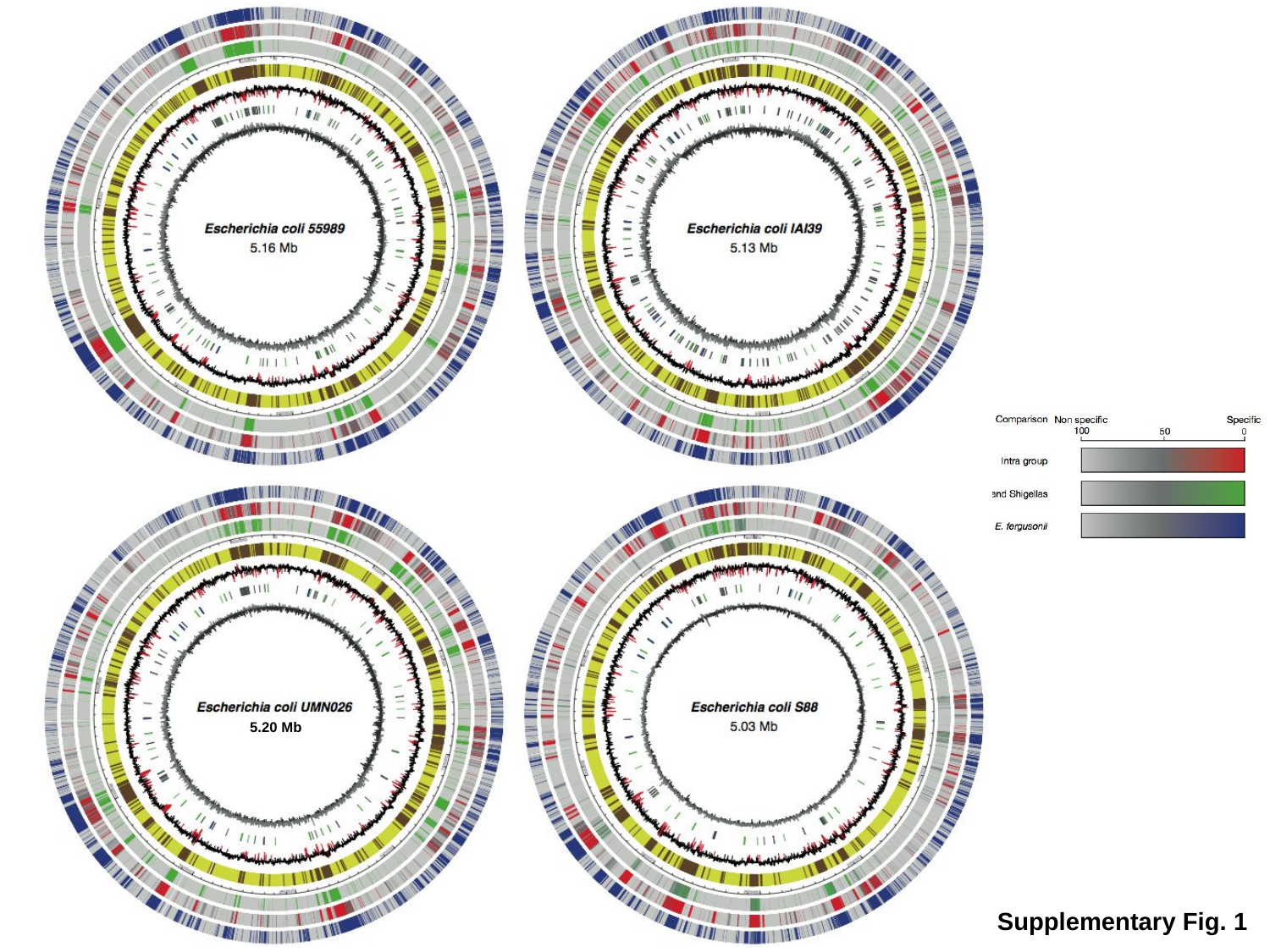

5.20 Mb
Supplementary Fig. 1

## Slide 2
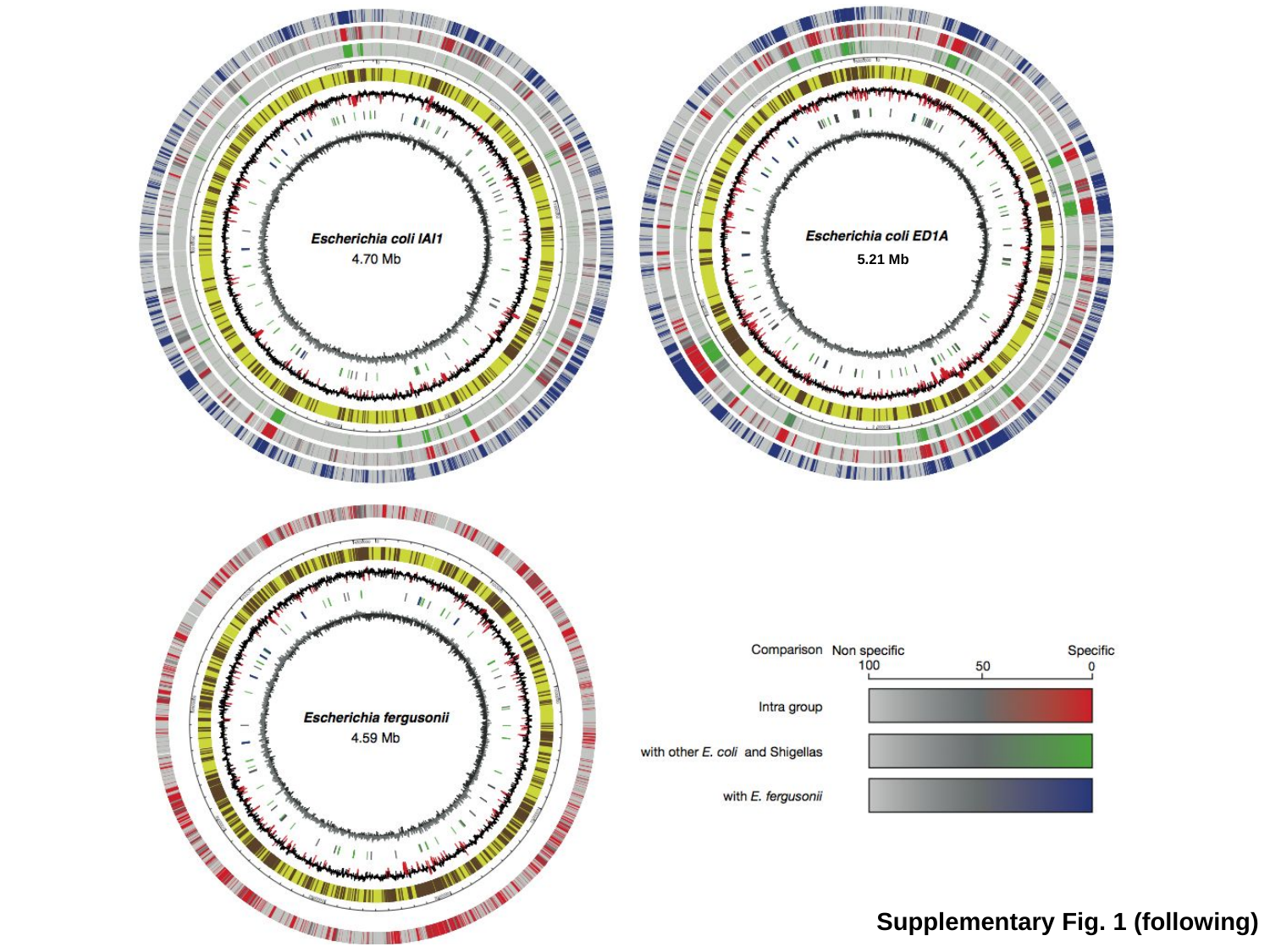

5.21 Mb
Supplementary Fig. 1 (following)

Supplement: Figure S1 — Circular representation of the six Escherichia coli genomes and the E. fergusonii genome. Circles display from the inside out: (1) GC skew (G+C/G−C using a 1 kbp sliding window). (2) Location of tRNA genes, rRNA operons and Insertion Sequences (ISs). (3) GC deviation (difference between the mean GC content in a 1 kbp window and the overall mean GC). Red areas indicate that the deviation is greater than 2 standard deviations. (4) Ancestral E. coli genome. Yellow areas denote genes that are present in all the genomes under study. (5) Scale. (6) Gene specificity at strain level. Genes sharing at least one homolog in an other E. coli strain of the same phylogenetic group and having more than 85% identity over at least 80% of their length were regarded as non specific. To simplify the visualisation of specific regions, we created a colour gradient that denotes the percentage of organisms that possess a homolog of a given gene within the reference genome. If this particular gene is present in all the organisms under study, it is tagged in light grey. Conversely, if it is present only in the reference genome, it is tagged in dark colour. In other words, the more pronounced the colour, the higher the specificity. (7) Gene specificity at group level. The same criteria were used as for circle (6) but the genome analysed is compared to E. coli strains that belong to other phylogenetic groups. The comparison includes Shigella as well. (8) Gene specificity at the species level. The same protocol was used as for circles (6) and (7) except the comparison involves E. fergusonii which is considered as the outgroup for this study. (1.82 MB PPT) [file pgen.1000344.s001.ppt]

## Slide 1
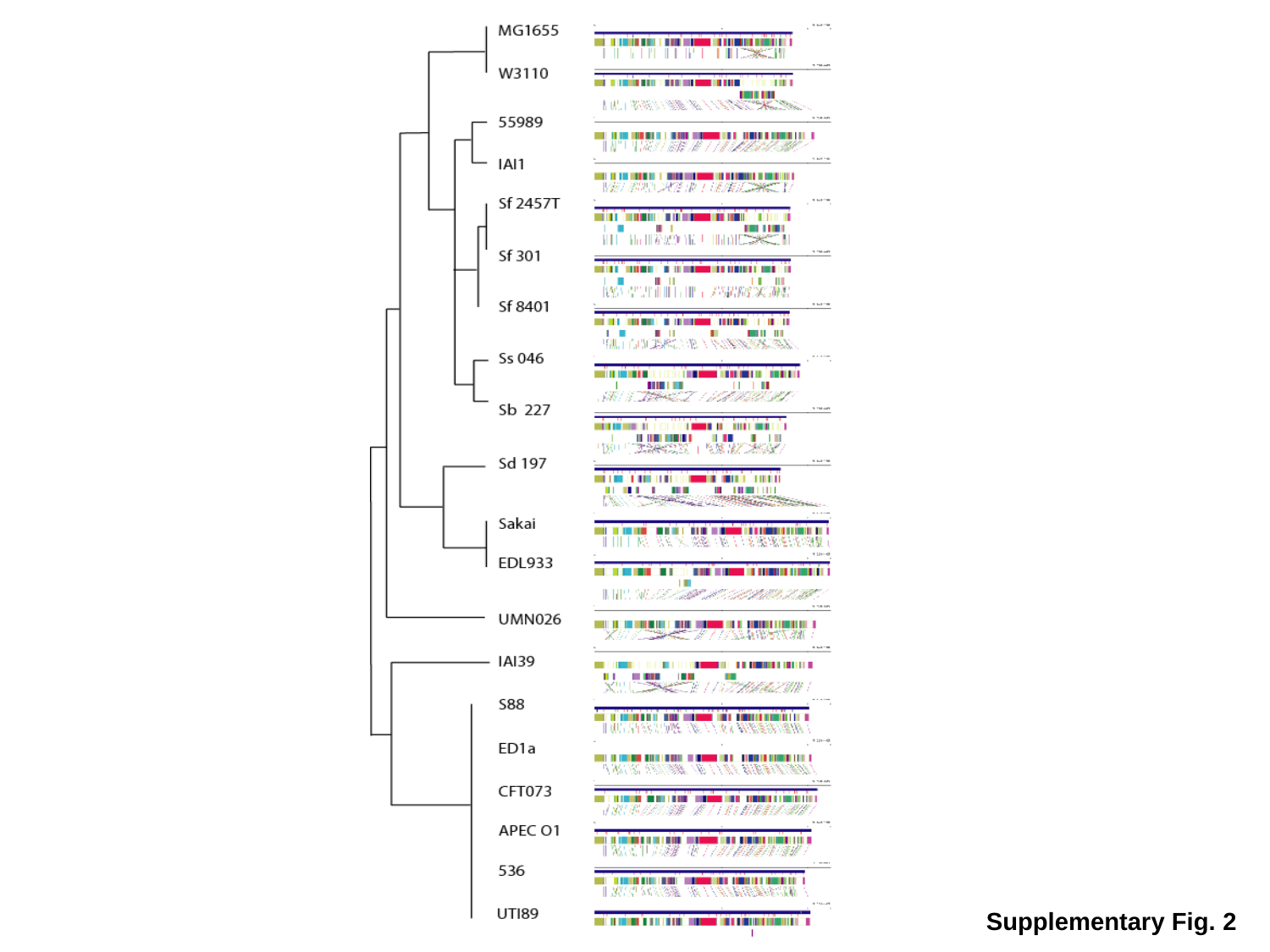

Supplementary Fig. 2

Supplement: Figure S2 — Visual representation of MAUVE multiple alignment of 20 Escherichia coli/Shigella genomes. The representation was performed using the MOSAIC database (http://genome.jouy.inra.fr/mosaic/) multiple alignment viewer. Horizontal lines correspond to a linear representation of each genome sequence drawn to scale. The blue line corresponds to annotated genes. (At this scale only a unique line is visible.) Coloured blocks correspond to the locally collinear blocks (LCBs) of the alignment as defined by MAUVE. LCBs corresponding to inversions are represented on a second line. An LCB in one genome is linked to the corresponding LCB on the subsequent genome with a plot of the same colour. This visual representation shows that, apart from the rearrangements present in Shigella chromosomes, E. coli genomes are mostly collinear. (0.11 MB PPT) [file pgen.1000344.s002.ppt]

## Slide 1
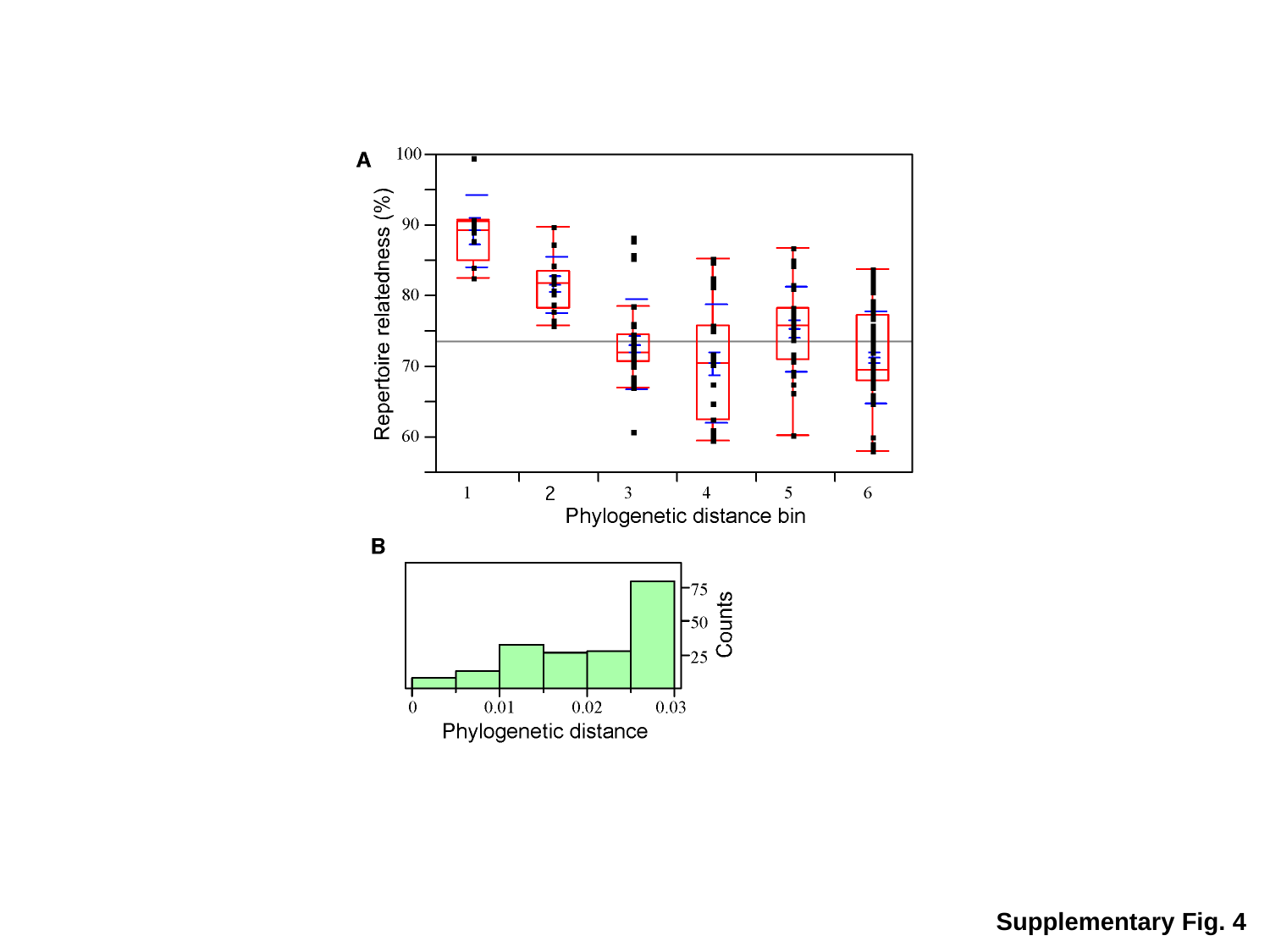

Supplementary Fig. 4

Supplement: Figure S4 — Association between gene repertoire relatedness and phylogenetic distance. A. Genomes were binned according to phylogenetic distance for clarity. For the first two bins, which correspond to the most closely related genes, there is a high percentage of genome in common, which is not the case for the other bins, which correspond with more distantly related genes. B. Histogram of the phylogenetic distances between pairs of genomes. (0.04 MB PPT) [file pgen.1000344.s004.ppt]

## Slide 1
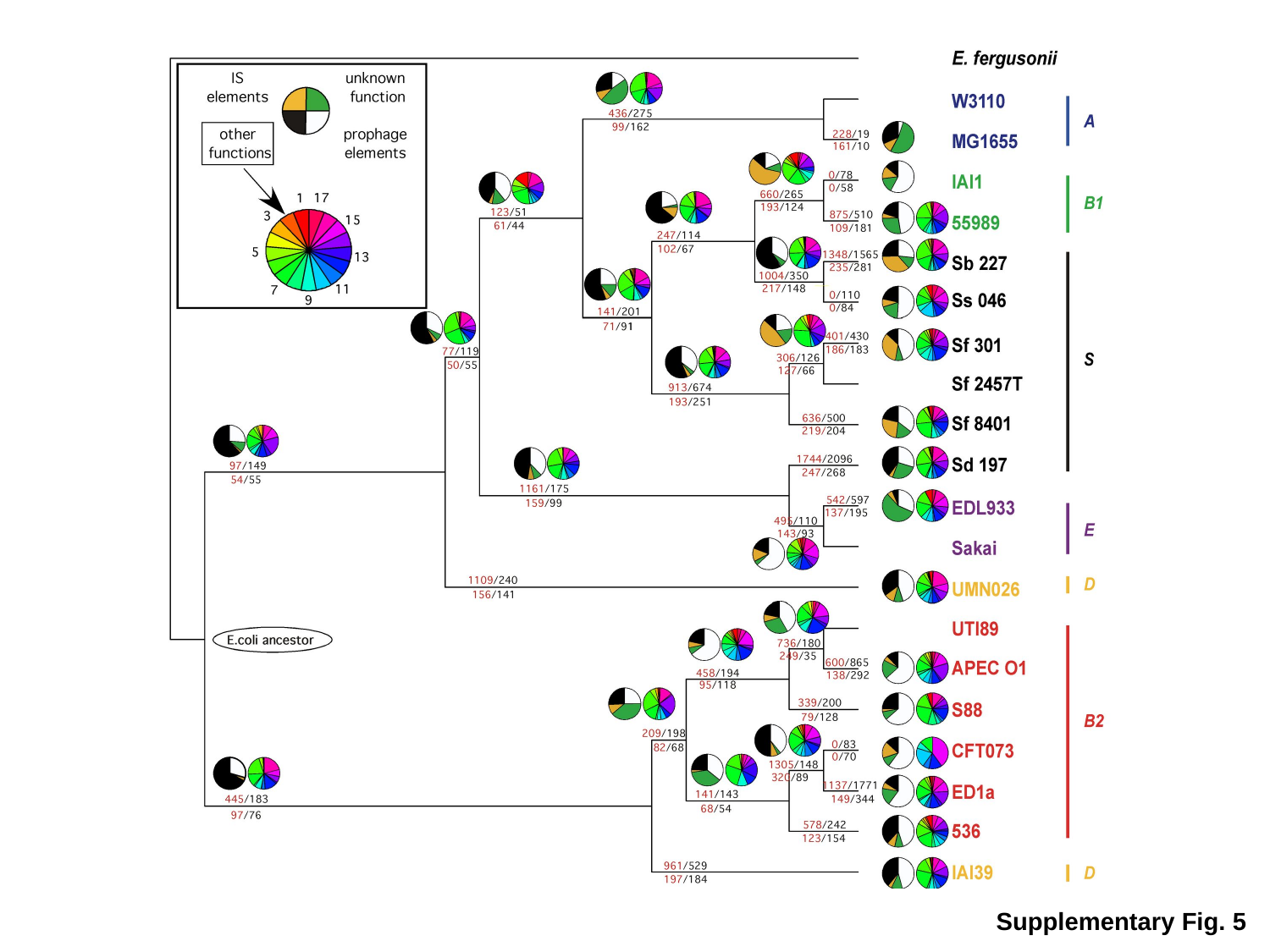

Supplementary Fig. 5

Supplement: Figure S5 — Reconstruction of gains and losses of genes in the evolution of Escherichia coli. The cladogram shows the phylogenetic relationships between the 20 E. coli/Shigella genomes rooted on the E. fergusonii genome, as in Figure 4, with branch lengths ignored for clarity. Each strain and internal node of the tree is labelled with the inferred numbers of genes gained (red: top) and lost (black: top), and the inferred numbers of corresponding events of gene acquisition (red: bottom) and loss (black: bottom) along the branch. Pie charts on each branch indicate the functional classification of genes lost, using the colour-scale (details in the keys). The functional classes of known-function genes are represented by numbers explained by a key in Supplementary Table 4. (0.31 MB PPT) [file pgen.1000344.s005.ppt]

## Slide 1
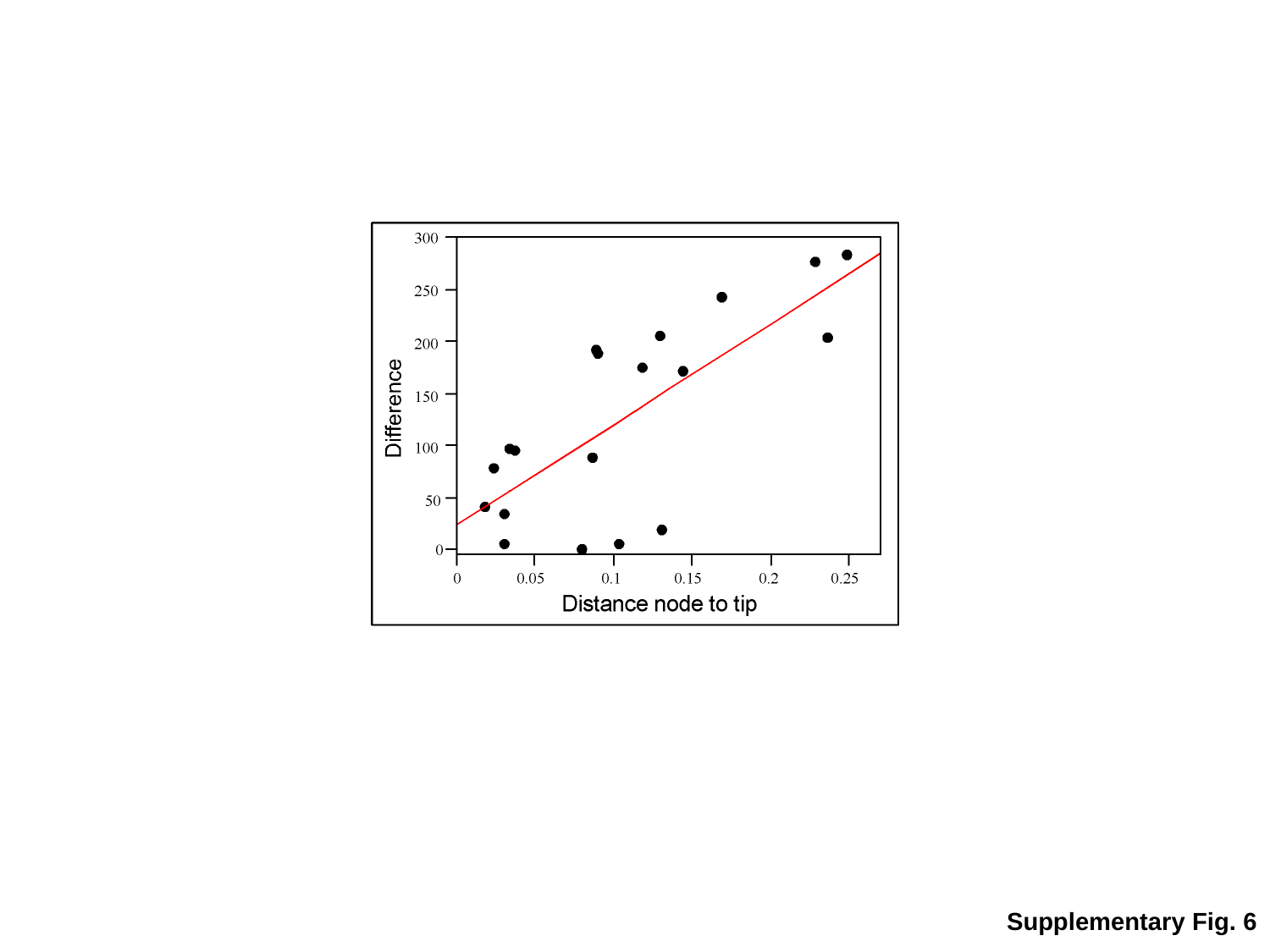

Supplementary Fig. 6

Supplement: Figure S6 — Association between the distance of a node to the tip of the tree and the difference between the predicted ancestral genome size and the effective number of genes reliably predicted to be present in the node. The association is highly significant (R2 = 0.56, p<0.001). (0.03 MB PPT) [file pgen.1000344.s006.ppt]

## Slide 1
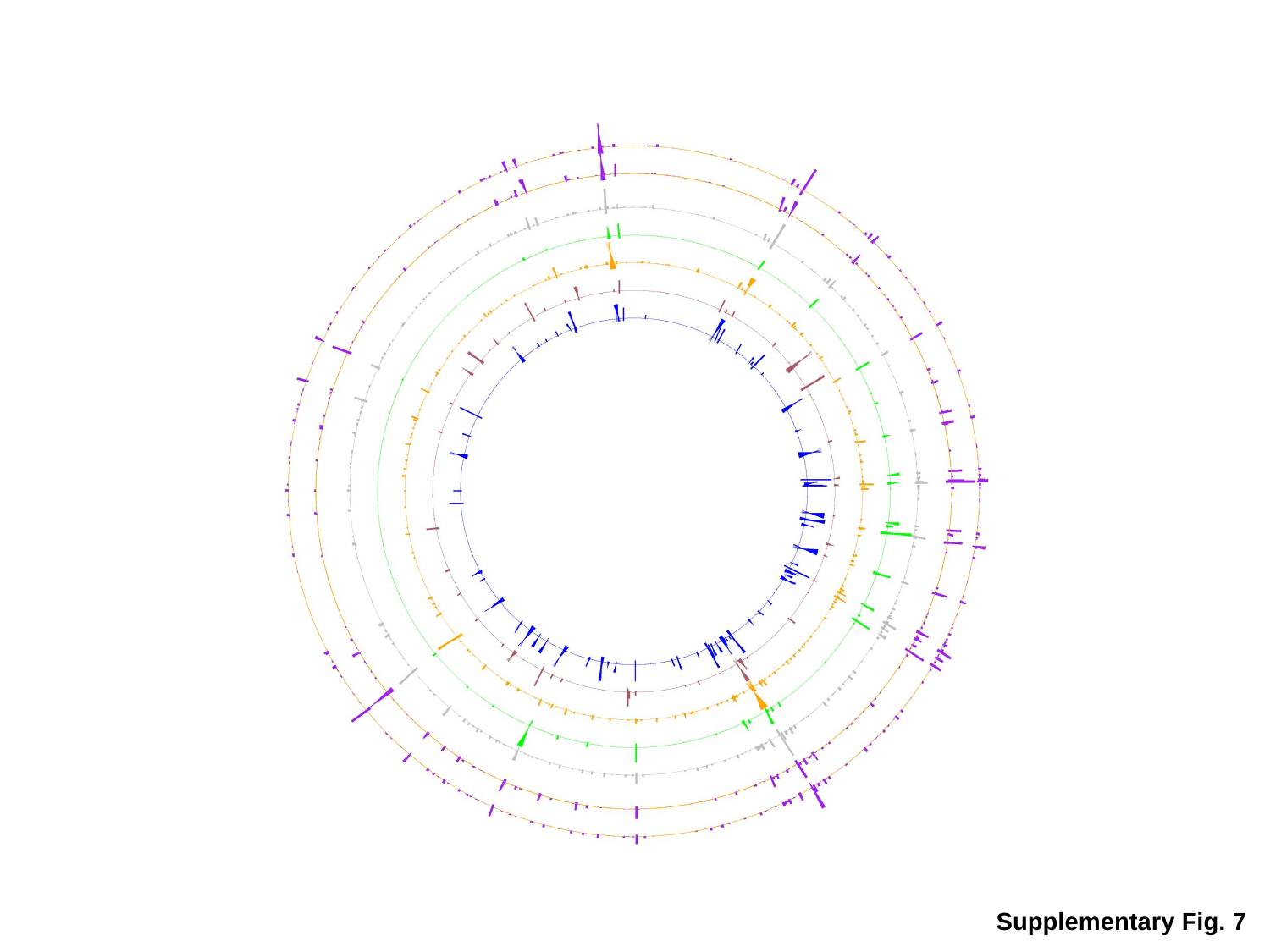

Supplementary Fig. 7

Supplement: Figure S7 — Characteristics of hotspots of insertion/deletion of genetic material. The circles indicate the values per location between contiguous genes in the core genome. Data are (from the outside circle inwards): average number of genes, standard deviation, sum of genes, number of prophage-like elements, number of insertion sequence like elements, sum of tRNA genes and heterogeneity rate at hotspots. The latter is the ratio between the observed number of orthologs and the expected value if all genes had orthologs in all genomes, after excluding genomes lacking genes at the hotspot and those for which the region has a synteny breakpoint. (0.14 MB PPT) [file pgen.1000344.s007.ppt]
