# Supplementary material for: Organised Genome Dynamics in the Escherichia coli Species Results in Highly Diverse Adaptive Paths
Source: PLoS Genet. 2009 Jan 23;5(1):e1000344. doi: 10.1371/journal.pgen.1000344 (PMC2617782; doi:10.1371/journal.pgen.1000344)
Supplement: Figure S3 — Phylogenetic tree of the backbone of the 20 Escherichia coli and Shigella strains as reconstructed by MAUVE software. This unrooted tree was built using Tree-puzzle with the HKY+gamma (with 8 categories)+I model followed by BioNJ to reconstruct the tree from the distance matrix. The values at the nodes correspond to support values for each internal branch, as estimated by Tree-puzzle (range 0–100), and can be interpreted in much the same way as bootstrap values. (0.09 MB PPT) [file pgen.1000344.s003.ppt]

## Slide 1
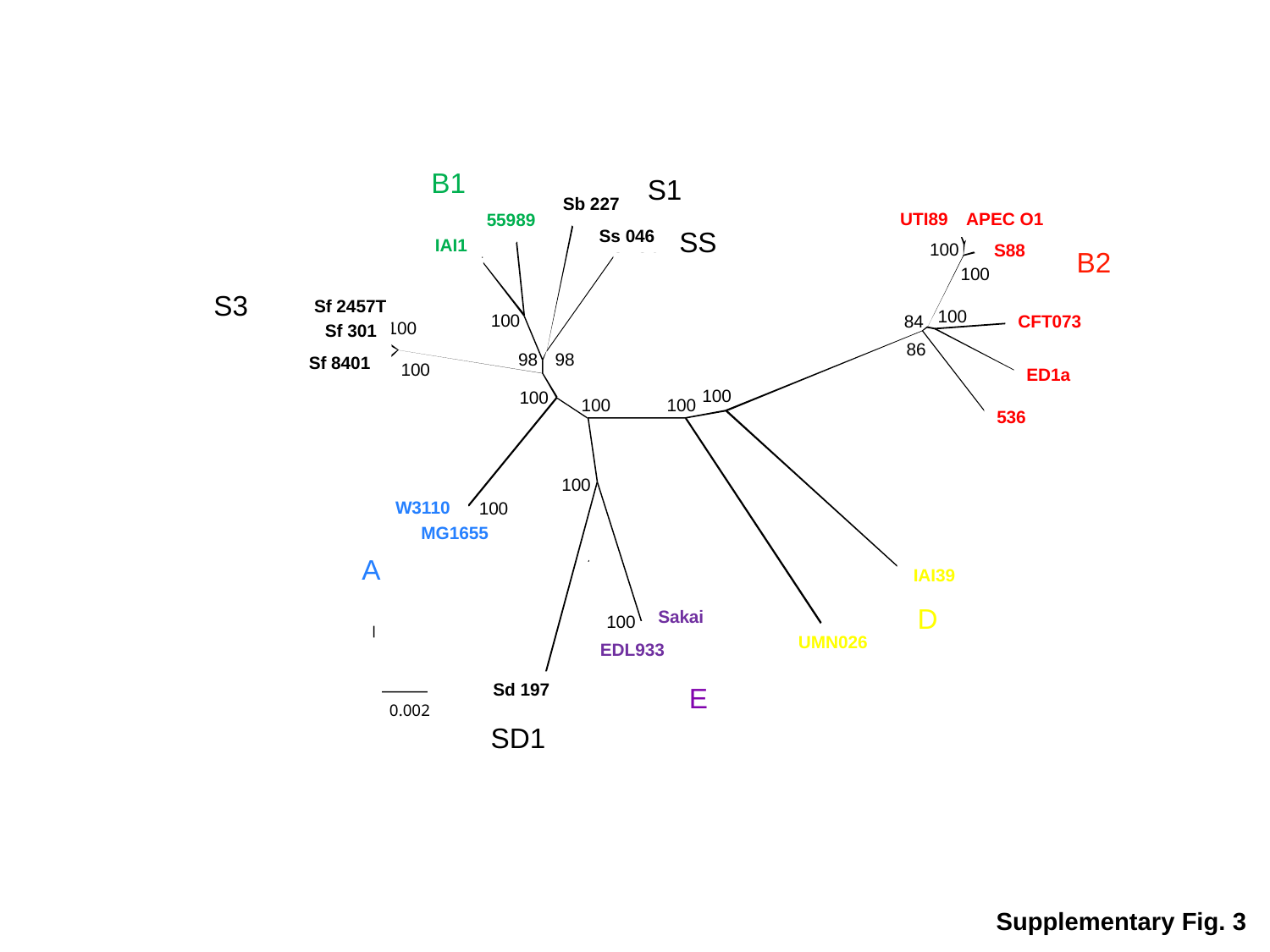

B1
S1
Sb 227
 SHBOA
UTI89
APEC O1
55989
ESCON
ESCOG
 ESCOI
Ss 046
SS
IAI1
100
S88
 SHSOA
B2
ESCOL
100
S3
Sf 2457T
100
100
84
CFT073
100
Sf 301
86
98
98
Sf 8401
100
ED1a
 ESCOM
100
100
100
100
536
100
W3110
100
ESCOA
MG1655
ESCOF
A
IAI39
 ESCOO
D
Sakai
100
UMN026
 ESCOK
EDL933
ESCOB
Sd 197
E
0.002
SD1
Supplementary Fig. 3
