# Supplementary material for: Organised Genome Dynamics in the Escherichia coli Species Results in Highly Diverse Adaptive Paths
Source: PLoS Genet. 2009 Jan 23;5(1):e1000344. doi: 10.1371/journal.pgen.1000344 (PMC2617782; doi:10.1371/journal.pgen.1000344)
Supplement: Figure S8 — Phylogenetic congruence at the rfb locus coding for O antigen. We followed the likelihood of the species topology for 5 kbp windows (spaced by 250 bp) along the chromosomal backbone. After correcting for the number of polymorphic sites, each window received a Z score of phylogenetic congruence. Low values reflect lower than average phylogenetic congruence. A large region (green arrow) has a significantly lower congruence than the rest of the genome. The red arrows indicate the hotspots of integration and the corresponding loci when identified. HPI: high pathogenicity island. (0.03 MB PPT) [file pgen.1000344.s008.ppt]

## Slide 1
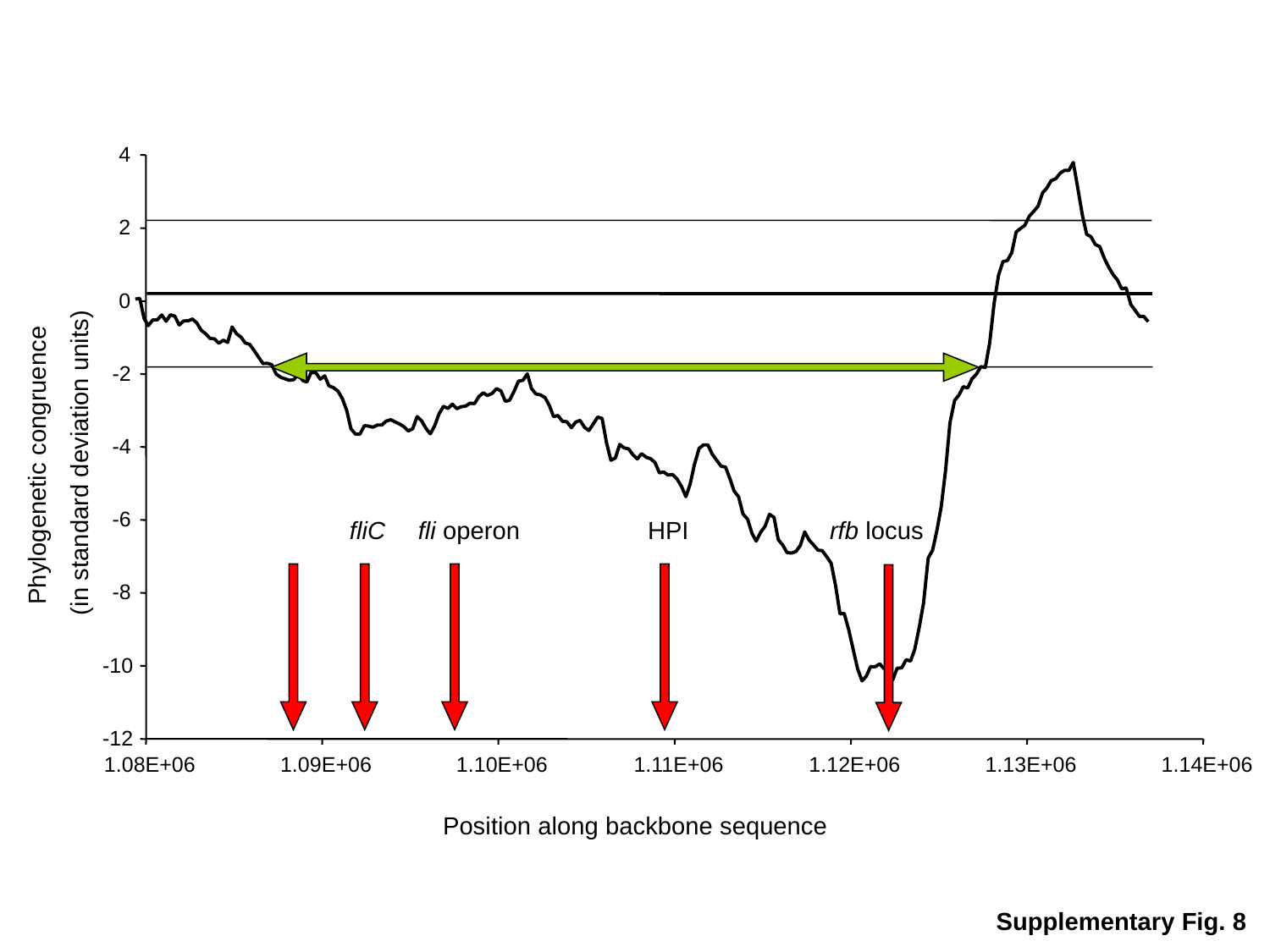

4
2
0
-2
-4
Phylogenetic congruence
 (in standard deviation units)
-6
fliC
fli operon
HPI
rfb locus
-8
-10
-12
1.08E+06
1.09E+06
1.10E+06
1.11E+06
1.12E+06
1.13E+06
1.14E+06
Position along backbone sequence
Supplementary Fig. 8
