# Supplementary material for: Organised Genome Dynamics in the Escherichia coli Species Results in Highly Diverse Adaptive Paths
Source: PLoS Genet. 2009 Jan 23;5(1):e1000344. doi: 10.1371/journal.pgen.1000344 (PMC2617782; doi:10.1371/journal.pgen.1000344)
Supplement: Figure S9 — Distribution of Tajima's D statistics on the 1976 Escherichia coli core genome genes. The colour code is as follows: all mutations (red), synonymous mutations (green) and non-synonymous mutations (yellow). Negative Tajima's D values [126] reflect a higher than expected frequency of rare alleles. The more negative value of Tajima's D for non-synonymous mutations suggests that they are on average deleterious: they persist some time in populations before selection removes them. (0.08 MB PPT) [file pgen.1000344.s009.ppt]

## Slide 1
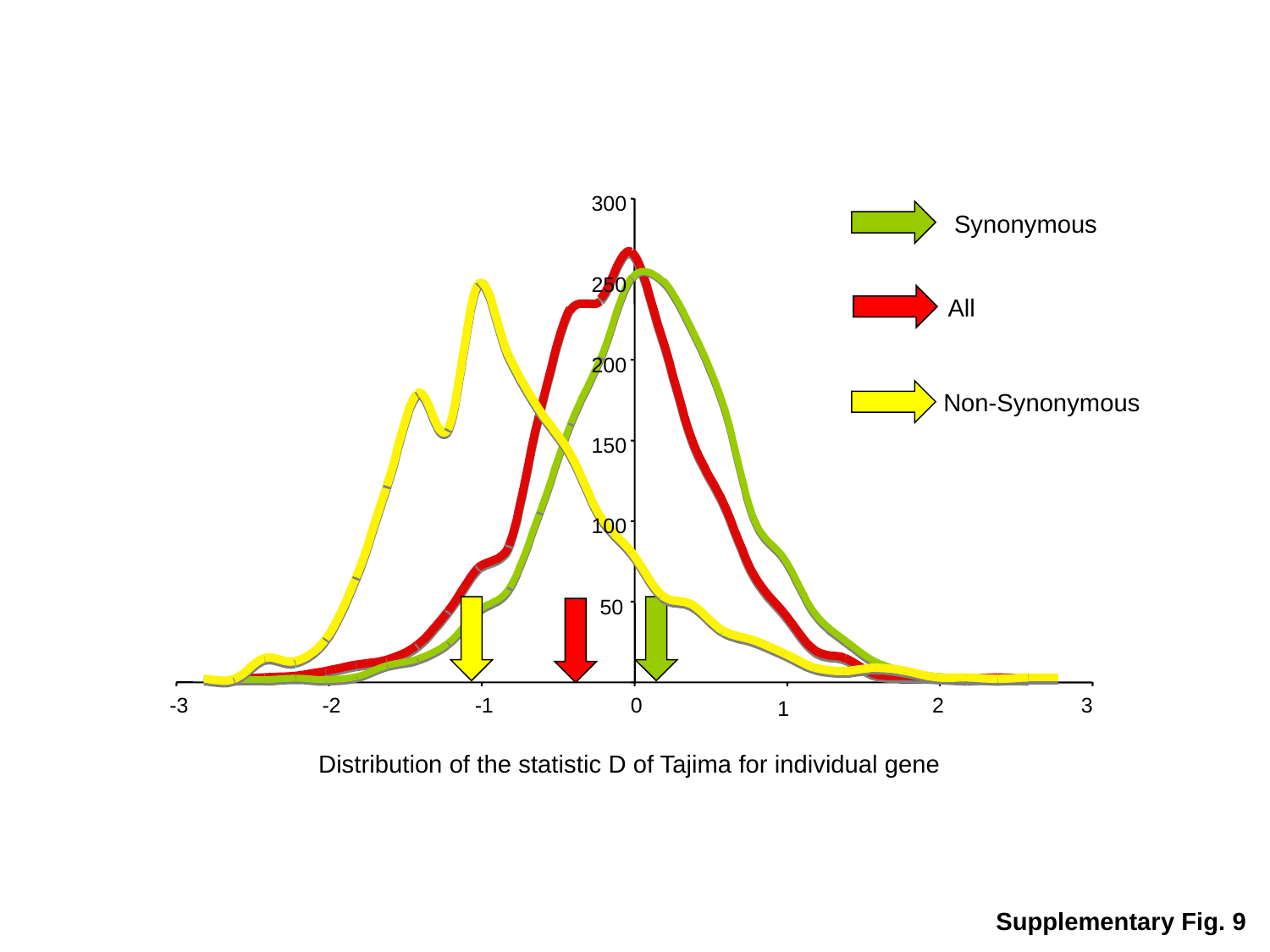

300
Synonymous
250
All
200
Non-Synonymous
150
100
50
2
3
-3
-2
-1
0
1
Distribution of the statistic D of Tajima for individual gene
Supplementary Fig. 9
