# Supplementary material for: Organised Genome Dynamics in the Escherichia coli Species Results in Highly Diverse Adaptive Paths
Source: PLoS Genet. 2009 Jan 23;5(1):e1000344. doi: 10.1371/journal.pgen.1000344 (PMC2617782; doi:10.1371/journal.pgen.1000344)
Supplement: Table S2 — A) Number of predicted protein encoding genes in the genomes of the newly sequenced strains of Escherichia coli and E. fergusonii. Genes were (a) functionality annotated using automatic annotation transfer from K-12 MG1655 orthologs or other ColiScope manually annotated orthologous genes, (b) manually annotated using the MaGe web-based graphical interface, or (c) considered as false positive gene predictions. B) Publicly available Escherichia and Shigella genomes included in the ColiScope database. (a) Inaccurate (‘Wrong’ status) or missed gene annotations (‘New’ status) have been found using our MICheck procedure. For the 14 analyzed genomes, the list of newly predicted genes is given in Supplementary Table 3. (b) Automatic functional annotation transfer between orthologous genes (85% identity over at least 80% of the length of the smallest protein) began with similarity results obtained with E. coli K-12 MG1655, then with the new genomes of the ColiScope project. False gene predictions (i.e., artefacts) were those defined in the course of the expert annotation of the ColiScope sequences. (c) ‘Specific genes’ are genes that have no ortholog in E. coli K-12 MG1655 or any of the newly sequenced and annotated genomes. (0.05 MB DOC) [file pgen.1000344.s011.doc]

|  | Size(Mb) | Predicted protein genes | Automaticannotation transfer^a^ | Manual expert annotation^b^ | **Artefacts**^c^ |
| --- | --- | --- | --- | --- | --- |
| ***E. coli* S88** Chr.  Plas. | 5.032  0.134 | 5086  157 | 2909  - | 1950  144 | 227  13 |
| ***E. coli* UMN026** Chr.  Plas1  Plas2 | 5.202  0.122  0.034 | 5048  160  50 | 3534  -  - | 1384  149  49 | 130  11  1 |
| ***E. coli* IAI1** Chr. | 4.701 | 4627 | 3658 | 833 | 136 |
| ***E. coli* ED1a** Chr.  Plas. | 5.209  0.120 | 5275  153 | 3833  - | 1296  150 | 146  3 |
| ***E. coli* 55989** Chr.  Plas. | 5.155  0.072 | 5065  106 | 3841  - | 1128  100 | 96  6 |
| ***E. coli* IAI39** Chr. | 5.132 | 4935 | 4225 | 681 | 29 |
| ***E. fergusonii*** Chr.  Plas. | 4.589  0.055 | 4501  58 | 2481  - | 1855  54 | 165  4 |
| **TOTAL ANNOTATION EFFORT:** | | | | **9776** |  |

**Supplementary Table 2A. Number of predicted protein encoding genes in the genomes of the newly sequenced strains of *Escherichia coli* and *E. fergusonii.***

Genes were (a) functionality annotated using automatic annotation transfer from K-12 MG1655 orthologs or other ColiScope manually annotated orthologous genes, (b) manually annotated using the MaGe web-based graphical interface, or (c) which were considered as false positive gene predictions.

Chr : chromosome

Plas : plasmid

| Genomes integrated in the ColiScope **database** | Size(Mb) | Original data | | | Re -annotation **Process ^a^** | | Annotation transfer ^b^ | | | Specific genes^c^ |
| --- | --- | --- | --- | --- | --- | --- | --- | --- | --- | --- |
|  |  | Date | RefSeq | Genes (nb) | ‘New’ status | ‘Wrong’ status | *E. coli* K12 | *E. coli* strains from ColiScope | Artefacts |  |
| ***E. coli* O157:H7 EDL** | 5.53 | 2001-01 | NC_002655 | 5374 | 74 | 94 | 3739 | 921 | 25 | 669 |
| ***E. coli* O157:H7 Sakai** | 5.50 | 2001-02 | NC_002695 | 5269 | 164 | 102 | 3757 | 911 | 26 | 637 |
| ***E. coli* CFT073** | 5.23 | 2002-12 | NC_004431 | 5443 | 72 | 525 | 3568 | 1079 | 56 | 287 |
| ***E. coli* W3110** | 4.64 | 2006-03 | NC_000091 | 4352 | 5 | 0 | 4199 | 54 | 3 | 101 |
| ***E. coli* UTI89** | 5.06 | 2006-04 | NC_007946 | 5029 | 88 | 354 | 3554 | 1016 | 34 | 159 |
| ***E. coli* 536** | 4.94 | 2006-07 | NC_008253 | 4668 | 40 | 24 | 3603 | 744 | 17 | 320 |
| ***E. coli* APECO1** | 5.08 | 2006-10 | NC_008563 | 4461 | 389 | 126 | 3564 | 1009 | 37 | 114 |
| ***E. coli* HS** | 4.64 | 2007-09 | NC_009800 | 4577 | 109 | 221 | 3788 | 478 | 13 | 186 |
| ***S. flexneri* 301** | 4.61 | 2002-10 | NC_004337 | 4656 | 136 | 11 | 3753 | 490 | 53 | 485 |
| ***S. flexneri* 2457T** | 4.60 | 2003-04 | NC_004741 | 4668 | 178 | 56 | 3785 | 472 | 53 | 480 |
| ***S. boydii* Sb227** | 4.52 | 2005-11 | NC_007613 | 4542 | 213 | 27 | 3612 | 486 | 47 | 583 |
| ***S. sonnei* Ss046** | 4.82 | 2005-11 | NC_007384 | 4585 | 379 | 14 | 3762 | 533 | 57 | 598 |
| ***S. dysenteria* Sd197** | 4.37 | 2005-11 | NC_007606 | 4649 | 187 | 117 | 3592 | 478 | 52 | 597 |
| ***S. flexneri* 5b 8401** | 4.57 | 2006-07 | NC_008258 | 4522 | 153 | 16 | 3688 | 491 | 48 | 432 |

**Supplementary Table 2B.** **Publicly available *Escherichia* and *Shigella* genomes included in the ColiScope database.**

(a) Inaccurate (‘Wrong’ status) or missed gene annotations (‘New’ status) have been found using our MICheck procedure. For the 14 analyzed genomes, the list of newly predicted genes is given in Supplementary Table 3. (b) Automatic functional annotation transfer between orthologous genes (85 % identity over at least 80 % of the length of the smallest protein) began with similarity results obtained with *E. coli* K-12 MG1655, then with the new genomes of the ColiScope project. False gene predictions (i.e, artefacts) were those defined in the course of the expert annotation of the ColiScope sequences. (c) ‘Specific genes’ are genes that have no ortholog in *E. coli* K-12 MG1655 or any of the newly sequenced and annotated genomes.
