# Supplementary material for: Organised Genome Dynamics in the Escherichia coli Species Results in Highly Diverse Adaptive Paths
Source: PLoS Genet. 2009 Jan 23;5(1):e1000344. doi: 10.1371/journal.pgen.1000344 (PMC2617782; doi:10.1371/journal.pgen.1000344)
Supplement: Table S4 — Synteny blocks and insertion sequence (IS) elements among the 21 Escherichia coli/Shigella/E. fergusonii genomes. (0.03 MB DOC) [file pgen.1000344.s013.doc]

**Supplementary Table 4**. **Synteny blocks and insertion sequence (IS) elements among the 21 *Escherichia coli / Shigella/ E. fergusonii* genomes.**

| Strains | Chromosome size, bp | No. of syntenic blocks | No. of  IS-like elements |
| --- | --- | --- | --- |
| *E. fergusonii* | 4,588,714 | 26 | 29 |
| MG1655 | 4,639,675 | 1 | 66 |
| W3110 | 4,646,332 | 2 | 93 |
| IAI1 | 4,700,561 | 1 | 42 |
| 55989 | 5,154,863 | 1 | 150 |
| *S. boydii* 4 227 | 4,519,823 | 33 | 848 |
| *S. sonnei* 046 | 4,825,265 | 17 | 717 |
| *S. flexneri* 2a 301 | 4,607,203 | 20 | 574 |
| *S. flexneri* 2a 2457T | 4,599,354 | 21 | 549 |
| *S. flexneri* 5b 8401 | 4,574,284 | 26 | 575 |
| *S. dysenteriae* 1 197 | 4,369,232 | 65 | 1155 |
| O157:H7 EDL933 | 5,528,445 | 2 | 130 |
| O157:H7 Sakai | 5,498,450 | 1 | 117 |
| UMN026 | 5,195,774 | 1 | 92 |
| UTI89 | 5,065,741 | 3 | 61 |
| APEC O1 | 5,082,025 | 1 | 45 |
| S88 | 5,032,269 | 1 | 47 |
| CFT073 | 5,231,428 | 1 | 111 |
| ED1A | 5,092,707 | 1 | 118 |
| 536 | 4,938,920 | 1 | 108 |
| IAI39 | 5,132,071 | 11 | 224 |
