# Supplementary material for: Organised Genome Dynamics in the Escherichia coli Species Results in Highly Diverse Adaptive Paths
Source: PLoS Genet. 2009 Jan 23;5(1):e1000344. doi: 10.1371/journal.pgen.1000344 (PMC2617782; doi:10.1371/journal.pgen.1000344)
Supplement: Table S5 — Classification of bioprocesses (key for Figure 7). The tests indicate the sense of the difference in the number of genes associated with a given bioprocess. ‘+/−’ means more/fewer genes in the first class, i.e., more/fewer in the core genome than in the complementary set. ‘++/−−’ means the difference is significant at the 5% level, using a Chi square test followed by a sequential Bonferroni correction for multiple tests. (0.02 MB DOC) [file pgen.1000344.s014.doc]

**Supplementary Table 5**. **Classification of bioprocesses (key for Fig. 7).**

| Bioprocesses | Ancestral genome  (core vs non-core) | Pan-genome  (core vs non-core) | Pan-genome  (ancestral vs non-anc) |
| --- | --- | --- | --- |
| 1 Amino acid biosynthesis | ++ | ++ | ++ |
| 2 Purines, pyrimidines, nucleosides, and nucleotides | ++ | ++ | ++ |
| 3 Fatty acid and phospholipid metabolism | + | ++ | ++ |
| 4 Biosynthesis of cofactors, prosthetic groups | ++ | ++ | ++ |
| 5 Central intermediary metabolism | - | - | ++ |
| 6 Energy metabolism | - | + | ++ |
| 7 Transport and binding proteins | -- | -- | - |
| 8 DNA metabolism | ++ | ++ | + |
| 9 Transcription | + | + | + |
| 10 Protein synthesis | ++ | ++ | ++ |
| 11 Protein fate | + | + | + |
| 12 Regulatory functions | - | - | - |
| 13 Signal transduction | - | - | - |
| 14 Cell envelope | -- | -- | -- |
| 15 Cellular processes | - | - | + |
| 16 Biological processes | -- | -- | -- |
| 17 Mobile and extrachromosomal element functions | + | -- | -- |

The tests indicate the sense of the difference in the number of genes associated with a given bioprocess. '+/-' means more/less genes in the first class, i.e., more/less in the core genome than in the complementary set. '++/--' means the difference is significant at the 5 % level, using a Chi square test followed by a sequential Bonferroni correction for multiple tests.
