# Supplementary material for: Organised Genome Dynamics in the Escherichia coli Species Results in Highly Diverse Adaptive Paths
Source: PLoS Genet. 2009 Jan 23;5(1):e1000344. doi: 10.1371/journal.pgen.1000344 (PMC2617782; doi:10.1371/journal.pgen.1000344)
Supplement: Table S6 — Genes (and associated characteristics) categorically associated with certain phylogenetic groups or pathotypes. Principal characteristics of the genes were deduced from the annotation process. (0.23 MB DOC) [file pgen.1000344.s015.doc]

**Supplementary Table 6.** **Genes (and associated characteristics) categorically associated with certain phylogenetic groups or pathotypes.**

Principal characteristics of the genes were deduced from the annotation process.

Genes present in the A group strains

| **Gene** | **Product** | **ProductType** | **Roles** | **BioProcess** | **Locus_tag** (from K12 strain) |
| --- | --- | --- | --- | --- | --- |
| *insL* | IS186/IS421 transposase | h : extrachromosomal origin | 8.3 : Transposon related ; | 17.3 : Transposon functions ; | ECK0016-1 |
| *ykgN* | putative transposase component; CP4-6 prophage | pe : putative enzyme | 8.1 : Prophage genes and phage related functions ; 8 : extrachromosomal ; | 17.2 : Prophage functions ; | ECK0266 |
| *ynfN* | hypothetical protein; Qin prophage | h : extrachromosomal origin | 8.1 : Prophage genes and phage related functions ; | 17.2 : Prophage functions ; | ECK1545 |
| *ydhL* | conserved hypothetical protein | o : ORF of unknown function | | 18 : Unknown function ; | ECK1644 |
| *yfbN* | hypothetical protein | o : ORF of unknown function | | 18 : Unknown function ; | ECK2267 |
| *yfbO* | hypothetical protein | o : ORF of unknown function | | 18 : Unknown function ; | ECK2268 |
| *yfbP* | hypothetical protein | o : ORF of unknown function | | 18 : Unknown function ; | ECK2269 |
| *yfcQ* | putative fimbrial-like adhesin exported protein | ps : putative structure | 6.5 : Pilus ; | 16.8 : Protect ; 14.1 : Surface structures ; | ECK2328 |
| *yfeS* | conserved hypothetical protein | o : ORF of unknown function | | 18 : Unknown function ; | ECK2415 |
| *ygcH* | hypothetical protein | o : ORF of unknown function | | 18 : Unknown function ; | ECK2751 |
| *ygcI* | hypothetical protein | o : ORF of unknown function | | 18 : Unknown function ; | ECK2752 |
| *ygcJ* | hypothetical protein | o : ORF of unknown function | | 18 : Unknown function ; | ECK2753 |
| *ygcK* | hypothetical protein | o : ORF of unknown function | | 18 : Unknown function ; | ECK2754 |
| *ygcL* | hypothetical protein | o : ORF of unknown function | | 18 : Unknown function ; | ECK2755 |
| *ygcB* | conserved hypothetical protein; putative member of DEAD box family | pe : putative enzyme | | 18 : Unknown function ; | ECK2756 |
| *ygeO* | hypothetical protein | o : ORF of unknown function | | 18 : Unknown function ; | ECK2857 |
| *tdcR* | DNA-binding transcriptional activator | r : regulator | 1.1.3 : Amino acids ; 2.2.2 : Transcription related | 6.2 : Amino acids and amines | ECK3108 |
| *hokA* | toxic polypeptide, small | f : factor |  | 15.8 : Toxin production and resistance ; | ECK3544 |
| *yifO* | conserved hypothetical protein | o : ORF of unknown function | | 18 : Unknown function ; | ECK3768 |

Genes absent in the A group strains

| **Gene** | **Product** | **ProductType** | **Roles** | **BioProcess** | **Locus_tag** (from IAI1 strain) |
| --- | --- | --- | --- | --- | --- |
|  | Exonuclease | e : enzyme |  |  | ECIAI1_0756 |
|  | putative host-nuclease inhibitor protein Gam of bacteriophage | h : extrachromosomal origin | 8.1 : Prophage genes and phage related functions ; | 17.2 : Prophage functions ; | ECIAI1_0758 |
|  | Recombination protein bet | e : enzyme |  | 8.1 : DNA replication, recombination, and repair ; | ECIAI1_0757 |
| *V* | Major tail protein V | h : extrachromosomal origin | 8.1.6 : Structural component ; 8.1 : Prophage genes and phage related functions ; | 17.2 : Prophage functions ; | ECIAI1_0792 |
|  | Minor tail protein L | h : extrachromosomal origin | 8.1.6 : Structural component ; 8.1 : Prophage genes and phage related functions ; | 17.2 : Prophage functions ; | ECIAI1_0798 |
| *K* | Tail assembly protein K | h : extrachromosomal origin | 8.1.1 : DNA packaging, phage assembly ; 8.1 : Prophage genes and phage related functions ; | 17.2 : Prophage functions ; | ECIAI1_0799 |
|  | Host specificity protein J | h : extrachromosomal origin | 8.1 : Prophage genes and phage related functions ; | 17.2 : Prophage functions ; | ECIAI1_0801 |
| *G* | Minor tail protein G | h : extrachromosomal origin | 8.1.6 : Structural component ; 8.1 : Prophage genes and phage related functions ; | 17.2 : Prophage functions ; | ECIAI1_0793 |
|  | Minor tail protein precursor H | h : extrachromosomal origin | 8.1.6 : Structural component ; 8.1 : Prophage genes and phage related functions ; | 17.2 : Prophage functions ; | ECIAI1_0795 |
|  | Minor tail protein M | h : extrachromosomal origin | 8.1.6 : Structural component ; 8.1 : Prophage genes and phage related functions ; | 17.2 : Prophage functions ; | ECIAI1_0797 |
| *fII* | Tail attachment protein (Minor capsid protein FII) | h : extrachromosomal origin | 8.1.6 : Structural component ; | 17.2 : Prophage functions ; | ECIAI1_0789 |
| *U* | Minor tail protein U | h : extrachromosomal origin | 8.1.6 : Structural component ; 8.1 : Prophage genes and phage related functions ; | 17.2 : Prophage functions ; | ECIAI1_0791 |
|  | conserved hypothetical protein | o : ORF of unknown function | | 18 : Unknown function ; | ECIAI1_3048 |
|  | conserved hypothetical protein; putative membrane protein | o : ORF of unknown function | 6.1 : Membrane ; 4 : Transport ; | 7 : Transport and binding proteins ; | ECIAI1_3049 |
|  | conserved hypothetical protein; putative exported protein | o : ORF of unknown function | | 18 : Unknown function ; | ECIAI1_3050 |
|  | putative ATP-binding protein of ABC transport system | pe : putative enzyme | 4.3.A.1.a : ATP binding component ; | 7 : Transport and binding proteins ; | ECIAI1_3052 |
|  | conserved hypothetical protein; putative exported protein | o : ORF of unknown function | | 18 : Unknown function ; | ECIAI1_3070 |
|  | putative enzyme | o : ORF of unknown function | | 18.1 : Enzymes of unknown specificity ; | ECIAI1_3149 |
| *phnE* | membrane channel protein component of Pn transporter | t : transporter | 1.8.1 : Phosphorous metabolism ; | 5.3 : Phosphorus compounds ; | ECIAI1_4333 |

Genes present in the B1 group strains

| **Gene** | **Product** | **ProductType** | **Roles** | **BioProcess** | **Locus_tag** (from IAI1 strain) |
| --- | --- | --- | --- | --- | --- |
| *yafU* | Inner membrane protein yafU | m : membrane component |  | 14 : Cell envelope ; | ECIAI1_0226 |
|  | conserved hypothetical protein (Hcp-like) | o : ORF of unknown function |  | 18 : Unknown function ; | ECIAI1_0227 |
|  | conserved hypothetical protein | o : ORF of unknown function |  | 18 : Unknown function ; | ECIAI1_0268 |
|  | conserved hypothetical protein | o : ORF of unknown function |  | 18 : Unknown function ; | ECIAI1_0269 |
|  | transposase, IS110 family | e : enzyme | 8.3 : Transposon related ; 8.3.1 : transposases ; | 17.3 : Transposon functions ; | ECIAI1_0337 |
|  | putative integral membrane protein | pm : putative membrane component |  | 14 : Cell envelope ; | ECIAI1_0822 |
|  | conserved hypothetical protein; putative exported protein | o : ORF of unknown function |  | 18 : Unknown function ; | ECIAI1_1185 |
|  | conserved hypothetical protein; putative exported protein | o : ORF of unknown function |  | 18 : Unknown function ; | ECIAI1_1462 |
|  | conserved hypothetical protein | o : ORF of unknown function |  | 18 : Unknown function ; | ECIAI1_1861 |
|  | conserved hypothetical protein | o : ORF of unknown function |  | 18 : Unknown function ; | ECIAI1_2271 |
|  | putative 2-dehydro-3-deoxyglucarate aldolase (yfaU) (partial) | pe : enzyme |  |  | ECIAI1_2323 |
|  | conserved hypothetical protein, putative Zn-dependent exopeptidases | o : ORF of unknown function |  | 18 : Unknown function ; | ECIAI1_2348 |
|  | conserved hypothetical protein, putative nucleotide binding protein | o : ORF of unknown function |  | 18 : Unknown function ; | ECIAI1_2525 |
|  | conserved hypothetical protein, putative transcriptionnal regulation protein | o : ORF of unknown function |  | 18 : Unknown function ; | ECIAI1_2526 |
| *ypjB* | conserved hypothetical protein | o : ORF of unknown function |  | 18 : Unknown function ; | ECIAI1_2750 |
| *ypjC* | conserved hypothetical protein | o : ORF of unknown function |  | 18 : Unknown function ; | ECIAI1_2751 |
| *eprI* | Putative Type III secretion EprI protein | pt : putative transporter | 4.3.A.6 : The Type III (Virulence-related) Secretory Pathway (IIISP) Family ; | 7 : Transport and binding proteins ; | ECIAI1_2973 |
| *lpfD* | LpfD protein precursor | m : membrane component |  | 14 : Cell envelope ; | ECIAI1_3708 |
|  | hypothetical protein | o : ORF of unknown function |  | 18 : Unknown function ; | ECIAI1_3850 |
|  | hypothetical protein | o : ORF of unknown function |  | 18 : Unknown function ; | ECIAI1_3998 |
|  | conserved hypothetical membrane protein (fragment) | o : ORF of unknown function |  | 18 : Unknown function ; | ECIAI1_4010 |
|  | putative zeta toxin; poison-antidote element | pf : putative factor |  | 18 : Unknown function ; | ECIAI1_4248 |
|  | conserved hypothetical protein | o : ORF of unknown function |  | 18 : Unknown function ; | ECIAI1_4608 |

Genes absent in the B1 group strains

| **Gene** | **Product** | **ProductType** | **Roles** | **BioProcess** | **Locus_tag** (from K12 strain) |
| --- | --- | --- | --- | --- | --- |
| *ykiA* | hypothetical protein | o : ORF of unknown function |  | 18 : Unknown function ; | ECK0387 |
| *yciQ* | conserved hypothetical protein; putative inner membrane protein | pm : putative membrane component |  | 18 : Unknown function ; | ECK1262 |
| *yfaU* | putative 2,4-dihydroxyhept-2-ene-1,7-dioic acid-like aldolase | pe : putative enzyme | 1.7.1 : Unassigned reversible reactions ; | 16.1 : Circulate ; 5 : Central intermediary metabolism ; | ECK2238 |
| *ygiS* | transporter subunit: periplasmic-binding component of ABC superfamily | t : transporter | 4.3.A.1.p : periplasmic binding component ; 7.2 : Periplasmic space ; | 7 : Transport and binding proteins ; | ECK3011 |
| *aslA* | arylsulfatase-like enzyme | e : enzyme | 1.8.2 : Sulfur metabolism | 16.2 : Construct biomass (Anabolism) | ECK3794 |

Genes present in the B2 group strains

| **Gene** | **Product** | **ProductType** | **Roles** | **BioProcess** | **Label Locus_tag** (from S88 strain) |
| --- | --- | --- | --- | --- | --- |
|  | Putative Colicin E7 immunity protein (ImmE7) | pf : putative factor |  | 15 : Cellular processes ; | ECS88_0116 |
|  | transposase InsA (ORF1) IS1 | pe : putative enzyme | 8.3 : Transposon related ; 8.3.1 : transposases ; | 17.3 : Transposon functions ; | ECS88_0281 |
|  | Putative adhesin; putative outer membrane autotransporter barrel | pm : putative membrane component |  | 14 : Cell envelope ; | ECS88_0312 |
|  | putative membrane protein | pm : putative membrane component |  | 18 : Unknown function ; | ECS88_0523 |
|  | conserved hypothetical protein | o : ORF of unknown function |  | 18 : Unknown function ; | ECS88_0530 |
|  | conserved hypothetical protein, TPR repeat protein | o : ORF of unknown function |  | 18 : Unknown function ; | ECS88_0755 |
| *ybiA* | conserved hypothetical protein | o : ORF of unknown function |  | 18 : Unknown function ; | ECS88_0816 |
|  | conserved hypothetical protein | o : ORF of unknown function |  | 18 : Unknown function ; | ECS88_1232 |
|  | conserved hypothetical protein | o : ORF of unknown function |  | 18 : Unknown function ; | ECS88_1233 |
|  | Putative HTH-type transcriptional regulator | pr : putative regulator | 3.1.2 : Transcriptional level ; | 12.1 : DNA interactions ; | ECS88_1479 |
|  | hypothetical protein | o : ORF of unknown function |  | 18 : Unknown function ; | ECS88_1556 |
|  | conserved hypothetical protein; putative exported protein | o : ORF of unknown function | 10 : cryptic genes ; | 18 : Unknown function ; | ECS88_2180 |
|  | conserved hypothetical protein | o : ORF of unknown function | 10 : cryptic genes ; | 18 : Unknown function ; | ECS88_2181 |
|  | conserved hypothetical protein | o : ORF of unknown function | 10 : cryptic genes ; | 18 : Unknown function ; | ECS88_2182 |
|  | conserved hypothetical protein | o : ORF of unknown function | 10 : cryptic genes ; | 18 : Unknown function ; | ECS88_2183 |
|  | putative outer membrane protein; RatA precursor homolog | pm : putative membrane component | 5.13 : Virulence associated ; | 15.9 : Pathogenesis ; | ECS88_2682 |
|  | putative exported Zn-dependent hydrolase | pe : putative enzyme |  |  | ECS88_3008 |
|  | putative protein with flavodoxin fold | pf : putative factor |  |  | ECS88_3009 |
|  | 2-hydroxyacid dehydrogenase | e : enzyme |  | 6 : Energy metabolism ; | ECS88_3107 |
|  | putative phosphosugar isomerase | pe : putative enzyme | 1.1.1 : Carbohydrates/Carbon compounds ; | 6 : Energy metabolism ; | ECS88_3108 |
|  | putative aminotransferase | pe : putative enzyme |  | 18.1 : Enzymes of unknown specificity ; | ECS88_3109 |
|  | Phosphotransferase system system, maltose and glucose-specific IIABC component | t : transporter | 4.4.A : Phosphotransferase Systems (PEP-dependent PTS) ; | 13.2 : PTS ; | ECS88_3110 |
|  | putative transcription antiterminator | pr : putative regulator | 3.1.2 : Transcriptional level ; | 12.1 : DNA interactions ; | ECS88_3111 |
|  | putative regulator | pr : putative regulator | 3.1.2.3 : Repressor ; 7.1 : Cytoplasm ; | 12.1 : DNA interactions ; | ECS88_3397 |
|  | putative Fructuronate reductase | pe : putative enzyme |  | 6 : Energy metabolism ; | ECS88_3398 |
|  | putative L-threonine 3-dehydrogenase | pe : putative enzyme |  | 6.2 : Amino acids and amines ; | ECS88_3399 |
|  | putative Ureidoglycolate dehydrogenase | pe : putative enzyme | 1.1.5.2 : Ethanol degradation ; | 5.7 : Nitrogen metabolism | ECS88_3400 |
|  | putative c4-dicarboxylate transport system binding protein | pt : putative transporter | 6.1 : Membrane: | 7 : Transport and binding proteins ; | ECS88_3401 |
|  | putative membrane protein. | pm : putative membrane component |  | 18 : Unknown function ; | ECS88_3402 |
|  | putative C4-dicarboxylate permease | pt : putative transporter | 6.1 : Membrane ; | 7 : Transport and binding proteins ; | ECS88_3403 |
|  | conserved hypothetical protein | o : ORF of unknown function |  | 18 : Unknown function ; | ECS88_3996 |
|  | conserved hypothetical protein | o : ORF of unknown function |  | 18 : Unknown function ; | ECS88_4691 |
|  | conserved hypothetical protein | o : ORF of unknown function |  | 18 : Unknown function ; | ECS88_4083 |
|  | conserved hypothetical protein; putative sugar phosphate isomerase involved in capsule formation | o : ORF of unknown function |  | 18 : Unknown function ; | ECS88_4262 |
| *ptsG* | fused glucose-specific PTS enzymes: IIBcomponent and IIC component | t : transporter | 1.1.1 : Carbohydrates/Carbon compounds ; | 6 : Energy metabolism ; | ECS88_4263 |
|  | transketolase 1, thiamin-binding | e : enzyme |  | 2.2 : Nucleotide and nucleoside interconversions | ECS88_4264 |
|  | conserved hypothetical protein | o : ORF of unknown function |  | 18 : Unknown function ; | ECS88_4265 |
|  | putative transcritional regulator | pr : putative regulator |  | 12 : Regulatory functions ; | ECS88_4266 |
|  | putative permease; putative Purine-cytosine permease and related proteins | pe : putative enzyme |  | 7 : Transport and binding proteins ; | ECS88_4267 |
|  | conserved hypothetical protein | o : ORF of unknown function |  | 18 : Unknown function ; | ECS88_4268 |
|  | Putative Carbamate kinase | pe : putative enzyme |  | 5 : Central intermediary metabolism ; | ECS88_4269 |
|  | conserved hypothetical protein | o : ORF of unknown function |  | 18 : Unknown function ; | ECS88_4270 |
|  | Putative enzyme, Putative Succinyl-CoA synthetase, alpha subunit | pe : putative enzyme |  | 18.1 : Enzymes of unknown specificity ; | ECS88_4271 |
|  | conserved hypothetical protein | o : ORF of unknown function |  | 18 : Unknown function ; | ECS88_4272 |
|  | conserved hypothetical protein; putative Isochorismate hydrolase | o : ORF of unknown function |  | 18 : Unknown function ; | ECS88_4273 |
|  | phospho acidic carbohydrate aldolase | e : enzyme |  | 6.11 : Sugars ; | ECS88_4279 |
| *sucA* | 2-oxoglutarate dehydrogenase E1 component (EC 1.2.4.2) (Alpha- ketoglutarate dehydrogenase) | e : enzyme |  |  | ECS88_4534 |
| *sucB* | Dihydrolipoyllysine-residue succinyltransferase component of 2- oxoglutarate dehydrogenase complex (EC 2.3.1.61) (E2) (Dihydrolipoamide succinyltransferase component of 2-oxoglutarate dehydrogenase complex) ) | e : enzyme |  | 6.12 : TCA cycle ; | ECS88_4535 |
|  | putative dihydrolipoyl dehydrogenase; E3 component of dehydrogenase complex | pe : putative enzyme |  | 6.12 : TCA cycle ; | ECS88_4536 |
| *sucC* | Succinyl-CoA synthetase beta chain (EC 6.2.1.5) (SCS-beta) | e : enzyme |  | 6.12 : TCA cycle ; | ECS88_4537 |
| *sucD* | Succinyl-CoA synthetase alpha chain (EC 6.2.1.5) (SCS-alpha) | e : enzyme |  | 6.12 : TCA cycle ; | ECS88_4538 |
|  | putative membrane protein; putative transporter | pt : putative transporter |  | 18 : Unknown function ; | ECS88_4539 |
|  | Putative malate/L-lactate dehydrogenases | pe : putative enzyme |  | 6 : Energy metabolism ; | ECS88_4540 |
|  | putative C4-dicarboxylate transport sensor protein (dctB-like) | prc : putative receptor |  | 7 : Transport and binding proteins ; | ECS88_4543 |
|  | conserved hypothetical protein; putative membrane protein | o : ORF of unknown function |  | 18 : Unknown function ; | ECS88_4558 |
|  | conserved hypothetical protein; putative membrane protein | o : ORF of unknown function |  | 18 : Unknown function ; | ECS88_4559 |
|  | putative ABC transporter ATP-binding protein (yddO) | pt : putative transporter |  | 7 : Transport and binding proteins ; | ECS88_4572 |
|  | putative dipeptide/oligopeptide/nickel transport system, ATPase component | pt : putative transporter |  | 7 : Transport and binding proteins ; | ECS88_4573 |
|  | putative dipeptide/nickel transporter, Inner membrane permease subunit (yddQ) | pt : putative transporter |  | 7 : Transport and binding proteins ; | ECS88_4574 |
|  | putative dipeptide/nickel transporter, Inner membrane permease subunit precursor (yddR) | pt : putative transporter |  | 7 : Transport and binding proteins ; | ECS88_4575 |
|  | putative ABC-type dipeptide transport system, periplasmic component | pt : putative transporter |  | 7 : Transport and binding proteins ; | ECS88_4576 |
|  | conserved hypothetical protein | o : ORF of unknown function |  | 18 : Unknown function ; | ECS88_4790 |

Genes absent in the B2 group strains

| **Gene** | **Product** | **ProductType** | **Roles** | **BioProcess** | **Locus_tag** (from K12 strain) |
| --- | --- | --- | --- | --- | --- |
| *coda* | cytosine deaminase | e : enzyme | 1.7.33.4 : Salvage pathways of pyrimidine ribonucleotides | 2.2 : Nucleotide and nucleoside interconversions ; | ECK0334 |
| *cynR* | DNA-binding transcriptional dual regulator | r : regulator | 1.7.1 : Unassigned reversible reactions | 5 : Central intermediary metabolism | ECK0335 |
| *cynT* | carbonic anhydrase | e : enzyme | 1.7.24 : Cyanate catabolism ; | 15.5 : Detoxification ; | ECK0336 |
| *cynS* | cyanate aminohydrolase | e : enzyme | 1.7.24 : Cyanate catabolism ; | 5.7 : Nitrogen metabolism ; 15.5 : Detoxification ; | ECK0337 |
| *cynX* | putative cyanate transporter | pt : putative transporter | 1.7.24 : Cyanate catabolism | 7 : Transport and binding proteins | ECK0338 |
| *sfmA* | putative fimbrial-like adhesin protein | ps : putative structure | 6.5 : Pilus ; | 16.8 : Protect ; 16.5 : Explore | ECK0523 |
| *sfmC* | pilin chaperone, periplasmic | f : factor | 2.3.4 : Chaperoning, folding | 11.3 : Protein folding and stabilization ; | ECK0524 |
| *sfmD* | putative outer membrane export usher protein | pf : putative factor | 6.1 : Membrane ; | 16.1 : Circulate ; 16.13 : Shape ; | ECK0525 |
| *sfmH* | putative fimbrial-like adhesin protein | ps : putative structure | 1.6.12 : Flagella ; | 14.1 : Surface structures ; | ECK0526 |
| *sfmF* | putative fimbrial-like adhesin protein | ps : putative structure | 6.5 : Pilus ; | 14.1 : Surface structures ; | ECK0527 |
| *fimZ* | DNA-binding transcriptional regulator, isolated component of a two-component regulator system | r : regulator | 2.2.2 : Transcription related | 9 : Transcription ; 12.1 | ECK0528 |
| *ybeF* | putative DNA-binding transcriptional regulator, LysR-type | pr : putative regulator | 2.2.2 : Transcription related | 16.6 : Maintain ; 9 : Transcription | ECK0622 |
| *ybiU* | hypothetical protein | o : ORF of unknown function |  | 18 : Unknown function ; | ECK0811 |
| *ycaM* | putative transporter | pt : putative transporter | 4.2.A.3 : The Amino Acid-Polyamine-Choline (APC) Family | 16.3 : Control ; 7 : Transport and binding proteins ; | ECK0890 |
| *puuP* | putrescine importer | t : transporter | 4.2.A.3 : The Amino Acid-Polyamine-Choline (APC) Family | 7 : Transport and binding proteins ; | ECK1291 |
| *puuA* | gamma-Glu-putrescine synthase | e : enzyme | 1.5.1.2 : Glutamine ; | 1.3 : Glutamate family ; 5.7 : Nitrogen metabolism | ECK1292 |
| *puuD* | gamma-Glu-GABA hydrolase | e : enzyme | 1.1.4 : Amines ; | 6.2 : Amino acids and amines ; | ECK1293 |
| *puuR* | DNA-binding transcriptional repressor | r : regulator |  | 12 : Regulatory functions ; | ECK1294 |
| *puuC* | gamma-Glu-gamma-aminobutyraldehyde dehydrogenase, NAD(P)H-dependent | e : enzyme | 1.7.32.2 : Putrescine degradation II ; | 6.2 : Amino acids and amines ; | ECK1295 |
| *puub* | gamma-Glu-putrescine oxidase, FAD/NAD(P)-binding | e : enzyme | 1.7.32.2 : Putrescine degradation II ; | 6.2 : Amino acids and amines ; | ECK1296 |
| *puuE* | GABA aminotransferase, PLP-dependent | e : enzyme | 1.5.3.6 : Pyridoxine (vitamin B6) ; | 4.8 : Pyridoxine ; 6 : Energy metabolism ; | ECK1297 |
| *abgA* | putative peptidase, para-aminobenzoyl-glutamate utilization protein | pe : putative enzyme | 1.1.1 : Carbohydrates/Carbon compounds ; | 16.3 : Control ; 6 : Energy metabolism ; | ECK1334 |
| *abgR* | DNA-binding transcriptional regulator | r : regulator | 1.1.1 : Carbohydrates/Carbon compounds ; Repressor ; | 16.1 : Circulate ; 6 : Energy metabolism ; | ECK1335 |
| *ydbD* | hypothetical protein | o : ORF of unknown function |  | 18 : Unknown function ; | ECK1400 |
| *ddpF* | D-ala-D-ala transporter subunit ; ATP-binding component of ABC superfamily | t : transporter | 4.3.A.1.a : ATP binding | 7.1 : Amino acids, peptides and amines ; | ECK1477 |
| *ddpD* | D-ala-D-ala transporter subunit ; ATP-binding component of ABC superfamily | t : transporter | 4.3.A.1.a : ATP binding component ; | 7.1 : Amino acids, peptides and amines ; | ECK1478 |
| *ddpC* | D-ala-D-ala transporter subunit ; membrane component of ABC superfamily | t : transporter | 4.3.A.1.m : membrane component ; | 7.1 : Amino acids, peptides and amines ; | ECK1479 |
| *ddpB* | D-ala-D-ala transporter subunit ; membrane component of ABC superfamily | t : transporter | 4.3.A.1.m : membrane component ; | 7.1 : Amino acids, peptides and amines ; | ECK1480 |
| *ddpA* | D-ala-D-a la transporter subunit ; periplasmic-binding component of ABC superfamily | t : transporter | 4.3.A.1.p : periplasmic binding component ; | 7.1 : Amino acids, peptides and amines ; | ECK1481 |
| *ddpX* | D-ala-D-ala dipeptidase, Zn-dependent | e : enzyme | 1.1.5 : Others ; 5.5.1 : Osmotic pressure ; | 6 : Energy metabolism ; | ECK1482 |
| *lsrB* | AI2 transporter ; periplasmic-binding component of ABC superfamily | t : transporter | 4.3.A.1.p : periplasmic binding component | 7.3 : Carbohydrates, organic alcohols, and acids ; | ECK1509 |
| *lsrF* | putative aldolase | pe : putative enzyme |  | 16.11 : Scavenge (Catabolism) ; | ECK1510 |
| *lsrG* | conserved hypothetical protein | o : ORF of unknown function |  | 18 : Unknown function ; | ECK1511 |
| *ydeI* | conserved hypothetical protein | o : ORF of unknown function |  | 18 : Unknown function ; | ECK1529 |
| *Lhr* | putative ATP-dependent helicase | pe : putative enzyme | 2.1.1 : DNA replication ; | 16.1 : Circulate ; | ECK1649 |
| *yeaT* | putative DNA-binding transcriptional regulator | pr : putative regulator | 2.2.2 : Transcription related ; | 9 : Transcription | ECK1797 |
| *yeaU* | putative tartrate dehydrogenase | pe : putative enzyme | 1.3.5 : Fermentation ; | 16.1 : Circulate ; 6.7 : Fermentation ; | ECK1798 |
| *yeaV* | putative transporter | pt : putative transporter | 4.2.A.15 : The Betaine/Carnitine/Choline Transporter (BCCT) Family ;; | 7 : Transport and binding proteins ; | ECK1799 |
| *yeaW* | putative 2Fe-2S cluster-containing dioxygenase subunit | pc : putative carrier |  | 18.1 : Enzymes of unknown specificity ; | ECK1800 |
| *yeaX* | putative dioxygenase subunit | pe : putative enzyme |  | 18.1 : Enzymes of unknown specificity ; | ECK1801 |
| *elaD* | putative enzyme | pe : putative enzyme |  | 18.1 : Enzymes of unknown specificity ; | ECK2263 |
| *yfeT* | putative DNA-binding transcriptional regulator | pr : putative regulator |  | 16.3 : Control ; | ECK2422 |
| *murQ* | D-lactyl ether N-acetylmuramic-6-phosphate acid etherase; beta-lactamase family | e : enzyme | 1.1.1 : Carbohydrates/Carbon compounds ; | 16.3 : Control ; 6 : Energy metabolism ; | ECK2425 |
| *hyfA* | hydrogenase 4, 4Fe-4S subunit | c : carrier | 1.3.7 : Anaerobic respiration ; | 6.3 : Anaerobic ; | ECK2477 |
| *hyfB* | hydrogenase 4, membrane subunit | m : membrane component | 1.3.7 : Anaerobic respiration | 6.3 : Anaerobic ; | ECK2478 |
| *hyfC* | hydrogenase 4, membrane subunit | m : membrane component | 1.3.7 : Anaerobic respiration ; | 6.3 : Anaerobic ; | ECK2479 |
| *hyfD* | hydrogenase 4, membrane subunit | m : membrane component | 1.3.7 : Anaerobic respiration ; | 6.3 : Anaerobic ; | ECK2480 |
| *hyfE* | hydrogenase 4, membrane subunit | m : membrane component | 1.3.7 : Anaerobic respiration | 6.3 : Anaerobic ; | ECK2481 |
| *hyfF* | hydrogenase 4, membrane subunit | m : membrane component | 1.3.7 : Anaerobic respiration | 6.3 : Anaerobic ; | ECK2482 |
| *hyfG* | hydrogenase 4, subunit | c : carrier | 1.3.7 : Anaerobic respiration ; | 6.3 : Anaerobic ; | ECK2483 |
| *hyfH* | hydrogenase 4, Fe-S subunit | c : carrier | 1.3.7 : Anaerobic respiration ; | 6.3 : Anaerobic ; | ECK2484 |
| *hyfI* | hydrogenase 4, Fe-S subunit | c : carrier | 1.3.7 : Anaerobic respiration ; | 6.3 : Anaerobic ; | ECK2485 |
| *hyfJ* | putative processing element hydrogenase 4 | pf : putative factor | 2.3 : Protein related ; | 18.1 : Enzymes of unknown specificity ; | ECK2486 |
| *hcaR* | DNA-binding transcriptional activator of 3-phenylpropionic acid catabolism | r : regulator | 1.1.1 : Carbohydrates/Carbon compounds; | 6 : Energy metabolism ; | ECK2534 |
| *hcaE* | 3-phenylpropionate dioxygenase, large (alpha) subunit | e : enzyme | 1.1.2.2 : 3-phenylpropionate and 3-(3-hydroxyphenyl)propionate degradation ; | 6 : Energy metabolism ; | ECK2535 |
| *hcaF* | 3-phenylpropionate dioxygenase, small (beta) subunit | e : enzyme | 1.1.2.2 : 3-phenylpropionate and 3-(3-hydroxyphenyl)propionate degradation ; | 6 : Energy metabolism ; | ECK2536 |
| *hcaC* | 3-phenylpropionate dioxygenase, putative ferredoxin subunit | pc : putative carrier | 1.1.2.2 : 3-phenylpropionate and 3-(3-hydroxyphenyl)propionate degradation ; | 6 : Energy metabolism ; | ECK2537 |
| *hcaB* | 2,3-dihydroxy-2,3-dihydrophenylpropionate dehydrogenase | e : enzyme | 1.1.2.2 : 3-phenylpropionate and 3-(3-hydroxyphenyl)propionate degradation ; | 6 : Energy metabolism ; | ECK2538 |
| *yqeH* | conserved hypothetical protein; putative bipartite regulator domain | o : ORF of unknown function |  | 18 : Unknown function ; | ECK2844 |
| *yqeI* | putative transcriptional regulator | pr : putative regulator |  | 12 : Regulatory functions ; | ECK2845 |
| *yqeJ* | hypothetical protein | o : ORF of unknown function |  | 18 : Unknown function ; | ECK2846 |
| *ygeF* | hypothetical protein | o : ORF of unknown function |  | 18 : Unknown function ; | ECK2848 |
| *ygeG* | putative chaperone | pc : putative carrier |  | 11.3 : Protein folding and stabilization ; | ECK2849 |
| *ygeH* | putative transcriptional regulator | pr : putative regulator | 5.10 : Defense/survival ; | 16.1 : Circulate ; 16.4 : Excrete ; | ECK2850 |
| *ygeI* | hypothetical protein | o : ORF of unknown function |  | 18 : Unknown function ; | ECK2851 |
| *argK* | membrane ATPase/protein kinase | t : transporter | 6.1 : Membrane ; 7.3 : Inner membrane ; | 7 : Transport and binding proteins ; | ECK2914 |
| *ygfG* | methylmalonyl-CoA decarboxylase, biotin-independent | e : enzyme | 1.7.1 : Unassigned reversible reactions ; 1.7.39 : Conversion of succinate to propionate ; | 5 : Central intermediary metabolism ; | ECK2915 |
| *ygfH* | propionyl-CoA:succinate-CoA transferase | e : enzyme | 1.7.1 : Unassigned reversible reactions ; 1.7.39 : Conversion of succinate to propionate ; | 5 : Central intermediary metabolism ; | ECK2916 |
| *yggF* | putative hexoseP phosphatase | pe : putative enzyme |  | 6.11 : Sugars ; | ECK2926 |
| *yhaI* | conserved hypothetical protein; putative inner membrane protein | pm : putative membrane component | 6.1 : Membrane ; | 18 : Unknown function ; | ECK3095 |
| *yhaB* | hypothetical protein | o : ORF of unknown function |  | 18 : Unknown function ; | ECK3109 |
| *yhaC* | hypothetical protein | o : ORF of unknown function |  | 18 : Unknown function ; | ECK3110 |
| *dcuD* | putative transporter | pt : putative transporter | 4.2.A.61 : The C4-dicarboxylate Uptake C (DcuC) Family | 7 : Transport and binding proteins ; | ECK3216 |
| *arsR* | DNA-binding transcriptional repressor | r : regulator | 2.2.2 : Transcription related | 9 : Transcription ; 12.1 : DNA interactions ; | ECK3486 |
| *arsB* | arsenite/antimonite transporter | t : transporter | 4.3.A.4 : The Arsenite-Antimonite (Ars) Efflux Family ; | 7.2 : Anions ; 15.5 : Detoxification ; | ECK3487 |
| *glvC* | arbutin sp ecific enzyme IIC component of PTS | t : transporter | 4.4.A.1 : The PTS Glucose-Glucoside (Glc) Family ;; | 7.3 : Carbohydrates, organic alcohols, and acids ; | ECK3675 |
| *yidP* | putative DNA-binding transcriptional regulator | pr : putative regulator | 2.2.2 : Transcription related ; | 9 : Transcription ; 12.1 : DNA interactions ; | ECK3676 |
| *yiiF* | conserved hypothetical protein | o : ORF of unknown function |  | 18 : Unknown function ; | ECK3883 |
| *yjbI* | conserved hypothetical protein | o : ORF of unknown function |  | 18 : Unknown function ; | ECK4030 |
| *melB* | melibiose:sodium symporter | t : transporter | 1.1.1 : Carbohydrates/Carbon compounds | 6 : Energy metabolism ; | ECK4113 |
| *yjfZ* | hypothetical protein | o : ORF of unknown function |  | 18 : Unknown function ; | ECK4200 |

Genes present in the B2 group strains virulent in the mouse model of septicaemia

| **Gene** | **Product** | **ProductType** | **Roles** | **BioProcess** | **Locus_tag** (from S88 strain) |
| --- | --- | --- | --- | --- | --- |
|  | CP4-like integrase | h : extrachromosomal origin | 8.1 : Prophage genes and phage related functions ; | 17.2 : Prophage functions ; | ECS88_0279 |
|  | putative transcription regulator; LuxR-type HTH domain | pr : putative regulator |  |  | ECS88_0315 |
|  | Putative DNA recombinase similar to Type 1 fimbriae Regulatory proteins | pe : putative enzyme |  |  | ECS88_0317 |
|  | conserved hypothetical protein | o : ORF of unknown function |  |  | ECS88_0318 |
|  | conserved hypothetical protein; putative membrane protein (part 1) | o : ORF of unknown function |  |  | ECS88_0705+6 |
|  | Conserved hypothetical protein | o : ORF of unknown function |  |  | ECS88_0707 |
|  | Putative Glycosyl hydrolase, BNR repeat | pe : putative enzyme |  |  | ECS88_0708 |
| *dapA* | putative dihydrodipicolinate synthase | pe : putative enzyme | 1.5.1.7 : Lysine, diaminopimelate ; | 1.2 : Aspartate family ; | ECS88_0711 |
|  | putative alcohol dehydrogenase | pe : putative enzyme |  |  | ECS88_0712 |
|  | conserved hypothetical protein | o : ORF of unknown function |  | 18 : Unknown function ; | ECS88_0713 |
|  | Putative pyridoxine phosphate biosynthetic protein (PdxA-like protein) | pe : putative enzyme |  |  | ECS88_0714 |
|  | putative transcriptional regulator, DeoR-family | pr : putative regulator |  | 9 : Transcription ; 12.1 : DNA interactions ; | ECS88_0715 |
|  | conserved hypothetical protein | o : ORF of unknown function |  |  | ECS88_0896 |
| *nmpC* | putative outer membrane porin | h : extrachromosomal origin | 7.4 : Outer membrane ; 8.1 : Prophage genes and phage related functions ; |  | ECS88_1183 |
| *yddV* | putative diguanylate cyclase YddV (partial) | pe : putative enzyme |  |  | ECS88_1578 |
|  | conserved hypothetical protein | o : ORF of unknown function |  |  | ECS88_3085 |
|  | conserved hypothetical protein; putative membrane associated protein | o : ORF of unknown function |  |  | ECS88_3086 |
|  | conserved hypothetical protein; putative membrane protein | o : ORF of unknown function |  |  | ECS88_3087 |
|  | Conserved hypothetical protein | o : ORF of unknown function |  |  | ECS88_3088 |
|  | Chaperone clpB | f : factor | 2.3.4 : Chaperoning, folding ; 7.1 : Cytoplasm ; |  | ECS88_3089 |
|  | conserved hypothetical protein | o : ORF of unknown function |  |  | ECS88_3090 |
|  | conserved hypothetical protein; putative membrane protein | o : ORF of unknown function |  |  | ECS88_3097 |
|  | conserved hypothetical protein | o : ORF of unknown function |  |  | ECS88_3098 |
|  | conserved hypothetical protein | o : ORF of unknown function |  |  | ECS88_3102 |
|  | conserved hypothetical protein | o : ORF of unknown function |  |  | ECS88_3103 |
|  | conserved hypothetical protein | o : ORF of unknown function |  |  | ECS88_3104 |
|  | conserved hypothetical protein | o : ORF of unknown function |  |  | ECS88_3105 |
|  | conserved hypothetical protein | o : ORF of unknown function |  |  | ECS88_3106 |
|  | conserved hypothetical protein | o : ORF of unknown function |  |  | ECS88_4259 |
|  | putative transporter | pt : putative transporter |  |  | ECS88_4531 |
|  | putative transcriptional regulator | pr : putative regulator |  |  | ECS88_4532 |

Genes absent in the B2 group strains virulent in the mouse model of septicaemia

| **Gene** | **Product** | **ProductType** | **Roles** | **BioProcess** | **Locus_tag** (from K12 strain) |
| --- | --- | --- | --- | --- | --- |
| *mhpR* | DNA-binding transcriptional activator, 3HPP-binding | r : regulator | 1.1.2 : Fatty acids (fatty acid oxidation) ; | 3.2 : Degradation ; | ECK0343 |
| *mhpA* | 3-(3-hydroxyphenyl)propionate hydroxylase | e : enzyme | 1.1.2.2 : 3-phenylpropionate and 3-(3-hydroxyphenyl)propionate degradation ; | 6 : Energy metabolism ; | ECK0344 |
| *mhpB* | 2,3-dihydroxyphenylpropionate 1,2-dioxygenase | e : enzyme | 1.1.2.2 : 3-phenylpropionate and 3-(3-hydroxyphenyl)propionate degradation ; | 6 : Energy metabolism ; | ECK0345 |
| *mhpC* | 2-hydroxy-6-ketonona-2,4-dienedioic acid hydrolase | e : enzyme | 1.1.2.2 : 3-phenylpropionate and 3-(3-hydroxyphenyl)propionate degradation ; | 6 : Energy metabolism ; | ECK0346 |
| *mhpD* | 2-keto-4-pentenoate hydratase | e : enzyme | 1.1.2.2 : 3-phenylpropionate and 3-(3-hydroxyphenyl)propionate degradation ; | 6 : Energy metabolism ; | ECK0347 |
| *mhpF* | acetaldehyde-CoA dehydrogenase II, NAD-binding | e : enzyme | 1.1.2.2 : 3-phenylpropionate and 3-(3-hydroxyphenyl)propionate degradation ; | 6 : Energy metabolism ; | ECK0348 |
| *mhpE* | 4-hyroxy-2-oxovalerate/4-hydroxy-2-oxopentanoic acid aldolase, class I | e : enzyme | 1.1.2.2 : 3-phenylpropionate and 3-(3-hydroxyphenyl)propionate degradation ; | 6 : Energy metabolism ; | ECK0349 |
| *mhpT* | hydroxy-aromatic acid transporter | t : transporter | 1.1.2 : Fatty acids (fatty acid oxidation) | 3.2 : Degradation | ECK0350 |

Genes present in the strains with ExPEC phenotype

| **Gene** | **Product** | **ProductType** | **Roles** | **BioProcess** | **Locus_tag** (from S88 strain) |
| --- | --- | --- | --- | --- | --- |
|  | putative transcriptional regulator, lysR family | pr : putative regulator | 3.1.2 : Transcriptional level ; | 12.1 : DNA interactions ; | ECS88_0297 |
|  | putative Pyridoxine 4-dehydrogenase | pe : putative enzyme |  | 18.1 : Enzymes of unknown specificity ; | ECS88_0298 |
|  | putative reductase (EC 1.1.-.-) | pe : putative enzyme |  | 18.1 : Enzymes of unknown specificity ; | ECS88_0299 |
|  | Putative flagellin-like structural protein similar to yaiT; putative exported protein | ps : putative structure |  | 14.1 : Surface structures ; | ECS88_0367 |
| *papI* | pap operon regulatory protein PapI | r : regulator | 2.2.2 : Transcription related ; 3.1.2 : Transcriptional level ; | 9 : Transcription ; 12.1 : DNA interactions ; | ECS88_3267 |
| *papB* | pap operon regulatory protein PapB | r : regulator | 1.6.13 : Fimbria, pili, curli ; 3.1.2 : Transcriptional level ; | 12.1 : DNA interactions ; | ECS88_3266 |
|  | Putative HTH-type transcriptional regulator | pr : putative regulator |  | 9 : Transcription ; 12.1 : DNA interactions ; | ECS88_4713 |
|  | conserved hypothetical protein | o : ORF of unknown function |  | 18 : Unknown function ; | ECS88_0593 |
| *papF* | Fimbrial adapter PapF precursor | s : structure | 6.5 : Pilus ; 1.6.13 : Fimbria, pili, curli ; | 14.1 : Surface structures ; | ECS88_3258 |
| *papE* | Fimbrial tip protein PapE | s : structure | 1.6.13 : Fimbria, pili, curli ; 6.5 : Pilus ; | 14.1 : Surface structures ; | ECS88_3259 |
| *papK* | Fimbrial adapter PapK precursor | s : structure | 1.6.13 : Fimbria, pili, curli ; 6.5 : Pilus ; | 14.1 : Surface structures ; | ECS88_3260 |
| *papJ* | Protein PapJ precursor, P pilus assembly | f : factor | 2.3.4 : Chaperoning, folding ; 7.2 : Periplasmic space ; | 11.3 : Protein folding and stabilization ; | ECS88_3261 |
| *papD* | Chaperone protein PapD precursor | f : factor | 2.3.4 : Chaperoning, folding ; 7.2 : Periplasmic space ; | 11.3 : Protein folding and stabilization ; | ECS88_3262 |
| *papC* | Outer membrane usher protein PapC precursor | f : factor | 6.1 : Membrane ; 7.4 : Outer membrane ; | 14 : Cell envelope ; | ECS88_3263 |
| *papH* | minor pilin protein PapH | s : structure | 1.6.13 : Fimbria, pili, curli ; 6.5 : Pilus ; | 14.1 : Surface structures ; | ECS88_3264 |
|  | conserved hypothetical protein | o : ORF of unknown function |  | 18 : Unknown function ; | ECS88_4395 |

Genes absent in the strains with ExPEC phenotype

| **Gene** | **Product** | **ProductType** | **Roles** | **BioProcess** | **Locus_tag** **(**from K12 strain) |
| --- | --- | --- | --- | --- | --- |
| *yodB* | putative cytochrome | pc : putative carrier | 1.6.15.1 : Cytochromes ; 6.1 : Membrane ; | 16.1 : Circulate ; 6.5 : Electron transport ; | ECK1970 |

Genes present in *Shigella* strains (Virulence plasmid excluded)

| **Gene** | **Product** | **ProductType** | **Roles** | **BioProcess** | **Locus_tag** (from S. dysenteria) |
| --- | --- | --- | --- | --- | --- |
|  | putative tail fiber assembly protein |  |  | 17.2 : Prophage functions ; | SDY1066 |
|  | putative phage tail fiber protein |  |  | 17.2 : Prophage functions ; | SDY0772 |
|  | 60 kDa antigen |  |  | 17.2 : Prophage functions ; 15.9 : Pathogenesis | SDY2753 |
|  | conserved hypothetical protein |  |  | 18 : Unknown function ; | SDY3714 |
|  | 65.4 kDa antigen |  |  | 18 : Unknown function ; 15.9 : Pathogenesis ; | SDY0834 |
|  | conserved hypothetical protein |  |  | 18 : Unknown function ; | SDY0773 |
|  | hypothetical protein |  |  | 18 : Unknown function ; | SHISD5335 |
|  | conserved hypothetical protein |  |  | 18 : Unknown function ; | SHISD4439 |

Genes absent in *Shigella* strains

| **Gene** | **Product** | **ProductType** | **Roles** | **BioProcess** | **Locus_tag** (from K12 strain) |
| --- | --- | --- | --- | --- | --- |
| *yaaJ* | putative amino acid sodium/proton transporter | pt : putative transporter | 4.2.A.25 : The Alanine/Glycine:Cation symporter (AGCS) Family ; | 7 : Transport and binding proteins ; | ECK0007 |
| *yagX* | conserved hypothetical protein | o : ORF of unknown function |  | 18 : Unknown function ; | ECK0290 |
| *yahO* | hypothetical protein | o : ORF of unknown function |  | 18 : Unknown function ; | ECK0327 |
| *prpR* | DNA-binding transcriptional activator | r : regulator | 2.2.2 : Transcription related | 9 : Transcription ; 12.1 : DNA interactions ; | ECK0328 |
| *prpB* | 2-methylisocitrate lyase | e : enzyme | 1.1.2.3 : Propionate degradation | 6 : Energy metabolism ; | ECK0329 |
| *prpC* | 2-methylcitrate synthase | e : enzyme | 1.1.2.3 : Propionate degradation ; | 6 : Energy metabolism ; | ECK0330 |
| *prpD* | 2-methylcitrate dehydratase | e : enzyme | 1.1.2.3 : Propionate degradation ; | 6 : Energy metabolism ; | ECK0331 |
| *prpE* | propionyl-CoA synthetase | pe : putative enzyme | 1.1.2.3 : Propionate degradation | 6 : Energy metabolism ; | ECK0332 |
| *codB* | cytosine transporter | t : transporter | 1.7.33 : Nucleotide and nucleoside conversions ; | 2.2 : Nucleotide and nucleoside interconversions ; | ECK0333 |
| *codA* | cytosine deaminase | e : enzyme | 1.7.33.4 : Salvage pathways of pyrimidine ribonucleotides ; | 2.2 : Nucleotide and nucleoside interconversions ; | ECK0334 |
| *lacY* | lactose/galactose transporter | t : transporter | 1.1.1 : Carbohydrates/Carbon compounds | 6 : Energy metabolism | ECK0340 |
| *ybbY* | putative uracil/xanthine transporter | pt : putative transporter | 4.9.B : Putative uncharacterized transport protein | 7 : Transport and binding proteins ; | ECK0506 |
| *allD* | ureidoglycolate dehydrogenase | e : enzyme | 1.1.5.2 : Ethanol degradation | 5.7 : Nitrogen metabolism; | ECK0510 |
| *fiu* | putative TonB-dependent iron outer membrane transporter | pt : putative transporter | 5.5.7 : Fe aquisition | 15.10 : Adaptations to atypical conditions ; | ECK0794 |
| *ydbC* | putative aldo/keto reductase, NAD(P)-binding | pe : putative enzyme |  | 18.1 : Enzymes of unknown specificity ; | ECK1399 |
| *speG* | spermidine N1-acetyltransferase | e : enzyme | 1.7.14 : Polyamine biosynthesis ; | 5.4 : Polyamine biosynthesis ; | ECK1579 |
| *ydiF* | short chain acyl-CoA transferase: fused alpha subunit ; beta subunit | e : enzyme | 1.1.2 : Fatty acids (fatty acid oxidation) ; | 3.2 : Degradation ; | ECK1692 |
| *ynjC* | fused putative transporter subunits of ABC superfamily: membrane components | pt : putative transporter | 4.3.A.1.m : membrane component | 7 : Transport and binding proteins ; | ECK1753 |
| *yecE* | conserved hypothetical protein | o : ORF of unknown function |  | 18 : Unknown function ; | ECK1869 |
| *yfaL* | adhesin | f : factor | 5.13 : Virulence associated ; | 15.9 : Pathogenesis ; | ECK2225 |
| *ygfK* | putative oxidoreductase, Fe-S subunit | pc : putative carrier |  | 18.1 : Enzymes of unknown specificity ; | ECK2874 |
| *ygfO* | putative transporter | pt : putative transporter | 4.2.A.40 : The Nucleobase:Cation Symporter-2 (NCS2) Family | 7 : Transport and binding proteins ; | ECK2878 |
| *guaD* | guanine deaminase | e : enzyme | 1.7.1 : Unassigned reversible reactions | 2.2 : Nucleotide and nucleoside interconversions ; | ECK2879 |
| *ygfQ* | putative transporter | pt : putative transporter | 4 : Transport ; 6.1 : Membrane ; | 7 : Transport and binding proteins ; | ECK2880 |
| *agaS* | tagatose-6-phosphate ketose/aldose isomerase | e : enzyme | 1.7.1 : Unassigned reversible reactions ; | 5 : Central intermediary metabolism ; | ECK3124 |
| *kbaY* | tagatose 6-phosphate aldolase 1, kbaY subunit | e : enzyme | 1.1.1.12 : Galactitol catabolism ; | 6.11 : Sugars ; | ECK3125 |
| *agaB* | N-acetylgalactosamine-specific enzyme IIB component of PTS | t : transporter | 4.4.A.6 : The PTS Mannose-Fructose-Sorbose (Man) Family | 7.1 : Amino acids, peptides and amines | ECK3126 |
| *aapQ* | amino-acid transporter subunit ; membrane component of ABC superfamily | t : transporter | 4.3.A.1.m : membrane component | 7 : Transport and binding proteins ; | ECK3256 |
| *rbsB* | D-ribose transporter subunit ; periplasmic-binding compoent of ABC superfamily | t : transporter | 1.1.1 : Carbohydrates/Carbon compounds ; 4.3.A.1.p : periplasmic binding component ; | 6 : Energy metabolism ; | ECK3745 |
| *cadB* | putative lysine/cadaverine transporter | pt : putative transporter | 1.5.1.7 : Lysine, diaminopimelate ; 4.2.A.3 : The Amino Acid-Polyamine-Choline (APC) Family ; | 1.2 : Aspartate family | ECK4126 |
| *cadC* | DNA-binding transcriptional activator | r : regulator | 1.1.3 : Amino acids ; 2.2.2 : Transcription related ; 3.1.2.2 : Activator ; 3.3.1 : Operon (regulation of one operon) ; 7.1 : Cytoplasm ; | 6.2 : Amino acids and amines | ECK4127 |
| *yjfI* | conserved hypothetical protein | o : ORF of unknown function |  | 18 : Unknown function ; | ECK4177 |
